# Acyl-CoA-binding protein (ACBP): a poor-prognosis biomarker in sepsis and a target for disease mitigation

**DOI:** 10.1038/s41392-026-02670-z

**Published:** 2026-04-02

**Authors:** Flavia Lambertucci, Omar Motiño, Uxía Nogueira-Recalde, Yan Rong, Léa Montégut, María Pérez-Lanzón, Vincent Carbonnier, Sijing Li, Sylvère Durand, Fanny Aprahamian, Hui Chen, Yanbing Dong, Allan Sauvat, Silvia Mingoia, Sylvie Lachkar, Ester Saavedra, Jonathan Pol, Federico Pietrocola, Maria Chiara Maiuri, Estela Rocha-Oliveira, Roberto Roncon-Albuquerque, Francisco Vasques-Nóvoa, Roberto Lozano-Rodríguez, José Avendaño-Ortiz, Eduardo López-Collazo, Mahmoud Abdellatif, Isabelle Martins, Guido Kroemer

**Affiliations:** 1 Université Paris Cité, Sorbonne Université, Inserm, Centre de Recherche des Cordeliers, Paris, France; 2 Centre de Recherche des Cordeliers, Equipe labellisée par la Ligue contre le cancer, Institut Universitaire de France, Paris, France; 3 Université Paris-Saclay, INSERM US23/CNRS UAR 3655, Metabolomics and Cell Biology Platforms, Institut Gustave Roussy, Paris, France; 4 Unidad de Excelencia, Instituto de Biomedicina y Genética Molecular (IBGM), Consejo Superior de Investigaciones Científicas (CSIC)-Universidad de Valladolid, Valladolid, Spain; 5 Unidad de Biología del Cartílago, Grupo de Investigación en Reumatología (GIR), Instituto de Investigación Biomédica de A Coruña (INIBIC), Complejo Hospitalario Universitario de A Coruña (CHUAC), Sergas, Universidad de A Coruña (UDC), A Coruña, Spain; 6 Onco-Pheno-Screen Platform, Centre de Recherche des Cordeliers, Paris, France; 7 Department of Pharmacological Sciences, University of Piemonte Orientale, Novara, Italy; 8 Departamento de Bioquímica y Biología Molecular, Fisiología, Genética e Inmunología, Instituto Universitario de Investigaciones Biomédicas y Sanitarias (IUIBS), Universidad de Las Palmas de Gran Canaria, Las Palmas de Gran Canaria, Spain; 9 Department of Cell and Molecular Biology, Karolinska Institute, Stockholm, Sweden; 10 Department of Molecular Medicine and Medical Biotechnologies, University of Napoli Federico II, Naples, Italy; 11 RISE-Health, Departamento de Cirurgia e Fisiologia, Faculdade de Medicina, Universidade do Porto, Porto, Portugal; 12 Centro Hospitalar Universitário São João, Porto, Portugal; 13 The Innate Immune Response Group, Instituto de Investigación Hospital Universitario La Paz (IdiPAZ), La Paz University Hospital, Madrid, Spain; 14 Tumour Immunology Laboratory, Instituto de Investigación Hospital Universitario La Paz (IdiPAZ), La Paz University Hospital, Madrid, Spain; 15 Biobank Platform, Instituto de Investigación Hospital Universitario La Paz (IdiPAZ), La Paz University Hospital, Madrid, Spain; 16 Centre for Biomedical Research Network, Centro de Investigación Biomédica en Red (CIBER) of Respiratory Diseases (CIBERES), Madrid, Spain; 17 Biomedical Department, UNIE Universidad, Madrid, Spain; 18 BioTechMed-Graz, Graz, Austria; 19 Division of Cardiology, Medical University of Graz, Graz, Austria; 20 Institut du Cancer Paris CARPEM, Department of Biology, Hôpital Européen Georges Pompidou, AP-HP, Paris, France

**Keywords:** Translational research, Inflammation, Prognostic markers, Preclinical research

## Abstract

Sepsis remains a major clinical challenge, with high mortality and long-term disability despite current interventions. Here, we identify the tissue hormone acyl-CoA-binding protein (ACBP), also known as diazepam-binding inhibitor (DBI), as a biomarker and driver of poor outcome in sepsis. ACBP/DBI was elevated in the plasma of septic patients and associated with organ dysfunction and increased mortality. In murine models of endotoxemia, *Escherichia coli* infection, and polymicrobial sepsis, genetic deletion or antibody-mediated neutralization of ACBP/DBI conferred robust protection by dampening cytokine storm and preserving organ function. Across these three models, neutralization of ACBP/DBI with monoclonal antibodies restored thermoregulation and reduced mortality. Mechanistically, ACBP/DBI inhibition enhanced resilience to lipopolysaccharide-induced sterile inflammation and improved bacterial clearance by macrophages and granulocytes in vivo and in vitro. These effects were observed in monomicrobial infection models and confirmed by high-dimensional immunophenotyping in a polymicrobial sepsis model. Notably, ACBP/DBI inhibition could be favorably combined with glucocorticoids, enhancing survival and reversing histopathological, transcriptional or metabolic signatures of septic shock across heart, kidney, liver, lung, spleen and plasma. These findings position ACBP/DBI as a mechanistic amplifier of sepsis pathophysiology and propose its neutralization, alone or in combination with corticosteroids, as a promising therapeutic strategy to interrupt the fatal trajectory of septic shock.

## Introduction

Sepsis, with septic shock representing its most severe clinical presentation, remains a major challenge in critical care.^1–3^ Despite substantial progress in antimicrobial strategies, hemodynamic stabilization, and organ support, mortality from septic shock remains alarmingly high, ranging from 25 to 50% in contemporary cohorts.^4,5^ Moreover, survivors frequently experience long-term disability, cognitive impairment and reduced quality of life.^6,7^ Thus, novel mechanistic insights and targeted interventions are urgently needed to decipher and interrupt this maladaptive immunometabolic cascade.

Acyl-CoA-binding protein (ACBP), also known as diazepam-binding inhibitor (DBI) or endozepine, is a phylogenetically conserved ~10 kDa protein originally implicated in intracellular lipid trafficking.^8,9^ Beyond its intracellular role, ACBP/DBI is secreted in a noncanonical, autophagy-dependent manner and, in turn, functions as an extracellular signaling molecule with potent pro‑inflammatory and metabolic effects.^10,11^ ACBP/DBI is widely expressed in multiple organs and cell types (in particular epithelial and myeloid cells), and its expression is stimulated by various stress-associated transcription factors, including the glucocorticoid receptor.^12,13^ Hence, ACBP/DBI can be considered a tissue stress hormone. Elevated plasma levels of ACBP/DBI have been observed in conditions associated with systemic inflammation, including bacterial infections and viral diseases such as COVID‑19, associating with disease severity.^14,15^ Mechanistically, ACBP/DBI inhibits autophagy and activates immune cells to stimulate cytokine release through binding to gamma-aminobutyric acid type A (GABA_A_) receptors.^11,16,17^ As such, ACBP/DBI amplifies inflammatory cascades and exacerbates tissue injury, supporting its candidacy as a pathological cofactor in sepsis.^11,18,19^

Evidence from clinical trials supporting the use of low-to-moderate doses of glucocorticoids in septic shock highlights the complexity of their therapeutic use.^20^ On one hand, glucocorticoids are potent anti-inflammatory agents that can compensate for relative adrenal insufficiency and dampen the excessive cytokine release driving organ dysfunction in the early hyperinflammatory phase of sepsis, though with limited therapeutic effects.^20,21^ On the other hand, their metabolic and immunomodulatory side effects are well-documented in the context of both chronic^22,23^ and short-term exposure.^24^ Recent findings in patients with Cushing syndrome and experimental models have implicated glucocorticoids in the upregulation of ACBP/DBI, linking steroid therapy to a rise in this pro-inflammatory mediator.^13,25^ This duality poses a therapeutic dilemma: while glucocorticoids may confer benefit in patients with refractory septic shock, their efficacy may be partially offset by the induction of factors such as ACBP/DBI that undermine immune homeostasis and metabolic balance.

In light of these considerations, the present study addresses a threefold research question: First, is ACBP/DBI elevated in human sepsis? Second, does it play a causal role in the pathogenesis of sepsis in murine models? And if so, third, can its neutralization be favorably combined with corticotherapy? To address these knowledge gaps, we combined clinical observational studies and preclinical experimentation, demonstrating that ACBP/DBI levels are markedly increased in the serum of patients with sepsis, while its neutralization in mice confers significant protection against mortality and systemic inflammation in multiple relevant models, including lipopolysaccharide (LPS)-induced endotoxemia, monomicrobial bacterial infection with *Escherichia coli*, and polymicrobial sepsis induced by cecal ligation and puncture (CLP). Importantly, these beneficial effects are preserved, if not potentiated, when ACBP/DBI blockade is combined with glucocorticoid therapy, offering a potential synergistic strategy to counteract both hyperinflammation and its metabolic side effects. Altogether, these findings support the hypothesis that ACBP/DBI represents a novel therapeutic target in sepsis.

## Results

### Circulating ACBP/DBI is increased in human sepsis and septic shock and is associated with worse prognosis

**Fig. 1:**
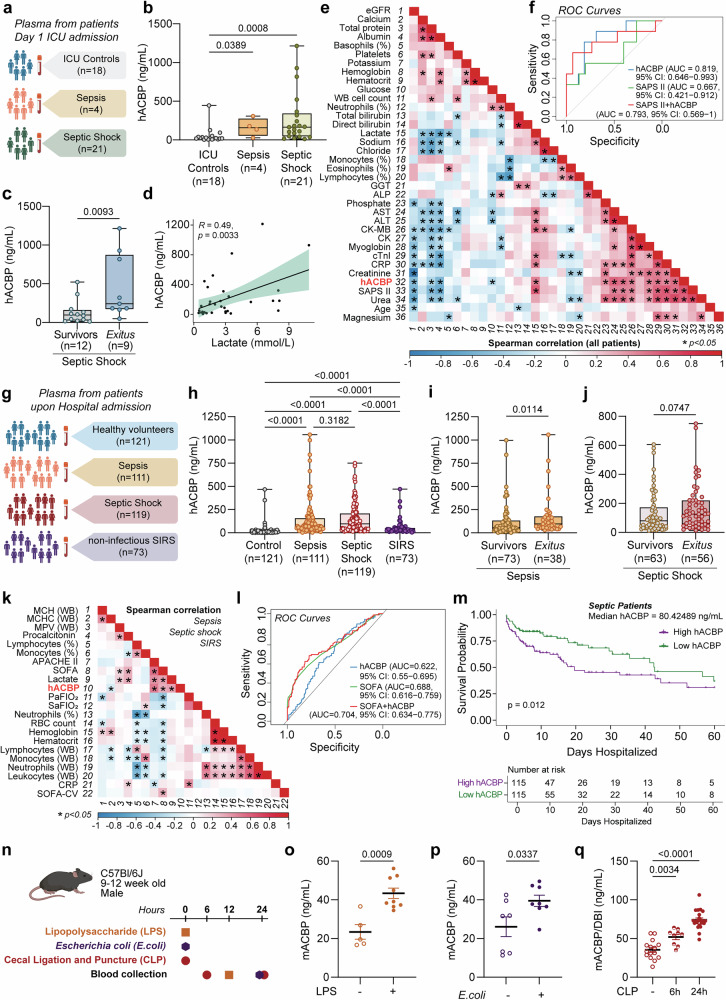
ACBP/DBI levels are elevated in human sepsis. **a** Plasma from patients with sepsis or septic shock was collected on day 1 of intensive care unit (ICU) admission. Individuals recovering from surgery in the ICU were used as controls. **b** Plasma human ACBP (hACBP) levels were measured by ELISA: ICU Controls (*n* = 18), Sepsis patients (*n* = 4), and septic shock patients (*n* = 21). **c** hACBP levels in plasma from survivors of septic shock (*n* = 12) and non-survivors (*Exitus*) (*n* = 9). **d** Plasma hACBP levels positively correlate with lactate, a prognostic marker in sepsis. **e** Hierarchically clustered correlation matrix highlighting different clinical parameters positively correlating with hACBP across all patients. **f** Receiver-operating characteristic (ROC) curves describing the predictive performance of plasma hACBP, SAPS II, and their combination in identifying which septic patients (n = 25) died following diagnosis. **g** Plasma from patients was collected upon hospital admission: Controls (healthy volunteers, *n* = 121), and patients with Sepsis (*n* = 111), Septic shock (*n* = 119), or non-infectious systemic inflammatory response syndrome (SIRS, *n* = 73). **h** Plasma hACBP levels were measured by ELISA. **i** hACBP levels in plasma from survivors (*n* = 73) and non-survivors (*n* = 38) with sepsis; or **j** from survivors (*n* = 63) and non-survivors (*n* = 56) with septic shock. **k** Hierarchically clustered correlation matrix showing different clinical parameters positively correlating with hACBP within patients with sepsis, septic shock, or SIRS. **l** Receiver operating characteristic (ROC) curves showing the predictive performance of plasma hACBP, SOFA score, and their combination in identifying mortality in septic patients from a second cohort (*n* = 230). **m** Kaplan–Meier survival curves stratified by median hACBP levels (cut-off: 80.42 ng/mL) in all septic patients (*n* = 230) showing worse prognosis in the “High hACBP” group (*p* = 0.012). **n** C57Bl/6J mice (9–12 weeks old) were injected with lipopolysaccharide (LPS, 20 mg/kg B.W. i.p.) or *Escherichia coli* (2.5 × 10⁶ CFU/mouse i.p.) and subjected to the cecal ligation and puncture (CLP) procedure (*n* = 5–20 mice per group). Murine ACBP (mACBP) was measured in plasma from **o** LPS-treated mice, **p** *E. coli*-injected mice, and **q** CLP-subjected mice at the indicated time points. Data in (**b**, **c**, **h**, **i**, **j**) are presented as box-and-whisker plots showing the median, interquartile range, and whiskers extending from minimum to maximum values. Individual data points are overlaid. Comparisons between groups were performed using Kruskal–Wallis or Wilcoxon signed-rank tests for plasma hACBP levels. Spearman correlation coefficients and *p* values were used for heatmaps and individual correlations. AUC confidence intervals in ROC curves were calculated using DeLong’s method. Kaplan–Meier survival analyses were stratified by median hACBP values, and significance assessed by the log-rank test. For in vivo mouse experiments, data are displayed as means ± SEM, and two-tailed unpaired Student’s t-tests were used. SIRS systemic inflammatory response syndrome, eGFR estimated glomerular filtration rate, WB whole blood, GGT γ-glutamyltransferase, ALP alkaline phosphatase, AST aspartate transaminase, ALT alanine transaminase, CK-MB creatine kinase-MB, CK creatine kinase, cTnI cardiac troponin I, CRP C-reactive protein, SAPS II Simplified Acute Physiology Score II, MCH mean corpuscular hemoglobin, MCHC mean corpuscular hemoglobin concentration, MPV mean platelet volume, RBC red blood cells, APACHE II Acute Physiology and Chronic Health Evaluation II, SOFA Sequential Organ Failure Assessment, SOFA-CV SOFA cardiovascular subscore

In a discovery sepsis cohort (*n* = 43), we found that ACBP/DBI plasma concentrations were higher in patients with septic shock than in the intensive care unit (ICU) control group, consisting of post-operative patients with aseptic inflammation (Fig. 1a, b). Importantly, ACBP/DBI levels were higher in those patients who died from septic shock than in survivors (Fig. 1c) and correlated with circulating lactate, a marker of hypoperfusion, stress-induced metabolic changes, and poor prognosis (Fig. 1d). In addition, ACBP/DBI plasma levels correlated with blood markers of renal, hepatic, and cardiac damage, namely, a decrease in estimated glomerular filtration rate (eGFR), an increase in the transaminases alanine aminotransferase (ALT), and aspartate aminotransferase (AST), and an elevation in cardiac troponin I levels, respectively (Fig. 1e, Supplementary Fig. S1a–h) and accurately predicted patient survival as efficiently as SAPS II (Simplified Acute Physiology Score II), a comprehensive scoring system used for estimating mortality risk in critically ill patients (Fig. 1f, Supplementary Table S1).

These results were confirmed in a much larger validation cohort^26^ (*n* = 424, Fig. 1g), showing that ACBP/DBI plasma levels were elevated in patients with sepsis and even more so, at least numerically, in those with septic shock compared to healthy controls or patients with non-infectious systemic inflammatory response syndrome (SIRS) (Fig. 1h). More convincingly, ACBP/DBI levels were higher in patients who died from sepsis, and a similar trend toward elevation was observed in patients who died from septic shock compared to survivors (Fig. 1i, j). Plasma concentrations of ACBP/DBI strongly correlated with key prognostic clinical parameters, including lactate levels, APACHE II (Acute Physiology and Chronic Health Evaluation II) score and SOFA (Sequential Organ Failure Assessment) score (Fig. 1k, Supplementary Fig. S1i–k). ACBP/DBI also yielded a superior predictive power for distinguishing sepsis survivors from non-survivors (reflected by the area under the receiver operating characteristic [ROC] curve [AUC]) than two commonly used prognostic parameters in sepsis as C-reactive protein (CRP) and procalcitonin (PCT) (Supplementary Fig. S1l, m). Indeed, its AUC was comparable to that of the SOFA and APACHE II scores (Fig. 1l, Supplementary Fig. S1n). Kaplan–Meier survival curves stratified by median ACBP/DBI levels in all septic patients indicated a worse prognosis for those with higher levels (Fig. 1m). While ACBP/DBI levels in sepsis remained positively correlated with both APACHE II and SOFA scores, this association was diminished in septic shock, where only the correlation with APACHE II persisted (Supplementary Fig. S1o–r, Supplementary Table S2).

Similar to patients, ACBP/DBI plasma levels were increased across sepsis models in mice, induced by three alternative methods, namely intraperitoneal (i.p.) LPS injection, infection with *E. coli*, or CLP (Fig. 1n–q). Taken together, these results clearly show that microbes and their products elicit a surge in circulating ACBP/DBI concentrations across species.

### ACBP/DBI neutralization protects against LPS-induced septic shock

**Fig. 2:**
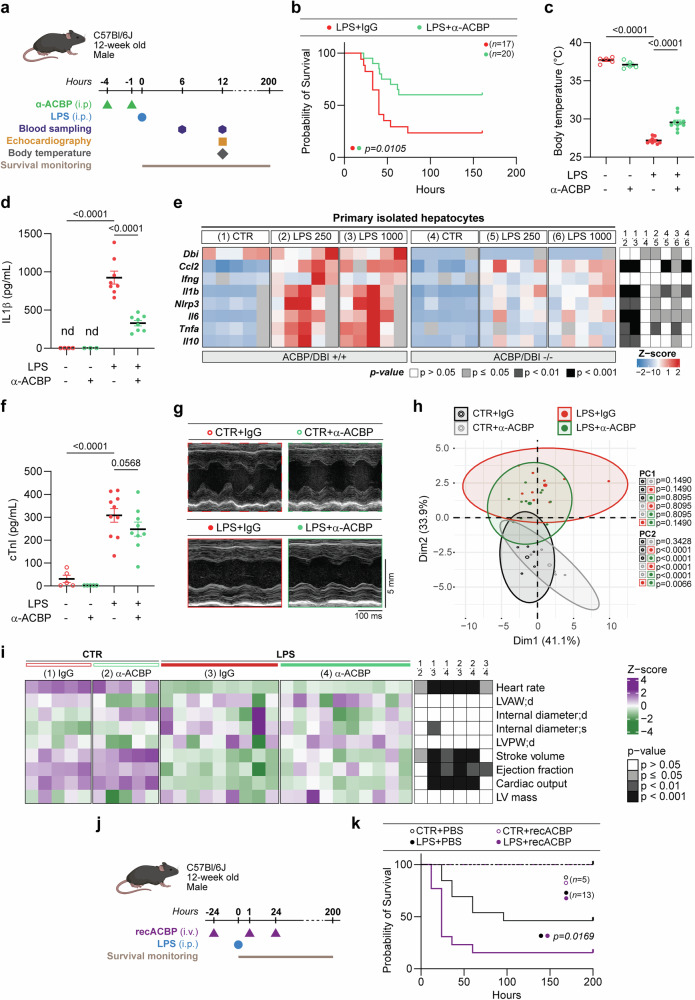
ACBP/DBI neutralization improves survival and mitigates cardiovascular dysfunction in a model of LPS-induced endotoxemia. **a** Twelve-week-old C57BL/6J mice were treated with either a monoclonal antibody against ACBP/DBI (α-ACBP) or an IgG2a isotype control (2.5 mg/kg body weight, i.p.), administered twice prior to LPS challenge. **b** Survival was monitored following LPS challenge (10 mg/kg body weight, i.p.) in mice (*n* = 17–20 per group). Statistical significance was assessed using the log-rank test. **c** Body temperature was measured 12 h post-LPS injection. **d** Plasma levels of interleukin-1β (IL-1β) were quantified 6 h post-LPS injection. **e** Primary hepatocytes isolated from inducible whole-body *Acbp/Dbi* knockout (*Acbp/Dbi*^−/−^) and control mice were treated in vitro with LPS (250 or 1000 ng/mL), and inflammatory gene expression was assessed by RT-qPCR at 6 h post-LPS. **f** Plasma cardiac troponin I (cTnI) levels were measured 12 h post-LPS (20 mg/kg body weight) in mice treated with or without α-ACBP. **g** Representative M-mode echocardiographic tracings of the left ventricle (LV) were obtained. **h** Principal component analysis (PCA) of echocardiographic parameters. **i** Heatmap of individual echocardiographic measurements. **j** Twelve-week-old C57BL/6J mice were treated with either the recombinant protein ACBP/DBI (recACBP, 8 mg/kg body weight, i.v.) or PBS (i.v.), administered once prior to LPS challenge, and twice post-LPS. **k** Survival was monitored following LPS challenge (10 mg/kg body weight, i.p.) in mice (*n* = 5–13 per group). Statistical significance was assessed using the log-rank test. All results are presented as mean ± SEM (*n* = 5–10 mice per group). Group comparisons were performed using one-way ANOVA followed by pairwise comparisons. For heatmap comparisons, *p* values were adjusted using false discovery rate (FDR) correction. Multivariate ANOVA followed by pairwise t-tests with FDR correction was used for group comparisons in PCA (PC1–PC2). LVAW;d LV anterior wall thickness in diastole, Internal diameter;d LV internal diameter in diastole, Internal diameter;s LV internal diameter in systole, LVPW;d LV posterior wall thickness in diastole, LV Mass estimated LV muscle mass

To determine whether ACBP/DBI plays a pathogenic role in sepsis, we used C57BL/6 J mice that were first injected i.p. with an anti-ACBP/DBI monoclonal antibody (mAb) or an isotype-matched IgG2a antibody (control), before receiving an i.p. injection of LPS, followed by blood sampling, echocardiographic assessment and survival monitoring (Fig. 2a). ACBP/DBI neutralization with an established non-toxic monoclonal antibody^10,11^ resulted in a significant reduction in LPS-induced mortality (Fig. 2b), coupled with an inhibition of LPS-induced hypothermia (Fig. 2c) and a reduced elevation of interleukin-1β (IL-1β) plasma levels (Fig. 2d). Similarly, isolated primary hepatocytes from *Dbi* knockout mice showed reduced *Il1b* mRNA levels upon in vitro LPS treatment, along with suppression of mRNAs encoding additional inflammatory cytokines including the chemokine *Ccl2*, the inflammasome component *Nlrp3*, interleukins *Il6* and *Il10*, as well as tumor necrosis factor-α (*Tnfa*) (Fig. 2e, Supplementary Fig. S2a–h). Concomitantly, LPS-elicited cardiac damage, measured by circulating cardiac Troponin I (cTnI), tended to be reduced upon ACBP/DBI neutralization (Fig. 2f). Importantly, non-invasive cardiac assessment by echocardiography (Fig. 2g) demonstrated a clear decline in cardiac performance following LPS administration, which was partially prevented by anti-ACBP/DBI mAb (Fig. 2h), as indicated by improvements in heart rate and left ventricular ejection fraction (Fig. 2i, Supplementary Table S3).

In summary, ACBP/DBI neutralization attenuates the LPS-induced cytokine storm, improves thermoregulation, and protects against cardiac dysfunction and injury. In contrast, administering recombinant ACBP/DBI protein intravenously (i.v.)—despite being non-toxic on its own—heightened the lethality of LPS in mice (Fig. 2j, k), suggesting that ACBP/DBI can act as a driver of septic shock pathogenicity.

### ACBP/DBI neutralization increases resistance to *E. coli* infection

**Fig. 3:**
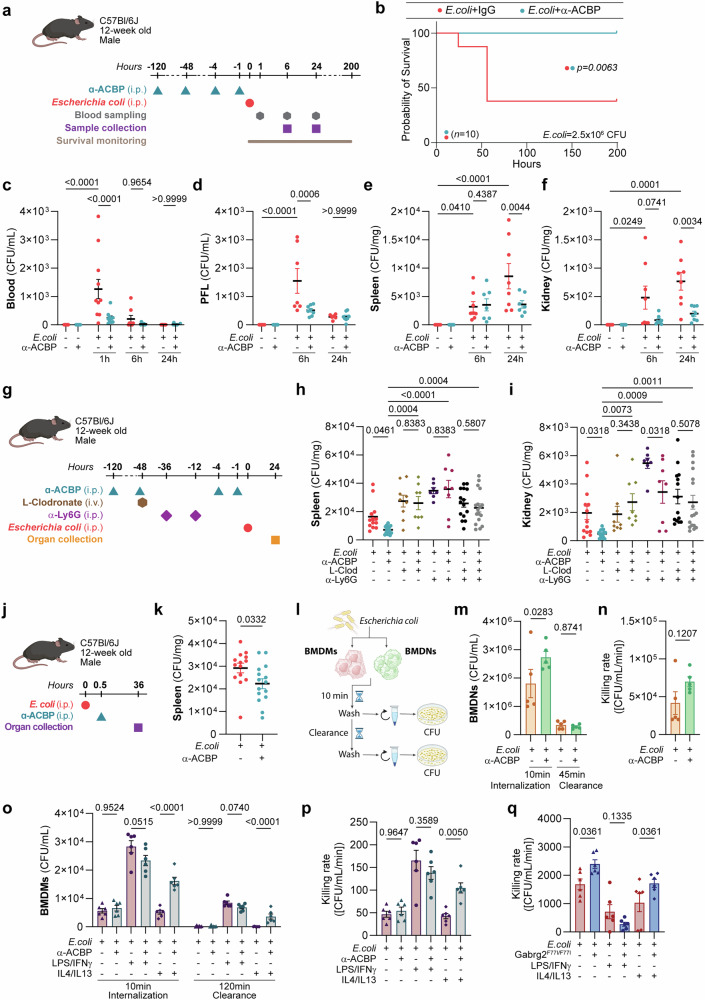
ACBP/DBI blockade increases resistance to *E. coli* infection. **a** Twelve-week-old male C57BL/6J mice were treated with monoclonal antibody against ACBP/DBI (α-ACBP; 5 mg/kg, i.p.) twice during the week prior to *E. coli* challenge, followed by two additional doses (2.5 mg/kg, i.p.) at 4 h and 1 h before infection. **b** Survival was monitored in mice (*n* = 10 per group) challenged intraperitoneally with 2.5 × 10⁶ CFU/mouse of *E. coli*. Statistical significance was assessed using the log-rank test. **c** Blood was collected at 1 h, 6 h, and 24 h post-infection (1 × 10⁶ CFU/mouse), plated on agar, and incubated overnight (O/N) at 37 °C for CFU counting (*n* = 4–8 mice per group). At 6 h and 24 h post-infection, **d** PLF, **e** spleens, and **f** kidneys were collected and processed for CFU quantification. **g** Mice were pre-treated with α-ACBP as in (**a**) and additionally injected with clodronate liposomes (100 µL/10 g body weight, i.v.) or control liposomes 48 h before infection, and anti-mouse Ly6G (clone 1A8, 50 µg/mouse) or isotype control IgG2a 36 h and 12 h prior to *E. coli* injection. Two doses of α-ACBP (2.5 mg/kg) were also administered prior to infection (*n* = 7–18 mice per group). At 24 h post-infection, **h** spleens and **i** kidneys were collected for CFU analysis. **j** Twelve-week-old male C57BL/6J mice (*n* = 15 mice per group) were challenged with *E. coli*, and 30 min after treated with the monoclonal antibody against ACBP/DBI (α-ACBP; 10 mg/kg, i.p.). **k** At 36 h post-infection, spleens were collected and processed for CFU quantification. **l** Bone marrow-derived neutrophils (BMDNs) or bone marrow-derived macrophages (BMDMs) were isolated from male C57BL/6J mice and treated with α-ACBP (5 µg/mL) or IgG2a control for 16 h. **m** A bacterial killing assay was performed with BMDNs (≥80% Ly6G⁺), and *E. coli* (MOI = 10); CFU/mL were quantified the following day (*n* = 5 mice per group). **n** Killing efficiency of BMDNs was calculated after 45 min of bacterial clearance. **o** BMDMs were differentiated over 7 days (≥70–80% F4/80⁺), then stimulated with LPS (100 ng/mL) + IFNγ (25 ng/mL) or IL-4 (25 ng/mL) + IL-13 (25 ng/mL) for 24 h, and treated with α-ACBP or IgG2a control for 16 h (*n* = 6 mice per group). Killing assays were performed with *E. coli* (MOI = 10), and CFU/mL were determined after overnight incubation. **p** Bacterial killing by BMDMs was quantified after 120 min and expressed as absolute values of [CFU/mL/min]. **q** BMDMs from male C57BL/6J and Gabrg2^F77I/F77I^ mice were differentiated over 7 days (≥70–80% F4/80⁺), then stimulated with LPS (100 ng/mL) + IFNγ (25 ng/mL) or IL-4 (25 ng/mL) + IL-13 (25 ng/mL) for 24 h (*n* = 6 mice per group). Killing assays were performed with *E. coli* (MOI = 10), and bacterial killing by BMDMs was quantified after 120 min and expressed as absolute values of CFU/mL/min. Data are presented as means ± SEM. Statistical comparisons were performed using one-way or two-way ANOVA with estimation of marginal means for pairwise comparisons, or Student’s t-test where applicable. **l** was generated with “BioRender.com.” IFNγ interferon-γ, IL-4 interleukin-4, IL-13 interleukin-13, MOI multiplicity of infection, CFU colony-forming units, PLF peritoneal lavage fluid

In the next series of experiments, we pre-treated mice with anti-ACBP/DBI mAb (or an isotype-matched control antibody) and then inoculated them i.p. with live *E. coli* (strain ATCC 25922), followed by blood sampling, organ collection or survival monitoring (Fig. 3a). ACBP/DBI neutralization strongly reduced the lethality of *E. coli* injections (Fig. 3b, Supplementary Fig. S3a) as it improved the clearance of live bacteria able to produce colonies (colony-forming units, CFU) in blood, peritoneal fluid lavage (PFL), spleen, and kidney (Fig. 3c–f, Supplementary Fig. S3b) and reduced plasma levels of the hepatic transaminase AST (Supplementary Fig. S3c, d). Simultaneous depletion of macrophages by liposome-encapsulated clodronate or elimination of neutrophils by anti-Ly6G antibody (Supplementary Fig. S3e–g) compromised the clearance-accelerating effect of ACBP/DBI neutralization (Fig. 3g–i), suggesting that these myeloid cells (which do express ACBP/DBI)^12^ mediate the bacterial clearance increased by ACBP/DBI blockade. Furthermore, ACBP/DBI neutralization following *E. coli* challenge reduced splenic bacterial burden in a therapeutic setting (Fig. 3j, k).

Accordingly, in vitro, anti-ACBP/DBI mAb enhanced the internalization of *E. coli* by freshly isolated bone marrow-derived neutrophils (BMDNs) (Fig. 3l, m; Supplementary Fig. S3h, i), as well as the capacity of such neutrophils to kill *E. coli* (Fig. 3n). Short-term exposure (10 min) to *E. coli* induced ACBP release, as indicated by a rapid reduction in intracellular ACBP/DBI immunostaining, and anti-ACBP/DBI mAb treatment reduced ELISA-detectable ACBP/DBI in the supernatant of BMDMs (Supplementary Fig. S3j, k). Similarly, anti-ACBP/DBI mAb stimulated the phagocytosis and killing of *E. coli* by alternatively activated bone marrow-derived macrophages (BMDMs cultured in IL-4/IL-13) but not by classically activated BMDMs (cultured with LPS/IFNγ) or unstimulated BMDMs, as determined by plating macrophage lysates on agar plates (Fig. 3o, p; Supplementary Fig. S3l, m). Again, short-term exposure to *E. coli* reduced the intracellular abundance of ACBP/DBI in BMDMs, and decreased ACBP/DBI levels were detectable in the supernatants upon anti-ACBP/DBI mAb treatment (Supplementary Fig. S3n, o). Of note, as compared to isogenic WT controls, primary and alternatively activated BMDMs from *Gabrg2*^F77I/F77I^ mice bearing the F77I loss-of-function mutation in the GABA_A_R subunit Gabrg2 (which disrupts ACBP/DBI binding)^17^ exhibited a constitutive increase in phagocytosis and killing activity (Fig. 3q; Supplementary Fig. S3p). Similarly, fluorescence confocal microscopy detected improved phagocytosis by alternatively activated BMDMs of *E. coli* engineered to express green fluorescent protein in the presence of anti-ACBP/DBI mAb (Supplementary Fig. S4a–c). Additionally, neutralization of ACBP/DBI in BMDMs induced phenotypic alterations as assessed by flow cytometry (Supplementary Fig. S4d–g).

Altogether, these findings demonstrate that ACBP/DBI inhibition improves the clearance of bacteria by myeloid cells and increases the resistance of mice to otherwise lethal doses of *E. coli*.

### ACBP/DBI neutralization increases resistance to polymicrobial peritonitis

**Fig. 4:**
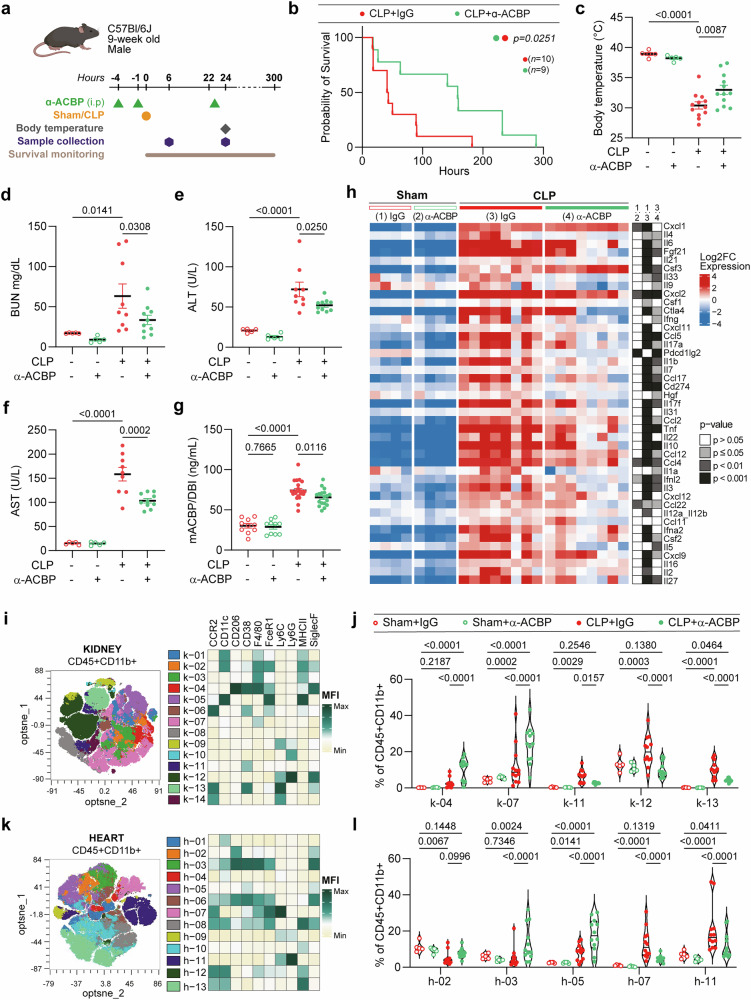
Neutralization of ACBP/DBI reduces sepsis-associated damage induced by CLP in mice. **a** Nine-week-old male C57BL/6J mice were treated with monoclonal antibody against ACBP/DBI (α-ACBP; 2.5 mg/kg, i.p.) or isotype control, followed 4 h later by high-grade CLP surgery. Additional doses of α-ACBP (5 mg/kg, i.p.) or IgG isotype were administered 90 min before sample collection at 24 h. **b** Survival was monitored in CLP-treated groups (*n* = 9–10 per group). The *p* value from the log-rank test is shown. **c** Body temperature was recorded at 24 h post-CLP. Plasma markers of organ injury and inflammation were measured at 24 h: **d** blood urea nitrogen (BUN), **e** alanine aminotransferase (ALT), **f** aspartate transaminase (AST), and **g** mACBP/DBI. **h** Plasma levels of 43 cytokines were measured at 24 h post-CLP using a proximity extension assay. Cytokine expression was compared among control mice (Sham) or those submitted to CLP and treated with α-ACBP or isotype. Statistical comparisons were performed by two-way ANOVA with FDR correction for multiple comparisons on log2-normalized cytokine data. **i** Immune cell populations in the kidney were analyzed by flow cytometry 24 h post-CLP (*n* = 5–12 mice per group). Fourteen populations were defined within CD45⁺CD11b⁺ cells using manual gating, and median fluorescence intensity (MFI) of each marker is represented in a heatmap. **j** Relative abundance of selected kidney immune clusters that were significantly modulated by ACBP neutralization is shown as a percentage of CD45⁺CD11b⁺ cells, comparing septic and control mice. **k** Similarly, 13 immune populations were defined in the heart within CD45⁺CD11b⁺ cells, with the corresponding MFI heatmap shown (*n* = 5–12 mice per group). **l** Relative abundance of significant heart immune populations affected by ACBP neutralization is displayed as a percentage of CD45⁺CD11b⁺ cells. Data are presented as means ± SEM. Statistical comparisons were performed using one-way ANOVA with estimation of marginal means for pairwise comparisons. Immune population analyses used two-way ANOVA followed by pairwise comparisons. Compensation, scaling, and gating strategies were conducted using the Omiq.ai platform

CLP causes polymicrobial peritonitis and sepsis, which is considered a more clinically relevant and heterogeneous model of septic shock than the injection of purified LPS or monomicrobial *E. coli* infection.^27^ ACBP/DBI blockade mediated by the specific mAb injected before CLP delayed the lethal outcome of this procedure (Fig. 4a, b), blunted hypothermia (Fig. 4c), improved renal function (as indicated by reduced levels of blood urea nitrogen, BUN, Fig. 4d), reduced the concentration of circulating liver transaminases (Fig. 4e, f), partially reduced ELISA-detectable ACBP/DBI in the plasma (Fig. 4g) and also reduced the circulating concentrations of multiple inflammatory cytokines induced by CLP (Fig. 4h).

Given the effects of ACBP/DBI on myeloid cells, we characterized the myeloid immune infiltrate of kidneys, hearts, spleens, and livers by high-dimensional immunofluorescence cytometry, followed by dimensionality reduction visualization using an optimized version of t-SNE (t-distributed Stochastic Neighbor Embedding). This analysis revealed major shifts in myeloid immune subsets, with a CLP-associated increase in pro-inflammatory subpopulations that were attenuated by ACBP/DBI neutralization (e.g., the subsets labeled as k-11, k-12, k-13 in kidney; h-07, h-11 in heart; s-01, s-02, s-18 in spleen; and l-05 in liver),^28^ as well as the expansion of CD206-positive, anti-inflammatory subpopulations induced by ACBP/DBI inhibition (e.g., the subsets labeled as k-04 in kidney; h-02, h-03 in heart; and l-04 in liver, Fig. 4i–l, Supplementary Fig. S5a–i).^29^ These observations highlight a major role for Ly6G⁺ and F4/80⁺ cells in the heart and kidney, and specifically for Ly6G⁺ cells in the spleen and liver, the latter confirmed by histological analysis (Supplementary Fig. S5j–m).

**Fig. 5:**
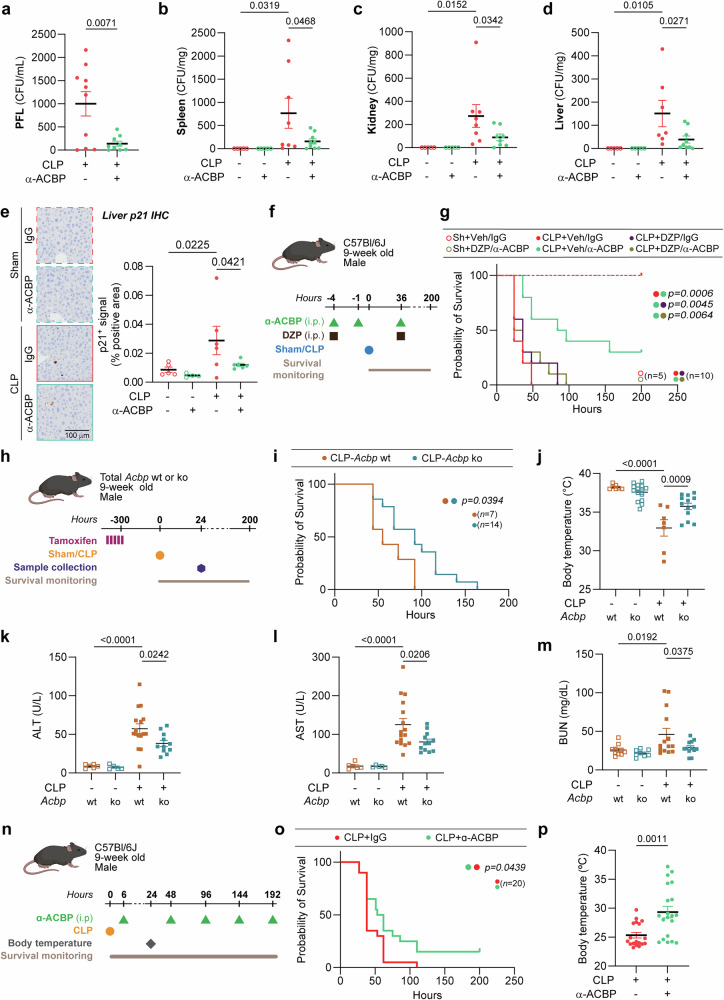
Genetic ablation or therapeutic neutralization of ACBP/DBI protects against CLP-induced damage and senescence. Bacterial clearance was improved by ACBP/DBI neutralization in **a** peritoneal fluid lavage (PFL) at 6 h post-CLP, and in **b** spleen, **c** kidney, and **d** liver 24 h post-CLP (*n* = 5–10 mice per group). **e** Representative immunohistochemistry (IHC) images showing p21 senescence marker in livers of Sham controls or CLP mice treated with α-ACBP, and quantification of p21⁺ IHC signal indicating increased p21 presence in CLP livers, which is reduced by α-ACBP treatment (*n* = 4–6 mice per group). **f** Nine-week-old male C57BL/6J mice were treated with α-ACBP (2.5 mg/kg, i.p.) twice in combination with one dose of diazepam (DZP, 4 mg/kg, i.p.) 4 h prior to sham or CLP surgery. **g** Survival was monitored over time (*n* = 5–10 per group). The *p* value of the log-rank test is indicated. **h** Nine-week-old inducible whole-body Acbp/Dbi knockout (Acbp/Dbi^−/−^) or control male mice were injected with tamoxifen for 5 consecutive days prior to sham or CLP surgery. **i** Survival was monitored post-surgery (*n* = 7–14 per group). Statistical significance was determined using the log-rank test. **j** Body temperature was measured at 24 h following CLP. Plasma levels of **k** ALT, **l** AST, and **m** BUN were assessed (*n* = 5–18 mice per group). **n** Nine-week-old male C57BL/6J mice were treated with monoclonal antibody against ACBP/DBI (α-ACBP; 10 mg/kg, i.p.) or isotype control, 6 h after high-grade CLP surgery and every 48 h. **o** Survival was monitored in CLP-treated groups (*n* = 20 per group). The *p* value from the log-rank test is shown. **p** Body temperature was recorded at 24 h post-CLP. Data are presented as means ± SEM. Statistical comparisons were performed using one-way ANOVA with estimation of marginal means for pairwise comparisons, or Student’s t-test, where applicable

Importantly, in the CLP model, neutralization of ACBP/DBI reduced the number of bacteria present in the peritoneal fluid and various internal organs, confirming enhanced microbial clearance (Fig. 5a–d). Bulk transcriptomics of the liver confirmed that multiple genes involved in inflammation were upregulated by CLP but downregulated by ACBP/DBI inhibition (Supplementary Fig. S6a, b). Intriguingly, CLP caused the hepatic upregulation of the pro-senescence gene *Cdkn1a* (which codes for the protein p21) that was blunted by ACBP/DBI inhibition (Supplementary Fig. S6c). Accordingly, CLP caused an anti-ACBP/DBI mAb-repressible increase in the frequency of hepatic p21-expressing cells detectable by immunohistochemistry (Fig. 5e). Bulk transcriptomics of the lung confirmed the capacity of ACBP/DBI inhibition to downregulate CLP-induced genes (Supplementary Fig. S6d). Accordingly, CLP-induced major histological alterations of the lung with increased infiltration by Ly6G^+^ granulocytes that were dampened by ACBP/DBI neutralization (Supplementary Fig. S6e–h).

Of note, the life-preserving effect of the anti-ACBP/DBI mAb was abolished upon administration of the benzodiazepine diazepam, which, like ACBP/DBI, acts on GABA_A_R (Fig. 5f, g), Moreover, the beneficial effects of the anti-ACBP/DBI mAb were mimicked by whole body knockout of the *Dbi* gene (Fig. 5h–m). Finally, the anti-ACBP/DBI mAb increased survival and maintained body temperature not only in a prophylactic setting but also when it was administered 6 h after CLP (Fig. 5n–p), i.e., at a time point when the peritoneum has already undergone invasion by gut microbes (Fig. 5a) and ACBP/DBI plasma levels are significantly increased (Fig. 1q).

Altogether, these findings support the idea that ACBP/DBI contributes to the CLP-triggered maladaptive and ultimately lethal inflammation affecting major organs, including kidney, heart, liver, spleen, and lung.

### Favorable interaction between ACBP/DBI neutralization and glucocorticoids

**Fig. 6:**
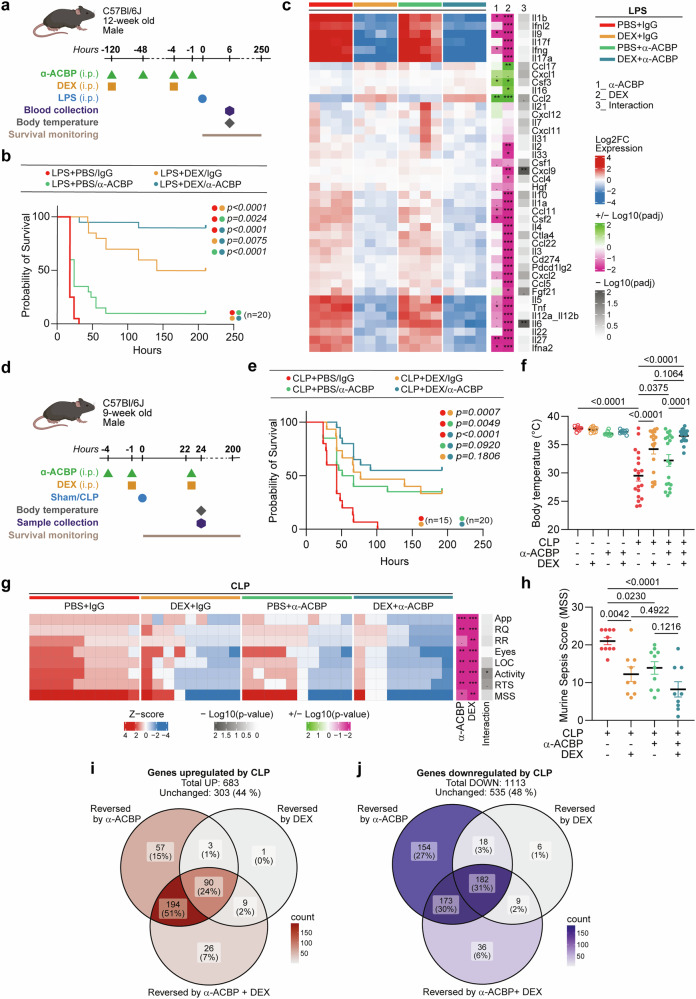
Neutralization of ACBP/DBI combined with dexamethasone enhances protection against LPS-induced challenge and CLP-induced sepsis in mice. **a** Twelve-week-old male C57BL/6J mice were treated with monoclonal antibody against ACBP/DBI (α-ACBP; 5 mg/kg, i.p.) twice, in combination with one dose of dexamethasone (DEX; 10 mg/kg, i.p.) during the week prior to LPS challenge. Additionally, two doses of α-ACBP (2.5 mg/kg) and one dose of DEX (10 mg/kg) were administered 4 h or 1 h before LPS injection. **b** Survival was monitored in LPS-challenged mice (*n* = 20 per group). The *p* value from the log-rank test is shown. **c** Plasma levels of 42 cytokines were measured at 6 h post-LPS challenge using a proximity extension assay. Cytokine expression was compared among groups treated with α-ACBP, DEX, both, or vehicle. Statistical comparisons were performed by two-way ANOVA with FDR correction for multiple comparisons on log2-normalized cytokine data. **d** Nine-week-old male C57BL/6J mice were treated with α-ACBP (2.5 mg/kg, i.p.) twice in combination with one dose of DEX (10 mg/kg, i.p.) 1 h prior to sham or CLP surgery. **e** Survival was monitored over time (n = 15–20 per group). The *p* value of the log-rank test is indicated. **f** Body temperature was recorded 24 h post-surgery. **g** Murine Sepsis Score (MSS) was assessed on a scale of 0 to 4 based on multiple clinical parameters—appearance (App), respiration quality (RQ), respiration rate (RR), eyes, level of consciousness (LOC), activity, and response to stimulus (RTS)—by two independent examiners. Scores were averaged per mouse and depicted as Z-scores in a heatmap analyzed by two-way ANOVA with FDR correction. **h** Individual MSS values are shown. **i** Venn diagram illustrating overlap among upregulated differentially expressed genes (DEGs) in spleens from CLP mice, categorizing genes reversed by α-ACBP alone, by DEX alone, or specifically by the combination treatment. **j** Venn diagram showing overlap of downregulated DEGs in the same groups. Data are presented as means ± SEM. Statistical comparisons were performed using one-way ANOVA (**h**) and two-way ANOVA (**f**) with estimation of marginal means for pairwise comparisons. il1b interleukin-1 beta, ifnl2 interferon lambda 2, il9 interleukin-9, il17f interleukin-17f, ifng interferon gamma, il17a interleukin-17a, ccl17 c-c motif chemokine ligand 17, cxcl1 c-x-c motif chemokine ligand 1, csf3 colony stimulating factor 3, il16 interleukin-16, ccl2 c-c motif chemokine ligand 2, il21 interleukin-21, cxcl12 c-x-c motif chemokine ligand 12, il7 interleukin-7, cxcl11 c-x-c motif chemokine ligand 11, il31 interleukin-31, il2 interleukin-2, il33 interleukin-33, csf1 colony stimulating factor 1, cxcl9 c-x-c motif chemokine ligand 9, ccl4 c-c motif chemokine ligand 4, hgf hepatocyte growth factor, il10 interleukin-10, il1a interleukin-1 alpha, ccl11 c-c motif chemokine ligand 11, csf2 colony stimulating factor 2, il-4 interleukin-4, ctla4 cytotoxic t-lymphocyte associated protein 4, ccl22 c-c motif chemokine ligand 22, il3 interleukin-3, cd274 cluster of differentiation 274 (pd-l1), pdcd1lg2 programmed cell death 1 ligand 2 (pd-l2), cxcl2 c-x-c motif chemokine ligand 2, ccl5 c-c motif chemokine ligand 5, fgf21 fibroblast growth factor 21, il5 interleukin-5, tnf tumor necrosis factor, il12a_il12b interleukin-12 subunits (p35 and p40), il-6 interleukin-6, il22 interleukin-22, il27 interleukin-27, ifna2 interferon alpha 2

To evaluate the combined effects of glucocorticoids and ACBP/DBI inhibition, we administered dexamethasone (DEX), a well-known and validated synthetic glucocorticoid receptor agonist, alongside anti-ACBP/DBI mAb treatment. DEX administration alone was more effective than ACBP/DBI neutralization in improving animal survival after i.p. LPS challenge. However, the combination of both treatments (anti-ACBP/DBI mAb plus DEX) was even more effective than the monotherapies, allowing 90% of the mice to survive (Fig. 6a, b). Accordingly, DEX was more efficient in reducing the LPS-elicited cytokine storm than anti-ACBP/DBI mAb, and the combination of both was particularly strong in downregulating critical cytokines such as Il-6 and Cxcl9 (Fig. 6c). Of note, the DEX-induced inhibition of the LPS-elicited cytokine storm, as well as its effect on restoring body temperature, remained intact in mice receiving the anti-ACBP/DBI mAb (Supplementary Fig. S7a–d), confirming that ACBP/DBI neutralization does not interfere with the anti-inflammatory effects of DEX.

In the CLP model, the combination of anti-ACBP/DBI mAb plus DEX yielded slightly more favorable, though statistically non-significant survival scores than the monotherapies (Fig. 6d, e). Only the combination of both anti-ACBP/DBI mAb and DEX was able to normalize the body temperature after CLP (Fig. 6f). Anti-ACBP/DBI mAb and DEX interacted favorably to enhance the activity score of the mice (Fig. 6g) and yielded a better clinical outcome (quantified as the murine sepsis score, MSS) than anti-ACBP/DBI mAb or DEX alone (Fig. 6h).

Transcriptomics analysis by RNA-sequencing (RNA-seq) demonstrated that splenic gene expression changes induced by CLP were attenuated toward the sham profile with a gradient of efficacy (DEX < anti-ACBP/DBI mAb < the combination of DEX plus anti-ACBP/DBI mAb) (Supplementary Fig. S7e). Among the CLP-upregulated genes (Fig. 6i) or downregulated genes (Fig. 6j), a large number was reversed by ACBP/DBI neutralization (up: 344; down: 527), and many of these genes were also reversed by DEX (the overlap with ACBP being up: 93; down: 200) and reversed by anti-ACBP/DBI mAb plus DEX as well (overlap with ACBP being up: 284; down: 355). In contrast, the number of CLP-regulated genes that were affected only by DEX and not by anti-ACBP/DBI was rather small (up: 1; down: 6). Notably, the capacity of DEX to reverse CLP-induced gene expression changes in the spleen (up: 103; down:215) remained intact in the context of ACBP/DBI neutralization (Fig. 6i, j). Mass spectrometric metabolomics performed on the spleen revealed that the majority of the metabolites that were upregulated or downregulated by CLP were indistinguishably reversed to normal levels by anti-ACBP/DBI mAb, DEX, or the combination of both (65% of the metabolites that were upregulated and 80% of those that were downregulated) (Supplementary Fig. S7f–h). Very similar results were obtained from plasma metabolomics, leading to the conclusion that single agents and combinations convergently normalized 71% of the metabolites that were upregulated and 87% of those that were downregulated (Supplementary Fig. S8a–c).

The CLP-induced elevation of liver transaminases was similarly reduced by each of the treatments: anti-ACBP/DBI mAb, DEX, or the combination of both (Supplementary Fig. S8d, e). ACBP/DBI neutralization, alone or in combination with DEX, was more efficient in reducing histological signs of liver damage than DEX alone (Supplementary Fig. S8f, g). Again, the metabolic normalization achieved by the three treatments in the livers from CLP-treated mice occurred in a convergent fashion, leading to a large overlap of effects (Supplementary Fig. S8h, i).

**Fig. 7:**
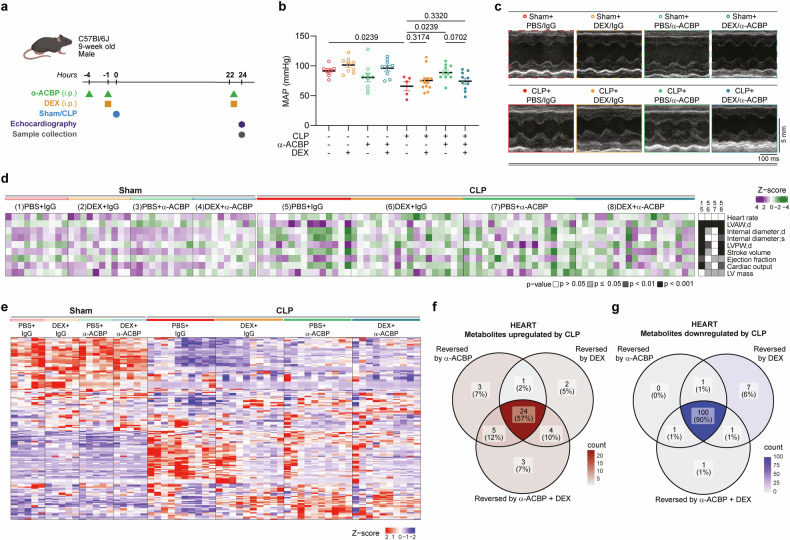
Co-administration of DEX and ACBP/DBI neutralization ameliorates sepsis-associated cardiomyopathy following CLP surgery. **a** Nine-week-old male C57BL/6J mice were treated with the monoclonal antibody against ACBP/DBI (α-ACBP; 2.5 mg/kg, i.p.) twice, in combination with one dose of DEX (10 mg/kg, i.p.) administered 1 h before sham or CLP surgery. **b** Mean arterial pressure (MAP) was measured 24 h post-surgery in sham and CLP mice treated with α-ACBP, DEX, or both. Data are presented as means ± SEM. Statistical comparisons were performed by two-way ANOVA followed by pairwise comparisons. **c** Representative echocardiographic M-mode images of LV function from the treatment groups. **d** Quantitative analysis of LV echocardiographic parameters. Group comparisons were assessed using one-way ANOVA followed by FDR correction for multiple comparisons. Adjusted *p* values are reported (*n* = 10–20 mice per group). **e** Heatmap clustered by Euclidean distance showing Z-score–normalized changes in cardiac metabolite concentrations in sham and CLP mice treated with α-ACBP, DEX, or the combination. Venn diagram showing overlap of **f** upregulated or **g** downregulated differentially expressed metabolites in CLP hearts and those reversed by α-ACBP, DEX, or their combination

We investigated cardiac phenotypes of mice after CLP in the context of anti-ACBP/DBI mAb, DEX, or combination treatments (Fig. 7a). Arterial hypotension was best antagonized by anti-ACBP/DBI mAb treatment alone (Fig. 7b). Quantitative echocardiography (Fig. 7c) revealed that, while some parameters were differentially improved by either anti-ACBP/DBI mAb or DEX alone, the combination yielded the best outcome, particularly regarding the normalization of stroke volume and cardiac output (Fig. 7d, Supplementary Table S4).^30^ Metabolomic analyses of the myocardium confirmed at the biochemical level a large overlap of normalization effects between anti-ACBP/DBI mAb, DEX, and their combination on metabolites that are up- or downregulated after CLP (Fig. 7e–g).

**Fig. 8:**
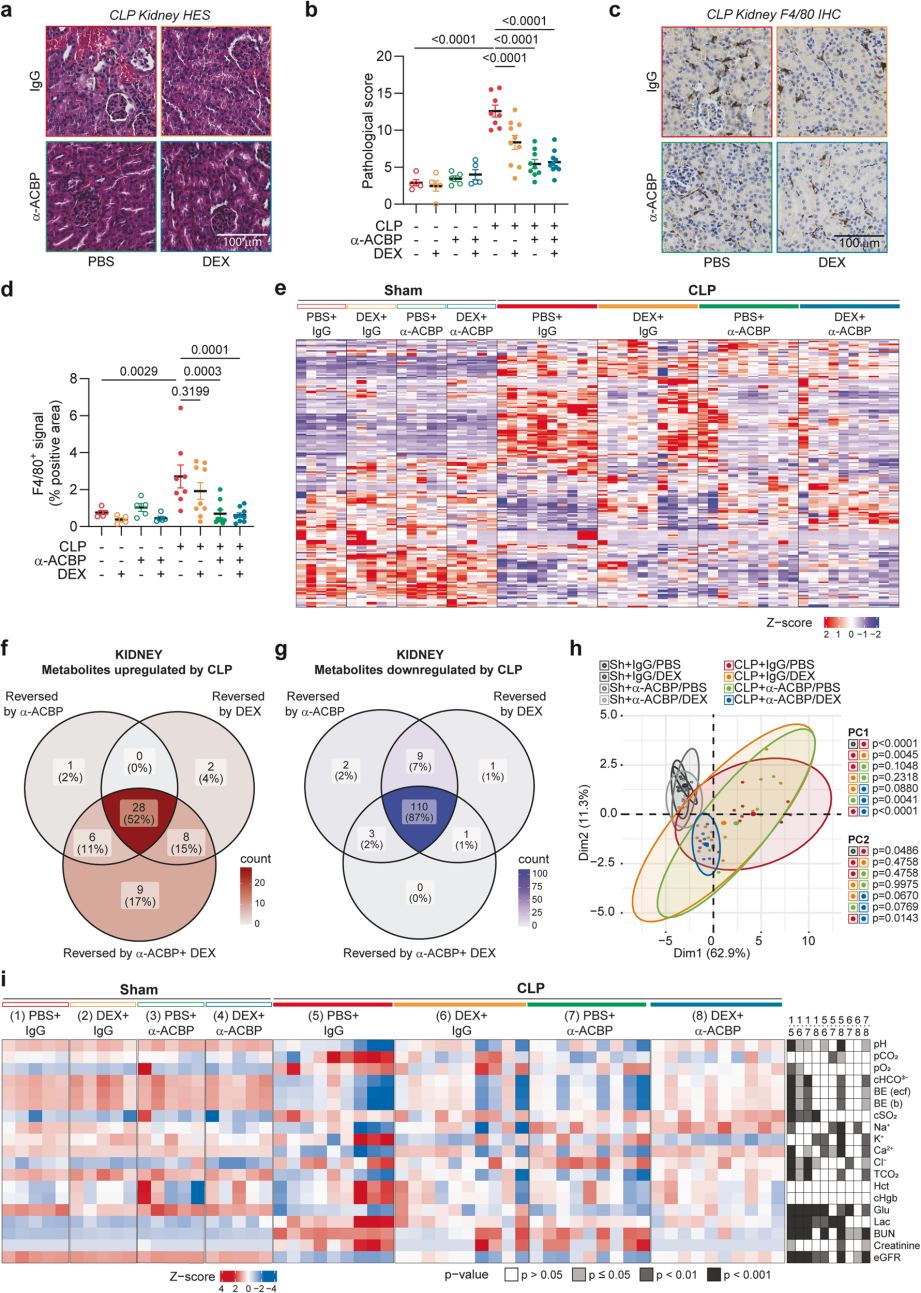
Co-administration of DEX and α-ACBP attenuates renal damage in CLP-induced sepsis. **a** Representative hematoxylin-eosin-safranin (HES) stained kidney sections showing renal damage in CLP mice. **b** Quantification of renal pathology scores (scale 0–4), based on tubular necrosis, brush border loss, tubular dilation, tubular cast formation, widening of the interstitium, degeneration, and inflammation. **c** Representative immunohistochemistry (IHC) images showing F4/80⁺ macrophage infiltration in kidneys of CLP mice treated with α-ACBP, DEX, or the combination. **d** Quantification of F4/80⁺ IHC signal indicating increased macrophage presence in CLP kidneys, which is reduced by α-ACBP alone and further attenuated by combined α-ACBP + DEX treatment. **e** Heatmap (Z-score) of renal metabolite concentrations clustered by Euclidean distance in sham or CLP mice treated with α-ACBP, DEX, or their combination. Venn diagram of **f** upregulated or **g** downregulated differentially expressed metabolites in CLP kidneys, showing subsets reversed by α-ACBP, DEX, or specifically by the combination. **h** PCA of kidney-related blood parameters at 24 h post-surgery in sham or CLP mice treated with α-ACBP, DEX, or both. **i** Heatmap of measured blood parameters (Z-score), including gases, electrolytes, and metabolites. Data in (**b**, **d**) are presented as mean ± SEM (*n* = 5–10 mice per group). Statistical significance was assessed using one-way or two-way ANOVA with false discovery rate (FDR) correction for multiple comparisons. PCA group differences were analyzed using multivariate ANOVA followed by pairwise t-tests. pCO₂ partial pressure of carbon dioxide, pO₂ partial pressure of oxygen, cHCO₃⁻ bicarbonate concentration, BE (ecf) base excess in extracellular fluid, BE (b) base excess in whole blood, cSO₂ calculated oxygen saturation, Na⁺ sodium, K⁺ potassium, Ca²⁺ calcium, Cl⁻ chloride, TCO₂ total carbon dioxide, Hct hematocrit, cHgb calculated hemoglobin, Glu glucose, Lac lactate, BUN blood urea nitrogen, eGFR estimated glomerular filtration rate

With respect to renal pathology, ACBP/DBI inhibition, alone or in combination with DEX, appeared more efficient in mitigating histological signs of organ damage (Fig. 8a, b; Supplementary Fig. S9a) and infiltration by F4/80^+^ macrophages (Fig. 8c, d; Supplementary Fig. S9b) than DEX alone. However, the biochemical effects of anti-ACBP/DBI mAb, DEX, or the combination of both on metabolites up- or downregulated by CLP largely converged (Fig. 8e–g). We also performed analyses of renal clearance and kidney effects on blood parameters to identify favorable combination effects with respect to acid-base balance, BUN, eGFR, and various electrolytes (K^+^, Ca^2+^, Cl^−^). Thus, the combination of anti-ACBP/DBI mAb and DEX yields superior normalization effects compared to each of the treatments alone, as indicated by principal component analysis (Fig. 8h) and statistical assessment of individual parameters (Fig. 8i; Supplementary Fig. S9c–u).

At the level of metabolomic shifts measured in at least 4 out of 5 anatomical components (heart, kidney, liver, plasma, spleen, Supplementary Figs. S10 and S11a, Supplementary Table S5), several metabolites that were elevated after CLP, such as the oxidative stress biomarker allantoin, the pro-oxidant malonic acid, the monoamine metabolite homovallinic acid, the pentose sugar xylose, the bacterial metabolites N-acetylputrescine and hydroxyphenyllactic acid, and the kynurenine metabolite 3-hydroxyanthranilic acid, were reduced by both anti-ACBP/DBI mAb and DEX, as well as by their combination, indicating shared effects of both monotherapies. However, one CLP-induced metabolite, arabinose, was only corrected by the combination of anti-ACBP/DBI mAb with DEX, but not by either monotherapy. Other CLP-induced metabolites were only reduced by anti-ACBP/DBI mAb, not by DEX, as this applies to N-acetylglutamic acid, 2-hydroxybutyric acid, 2-hydroxyethanesulfonic acid, 3-indoxylsulfuric acid, glucuronic/galacturonic acid, and, most prominently, the polyamine catabolism products N^1^-acetylspermidine, N,^1^N^12^-diacetylspermine, and N^8^-acetylspermidine. Of note, the magnitude of the metabolic correction induced by anti-ACBP/DBI mAb, DEX, or their combination was comparable in the four organs analyzed, supporting the idea that these effects are systemic rather than organ-specific (Supplementary Fig. S11b–d). At the hepatic level, we identified several mRNAs encoding metabolic enzymes whose expression was significantly altered by CLP and subsequently restored by treatment with the anti-ACBP/DBI mAb. These transcriptional changes aligned with the metabolic rewiring observed in the liver. Notably, *Smox*, which encodes the polyamine-catabolizing enzyme spermine oxidase, was significantly upregulated following CLP and corrected upon anti-ACBP/DBI mAb administration. This reversal is consistent with the observed reduction in polyamine-derived catabolites, including N¹-acetylspermidine, N¹,N¹²-diacetylspermine, and N⁸-acetylspermidine (Supplementary Fig. S12).

In sum, ACBP/DBI neutralization and corticosteroid therapy can be favorably combined in the treatment of septic shock induced by LPS or CLP in mice.

## Discussion

Septic shock remains a highly lethal clinical entity characterized by dysregulated host responses that culminate in multi-organ failure and death. Our study identifies ACBP/DBI as a novel and actionable mediator of persistent, maladaptive inflammation in sepsis. Elevated ACBP/DBI levels in patients were strongly associated with both clinical severity and mortality across independent cohorts, suggesting its potential utility as both a prognostic biomarker and a therapeutic target. Mechanistically, ACBP/DBI is not merely a bystander but appears to function as a central driver of the pathogenic cascade via the activation of pro-inflammatory immune effectors, thereby perpetuating the cytokine storm and tissue injury that typify septic pathophysiology (Supplementary Fig. S13).

The functional relevance of ACBP/DBI was corroborated through three independent murine models—LPS-induced endotoxemia, *E. coli* infection, and CLP—each representing distinct clinical features of septic shock.^27^ In all cases, prophylactic ACBP/DBI neutralization significantly improved survival, mitigated inflammatory cytokine production, and preserved organ function. In addition, ACBP/DBI neutralization reduced splenic bacterial burden and septic shock lethality even when administered after *E. coli* challenge or CLP, suggesting that it has therapeutic effects as well. ACBP/DBI neutralization improved bacterial clearance in vivo and enhanced the phagocytic and bactericidal capacities of neutrophils and alternatively activated macrophages, indicating enhanced host defense. In addition, it appears plausible that ACBP/DBI inhibition enhances tissue tolerance, given its ability to protect tissues against sterile insults, such as ischemia-reperfusion damage of the heart and the liver,^11^ dietary and toxic insults to the liver,^11,31^ chemotherapeutic agents affecting the heart or the kidney,^18^ as well as against LPS, as shown here. Indeed, high-dimensional immune profiling and multi-organ metabolomics revealed that ACBP/DBI neutralization remodels the myeloid immune landscape and/or reinstates metabolic homeostasis in the heart, kidney, liver, lung and spleen, suggesting body-wide protective effects against septic shock.

A central strength of our work lies in its translational scope: we not only document ACBP/DBI elevation in human sepsis but also demonstrate the therapeutic efficacy of its prophylactic neutralization in preclinical models that recapitulate key features of the clinical syndrome. Importantly, we interrogated the interaction between ACBP/DBI and the only anti-inflammatory therapy shown to improve outcomes in sepsis, glucocorticoid therapy. While corticosteroids reduce systemic inflammation and improve hemodynamic stability, they paradoxically induce ACBP/DBI,^13,32^ potentially offsetting some of their therapeutic benefit. Indeed, the deleterious long-term effects of glucocorticoids -including hyperglycemia, dyslipidemia, visceral adiposity, and musculoskeletal degeneration (sarcopenia and osteopenia)- are mechanistically dependent on ACBP/DBI upregulation. In murine models, genetic deletion or antibody-mediated inhibition of ACBP/DBI abolishes all facets of metabolic syndrome and adverse body composition remodeling induced by glucocorticoids.^13,32^ In marked contrast, the beneficial anti-inflammatory effects of short-term glucocorticoid administration during septic shock, principally the mitigation of hyperinflammation, are fully preserved and in some domains enhanced by concomitant ACBP/DBI blockade. Our data suggest that, in the context of septic shock, co-administration of anti-ACBP/DBI mAb with DEX yields additive benefits across physiological, transcriptomic, and metabolomic parameters. This supports a model in which ACBP/DBI inhibition selectively uncouples the short-term anti-inflammatory effects and the long-term metabolic liabilities of glucocorticoids, enhancing the former while abrogating the latter.

Survivors of sepsis demonstrate features of accelerated aging in the context of the “post-sepsis syndrome” that includes a higher risk of major adverse cardiovascular events,^33^ a long-term exhaustion of the adaptive immune response suggestive of immunosenescence,^34^ as well as a myriad of cognitive, sensory, integumentary, musculoskeletal, respiratory and renal problems.^35,36^ It has been suggested that (some of) these long-term consequences might be mediated by cellular senescence affecting various organs, including the heart, kidney, liver and lung in mice^37–40^ and circulating blood cells in humans.^41^ In this context, it should be noted that ACBP/DBI has been causally implicated in the aging process affecting humans and model organisms^9,42^ and that ACBP/DBI neutralization has senolytic effects in mice,^18^ including the CLP murine model of septic shock, as shown here. Hence, it can be speculated, yet remains to be demonstrated, that ACBP/DBI inhibition might attenuate the long-term sequelae of sepsis.

ACBP/DBI is also called “endozepine” (a contraction of “endogenous benzodiazepine”) because it displaces the prototypic benzodiazepine diazepam from the GABA_A_ receptor (“diazepam-binding inhibitor”) and shares the pharmacological properties of benzodiazepines as a positive allosteric modulator, hence sensitizing the GABA_A_ receptor to their endogenous agonist GABA.^43–45^ Increased plasma levels of endozepines have been reported during systemic inflammation in patients.^46^ In addition, retrospective clinical observations indicate that long-term benzodiazepine use is associated with increased 90-day mortality among adult patients with sepsis compared with non-users,^47^ as well as with an increased susceptibility to bacterial or viral infection in the general population.^48^ Accordingly, diazepam administration reversed the live-preserving effects of ACBP/DBI neutralization in the CLP model of polymicrobial sepsis. These results echo other reports demonstrating that benzodiazepines enhance the susceptibility of rodents to infection,^49–51^ and indirectly validate the conjecture that ACBP/DBI mediates immunosuppression in humans.^52^ However, it will be necessary to perform clinical trials with ACBP/DBI neutralizing antibodies or small molecule antagonists to test this hypothesis.

While our findings provide compelling preclinical and translational evidence supporting ACBP/DBI as a therapeutic target in sepsis, several limitations merit consideration. First, although we validated our observations across multiple murine models and patient cohorts, the interspecies divergences in immune signaling, metabolic regulation, and cytokine response kinetics may limit the direct extrapolation of these results to human pathophysiology. Specifically, murine sepsis models, while biologically informative, do not fully capture the temporal, immunological, and genetic heterogeneity of human sepsis. Despite the multi-omics and mechanistically integrative nature of our analyses, important questions remain regarding the cell-type-specific expression patterns and intracellular signaling cascades downstream of ACBP/DBI. Dissecting these circuits at single-cell resolution in humanized or patient-derived models may reveal additional context-dependent roles and help refine the therapeutic index of ACBP/DBI targeting. Finally, although our prophylactic combination therapy experiments in mice are promising, the optimal timing, dosing regimen and clinical indications for ACBP/DBI inhibition, either as monotherapy or in combination with glucocorticoids, require validation in prospective clinical trials. While these murine models are useful to understand the drivers of pathology, further experiments are needed to determine the real therapeutic potential of ACBP/DBI inhibition. Future studies should also address potential off-target effects, durability of response, and the impact of ACBP/DBI blockade across different sepsis endotypes and comorbid metabolic conditions. To this end, human ACBP/DBI-specific antibodies crossreactive with non-human primate ACBP/DBI will need to be evaluated for their pharmacological and toxicological profile in primates before advancing to clinical trials.

## Materials and methods

### Experimental models and study participant details

#### Human cohorts

##### Discovery cohort

Clinical data were prospectively collected from critically ill patients with sepsis (*n* = 4), septic shock (*n* = 21), and patients admitted into the ICU after elective surgery (ICU Control; *n* = 18). Collection of clinical data and biological samples of septic and control patients was performed in the Intensive Care Medicine Department of Centro Hospitalar Universitário São João (Porto, Portugal). Demographic characteristics, preexisting conditions, organ function, sites of infection, microbiology results, hematologic and laboratory measurements, SAPS II score, and ICU mortality were recorded. The study was approved by the São João Hospital Ethics Committee (#03/2012), and written informed consent was obtained from each patient or next of kin. Inclusion Criteria: (i) Sepsis and Septic Shock group: Adult patients (age >18 years) with sepsis or septic shock according to Third International Consensus Definitions for Sepsis and Septic-Shock^3^ (Sepsis-3); (ii) ICU Control group: Adult patients (age >18 years) admitted after elective surgery, without clinical or analytical evidence of infection or hypoperfusion. Exclusion criteria: (i) patients undergoing immunosuppressive or chemotherapy protocols; (ii) patients with immunoproliferative and autoimmunity disorders. Samples of peripheral blood (5 mL) were collected in K3EDTA tubes during the first 24 h, 48 h, and 72 h post-admission to ICU and centrifuged (4 °C, 3000 rpm, 15 min). The plasma (supernatant) was then collected and cryopreserved at −80 °C. Plasma of 24 h post-admission to ICU was thawed for ACBP ELISA quantification. Estimated glomerular filtration rate (eGFR) values were calculated using the 2021 CKD-EPI eGFRcr equation.^53^

##### Validation cohort

Patients older than 18 years meeting the diagnostic criteria for sepsis according to the Third International Consensus Definitions for Sepsis and Septic-Shock^3^ (Sepsis-3) were enrolled in this study upon arrival to Emergencies (*n* = 303). Sepsis was initially identified based on SOFA criteria (tachypnea, altered level of consciousness, and hypotension) and then thoroughly corroborated following the International Sepsis Definitions Conference criteria^54^ and classified according to their severity in sepsis and septic shock (operationally defined as requiring vasopressor therapy to maintain a mean arterial blood pressure of >65 mmHg and an increased plasma lactate level of >2 mmol/L). Patients were classified according to their outcome into survivors or *exitus*. Blood samples were promptly obtained from patients upon their admission to the Emergency Department of the University Hospital La Paz (Madrid, Spain) within the first hour of their arrival, and plasma were isolated by standardized procedures. As controls, healthy volunteers (HV, *n* = 121) were recruited in person from the Blood Donor Services of La Paz University Hospital. All of them were free of pathogen colonization and recruited randomly with age and gender matching.^26^

##### Human ACBP (hACBP) quantification

ACBP concentrations were measured using an enzyme-linked immunosorbent assay (ELISA) with a commercial kit from Abnova (Cat. No. ABNOKA6327, Lot No. KD6867), according to the manufacturer’s instructions. Briefly, patient plasma samples were diluted 1:40 in standard diluent prior to the assay. The TMB substrate was added, and the enzymatic reaction was allowed to proceed for 12 min. Absorbance was measured at 450 nm using a Victor NIVO microplate reader (PerkinElmer, Waltham, MA, USA).

##### Statistical analyses

ACBP levels among different groups (Controls, Sepsis, Septic Shock, and SIRS) were compared using the Kruskal–Wallis test for unpaired, non-parametric comparisons across multiple groups. For multiparametric analyses, clinical variables obtained from participants’ medical records and routine blood tests were analyzed as raw values. The Wilcoxon signed-rank test was used for pairwise comparisons between the Control and Septic groups or between Survivors and non-survivors (*Exitus*). When applicable, *p* values were adjusted using the false discovery rate (FDR) method. Spearman correlation matrices were computed using the rcorr() function from the Hmisc package (v5.1-3), clustered with hclust() from the stats package (v4.3.3), and visualized using the corrplot package (v0.94). Survival analysis was performed using the Kaplan–Meier method, with group differences assessed via the log-rank test. Kaplan–Meier survival curves were generated with survfit() from the survival package (v3.5-5), stratified by median hACBP values, and visualized using ggsurvplot() from the survminer package (v0.5.0), including survival probabilities, *p* values, and risk tables. A multivariable logistic regression model was fitted using the glm() function with a binomial family to evaluate associations between in-hospital mortality, hACBP levels, and clinical parameters or scoring systems. Model fit and coefficient significance were assessed using Wald tests, and fitted probabilities were extracted for further analysis. Receiver-Operating Characteristic (ROC) curves were constructed using the pROC package (v1.18.5) to assess the predictive performance of biomarkers and scoring systems (e.g., SAPS II, CRP, PCT). Area Under the Curve (AUC) values were calculated using DeLong’s method. All analyses were performed using R software (version 4.4.1, 2024-06-14 ucrt; R Foundation for Statistical Computing; https://www.R-project.org/).

### Animal models

Mice were housed under a 12-h light/dark cycle in a temperature-controlled environment and had *ad libitum* access to a standard chow diet (#A04, Safe). All animal experiments were conducted in accordance with the recommendations of the Federation of European Laboratory Animal Science Associations (FELASA) and were approved by the local ethics committee (protocols #46833-202401111607908-v4 and #54338-2025031618187343-v4).

#### Mouse model of endotoxemia

Twelve-week-old male C57Bl/6J mice were purchased from Envigo (Gannat, France) and acclimatized for 1 week prior to experimentation. Mice received an intraperitoneal (i.p.) injection of either a mouse monoclonal anti-ACBP neutralizing antibody (α-ACBP; 2.5 or 5 mg/kg body weight, clone 7a, Fred Hutch Antibody Technology) or an isotype control antibody (IgG2a; 2.5 or 5 mg/kg body weight, BioXcell, NH, USA). Subsequently, each group received an i.p. injection of either phosphate-buffered saline (PBS) or lipopolysaccharide (LPS; 10–20 mg/kg body weight; Sigma, #L2630). Body temperature was monitored at various time points using rectal thermometry, and survival was assessed every 12 h for 7 days. In a separate set of experiments, mice received a co-administration of dexamethasone (DEX; 10 mg/kg body weight, Rapidexon 2 mg/mL, dissolved in PBS) or PBS 1X via intraperitoneal injection, as specified, or an intravenous (i.v.) injection of recombinant ACBP/DBI protein (8 mg/kg body weight).

#### Induction of E. coli peritonitis

Twelve-week-old male C57Bl/6J mice (Envigo, Gannat, France) were acclimatized for 1 week prior to experimentation. Peritonitis was induced by intraperitoneal (i.p.) injection of varying doses of viable *Escherichia coli* (ATCC 25922), diluted in PBS 1X, as previously described.^55,56^ Briefly, *E. coli* was cultured in Luria-Bertani medium (Sigma, L7275) at 37 °C to the mid-logarithmic growth phase, then washed twice with PBS 1X. Bacterial concentration was estimated by measuring absorbance at 600 nm (A_600_) using a spectrophotometer. Serial dilutions of the final inoculum were plated on LB agar and incubated overnight at 37 °C to verify the number of viable bacteria injected. In the initial set of experiments, survival was monitored following infection with increasing doses of *E. coli*, with or without co-administration of α-ACBP monoclonal antibody (clone 7a, Fred Hutch) or its isotype control (IgG2a, BioXcell). In subsequent experiments, mice infected with *E. coli* and treated with α-ACBP were sacrificed at 1-, 6-, and 24-h post-infection to collect blood and organs. Peritoneal lavage fluid (PLF) was collected by flushing the peritoneal cavity with 5 mL of sterile PBS 1X using a 21-gauge needle and immediately placed on ice. Blood was collected by cardiac puncture into sterile heparin-coated tubes (Microvette® CB 300 LH, Sarstedt, Nümbrecht, Germany) and kept on ice. Spleen and kidney were harvested and transferred into Precellys tubes (CK28 Hard Tissue Homogenizing, 2.8 mm zirconium oxide beads; Precellys, Bertin Technologies) containing 600 µL of PBS, placed on ice, and homogenized using a Precellys 24 tissue homogenizer (Bertin Technologies, Montigny-le-Bretonneux, France). Bacterial loads in PLF, blood, and tissue homogenates were quantified by plating serial dilutions of each sample onto LB agar plates, followed by overnight incubation at 37 °C. Colony-forming units (CFUs) were counted using an automated method based on image analysis of scanned LB plates, performed in R software (https://www.r-project.org/), using primarily the *EBImage* package (https://www.bioconductor.org/) in combination with the Python *scikit-image* library (https://scikit-image.org/) accessed through the *reticulate* package. Briefly, the red component of the image was extracted, and individual plates were automatically detected and cropped using Hough transform. The resulting images were then iteratively segmented by applying a sigmoid transformation following noise reduction via a Lee Filter. Segmented objects (i.e colonies) were thereafter separated and labeled using a watershed algorithm. Features such as colony count and area were computed and finally reported per plate quadrant, defined by their angular coordinates. In a separate set of experiments, mice received an intraperitoneal (i.p.) injection of either a mouse monoclonal anti-ACBP neutralizing antibody (α-ACBP; 10 mg/kg body weight, clone 7a, Fred Hutch Antibody Technology) or an isotype control antibody (IgG2a; 10 mg/kg body weight, BioXcell, NH, USA), 30 min after *E. coli* challenge.

#### In vivo depletion of macrophages and neutrophils

Twelve-week-old male C57Bl/6J mice (Envigo, Gannat, France) were treated with the monoclonal antibody against ACBP (α-ACBP; 5 mg/kg body weight, i.p.) administered twice during the week prior to *E. coli* challenge (2.5 × 10⁶ CFU/mouse, i.p.). For macrophage depletion, mice received an intravenous injection of clodronate liposomes (100 µL/10 g body weight; Liposoma B.V., The Netherlands) or control liposomes 48 h before infection. Neutrophil depletion was achieved by intraperitoneal injections of anti-mouse Ly6G antibody (clone 1A8, 50 µg/mouse, #BE0075-1, BioXcell) or isotype IgG2a control (clone 2A3, #BE0089, BioXcell) at 36 h and 12 h before *E. coli* injection.^57,58^ Additionally, α-ACBP (2.5 mg/kg) was administered twice more in the hours preceding *E. coli* challenge. At 24 h post-infection, spleen and kidney were harvested, homogenized, and plated to quantify bacterial colony-forming units (CFUs), as previously described. Depletion of macrophages (CD11b⁺F4/80⁺) and neutrophils (CD11b⁺Ly6G⁺) was confirmed by flow cytometry. Briefly, spleens were collected in RPMI medium and mechanically dissociated through a 70 µm cell strainer. Cell suspensions were centrifuged (400 × *g*, 5 min, 4 °C), treated with Red Blood Cell (RBC) lysis buffer (Cat. 50-112-9743, Invitrogen), then washed and resuspended in FACS buffer (0.5% BSA in PBS). Peripheral blood mononuclear cells (PBMCs) were isolated from whole blood using Ficoll-Paque Plus density gradient centrifugation (GE17-1440-02, Sigma-Aldrich). The PBMC layer was collected, washed twice in PBS, and resuspended in FACS buffer. Following Fc receptor blocking (anti-CD16/CD32, 10 min, 4 °C), spleen and PBMC cells were stained for 30 min at 4 °C in the dark with the following antibodies: F4/80-FITC (REAfinity™, Miltenyi Biotec), Ly6G-PE (clone 1A8, BD Pharmingen™), and CD11b-APC (clone M1/70, BioLegend). After staining, cells were washed twice and fixed with IC Fixation Buffer (eBioscience, Cat. 00-8222-49, Invitrogen) for 15 min at 4 °C in the dark. Fixed cells were then washed and resuspended in FACS buffer prior to acquisition on a MACSQuant® Analyzer 16 flow cytometer (Miltenyi Biotec, Bergisch Gladbach, Germany). Flow cytometry data were analyzed using FlowJo software (version 10.6.1).

#### Cecal ligation and puncture (CLP)-induced high-grade sepsis

Eight-week-old male C57Bl/6J mice (Envigo, Gannat, France) were purchased and allowed to acclimate for 1 week. Mice were randomly assigned to treatment groups and administered either a monoclonal antibody against ACBP/DBI (α-ACBP; 4 h and 1 h prior to surgery; or 6 h and every 48 h after surgery) or its isotype control, in combination with dexamethasone (DEX) or PBS 1X, administered 1 h before CLP, as previously described.^59,60^ Sepsis was induced by CLP under isoflurane anesthesia. Briefly, the cecum was exteriorized, ligated at 75% of its length using non-absorbable 4-0 silk suture, and punctured once through-and-through with a 21-gauge needle. A small amount of fecal content was gently extruded, and the cecum was returned to the abdominal cavity. Sham-operated mice underwent the same procedure, including treatment injections, but without ligation or puncture. Mice that died within the first 24 h post-surgery were considered to have succumbed to perioperative complications and were excluded from further analysis. Post-operative monitoring included daily weighing and 2–3 daily assessments for clinical signs of sepsis, using the Murine Sepsis Score (MSS), as described.^61^ Body temperature was measured at various time points using rectal thermometry. Survival was monitored every 12 h for 7 days. Humane endpoints were defined as a total MSS of 15 or the maximum score in any individual category, according to established guidelines.^61^ At 6 or 24 h post-CLP, PFL, spleen, kidney, and liver tissues were harvested, homogenized, and plated to quantify bacterial CFUs, as previously described. In another set of experiments, mice received the monoclonal antibody against ACBP/DBI or its isotype, in combination with diazepam or its vehicle. Diazepam (Atnahs, Valium® 1% drinkable solution) or the matching vehicle (40% EtOH, 500 mg/mL propylene glycol [PG, Sigma, #294004] in ddH2O) was diluted to 0.8 mg/mL in a 1:1 PG:ddH2O mix and injected intraperitoneally (i.p.) at a dose of 4 mg/kg. This dose corresponds to the clinically relevant dose of 20 mg per day for a 60 kg human adult, following the thumb rule that per-weight doses in mice must be divided by 12.3 (based on body surface area) to yield per-weight doses in humans.

#### Induction of ACBP/DBI knockout

Male C57Bl/6J mice homozygous for a floxed exon 2 of Acbp/Dbi gene and a tamoxifen-inducible, ubiquitously expressed Cre recombinase transgene (genotype: UBC- cre/ERT2::Acbp/Dbif/f, abbreviated as Acbp/Dbi^−/−^) were compared to “wild type” (WT) control mice homozygous for the floxed allele but lacking the Cre transgene (genotype: Acbp/Dbif/f). Both groups were injected with tamoxifen (i.p. 75 mg/kg body weight daily during 5 days, Sigma, #T5648). Prior to injection, tamoxifen was diluted in corn oil (90%) + ethanol (10%) at a concentration of 20 mg/mL and shaken overnight at 37 °C.^62^ Acbp/Dbi^−/−^ and WT mice were subjected to CLP surgery as previously described. Body temperature was measured at multiple time points using rectal thermometry, and survival was monitored every 12 h for up to 7 days.

#### Blood and tissue collection

For molecular analyses, organs were collected on the day of euthanasia (by cervical dislocation) and immediately snap-frozen in liquid nitrogen. Blood was collected in heparin-lithium capillary tubes (Microvette® CB 300 LH, Sarstedt, Nümbrecht, Germany) and kept on ice until centrifugation at 4000 × *g* for 10 min. After centrifugation, plasma was separated and snap-frozen. Both tissues and plasma were stored at −80 °C until further use. For histological analyses, freshly collected tissues were fixed overnight at 4 °C in 4% buffered formaldehyde (Sigma, #F8775), then transferred to 70% ethanol before paraffin embedding.

### Mean arterial pressure (MAP) measurement

MAP was measured in mice using the BP-2000 non-invasive blood pressure system (Visitech Systems, Inc.), which employs transmission photoplethysmography to determine diastolic and systolic blood pressure. Body temperature was maintained at 37 °C using a temperature-controlled heating platform. Measurements were taken multiple times before the CLP procedure to establish baseline values during the acclimation period, and again 24 h after the CLP. For each mouse, a minimum of seven stable readings were recorded at each time point to ensure accuracy.^63^

### Ultrasound assessment of cardiac function

Cardiac function was assessed using non-invasive 2D echocardiography (Vevo 3100, Fujifilm VisualSonics Inc.) at 12 h post-LPS injection and 24 h post-CLP. Briefly, mice were lightly anesthetized with isoflurane (3–4% for induction, 0.5% for maintenance), and body temperature was maintained at 37 °C using a temperature-controlled heating platform. Mice were placed in the supine position, with limbs in contact with non-invasive electrocardiogram leads to monitor heart rate. Pre-warmed ultrasound transmission gel was applied to the shaved chest to obtain cardiac images in the parasternal long axis view using a high-resolution 55 MHz linear-array probe. M-mode tracings at the level of the papillary muscles were used to measure left ventricular wall thickness and internal dimensions during systole and diastole, as previously described.^64^ Ventricular volumes and myocardial mass were calculated using the Teichholz and Troy formulas, respectively. Ejection fraction was determined to evaluate systolic function. For each mouse, parameters were averaged over at least three stable cardiac cycles.

### RT-qPCR of primary isolated hepatocytes from *Acbp/Dbi*^*−/−*^ mice

#### Primary mouse hepatocyte isolation and culture

Hepatocytes were isolated from 12-week-old male control (WT) and knockout (*Acbp/Dbi*^−/−^) mice by perfusion via the inferior vena cava. The perfusion solutions included Hank’s Balanced Salt Solution (HBSS, 1X; Gibco, # 14180046) supplemented with 1 mM HEPES (pH 7.4) and 0.2 mM EGTA, followed by William’s E medium (Gibco, #22551-022) containing 7.5 mg collagenase from *Clostridium histolyticum* (Type IV; 0.5–5.0 FALGPA units/mg solid; ≥125 CDU/mg solid, Sigma). Following digestion, the cell suspension was filtered through a 100 µm cell strainer and centrifuged twice at 30 × *g* for 5 min at 4 °C. Cells were then resuspended in culture medium for cell attachment consisting of DMEM/F12 supplemented with 20 mM HEPES (pH 7.4), 5 mM glucose, 10% fetal bovine serum (FBS), 5 mg/mL BSA (Bovine Serum Albumin, Euromedex, #04-100-812-E), 100 U/mL penicillin, and 100 µg/mL streptomycin. Hepatocytes were further purified by density gradient centrifugation using an isotonic Percoll solution (GE Healthcare Bio-Sciences AB, Uppsala, Sweden). Cell viability was assessed by Trypan blue exclusion. Purified hepatocytes were plated in 12-well plates and cultured overnight to allow cell attachment before experimental procedures.^65^

#### RNA extraction and gene expression analyses

Primary hepatocytes were treated in vitro with two concentrations of LPS (250 ng/mL and 1000 ng/mL; Sigma, L2630). After 6 h, cells were lysed in QIAzol lysis reagent, and total RNA was extracted using the RNeasy Mini Plus Kit (Qiagen) according to the manufacturer’s protocol. RNA purity and concentration were measured using a NanoDrop™ spectrophotometer (Thermo Fisher Scientific). One microgram of total RNA was reverse transcribed using the SuperScript™ VILO™ cDNA Synthesis Kit (Invitrogen). Quantitative real-time PCR was performed using a StepOnePlus Real-Time PCR System (Applied Biosystems, Thermo Fisher Scientific) with Power SYBR™ Green Master Mix (Applied Biosystems) or specific TaqMan probes (Thermo Fisher Scientific), following the manufacturer’s instructions (Supplementary Table S6). The PCR cycling parameters were as follows: 95 °C for 1 min, followed by 40 cycles of 95 °C for 15 s and 60 °C for 1 min. Each sample was run in duplicate, and expression levels were normalized to the housekeeping gene *36b4* or *Ppia*. Replicates were averaged, and relative gene expression was calculated using the ΔΔCt method.^11^

### Bone marrow-derived neutrophils (BMDNs) and their effects on bacteria

#### Bone marrow isolation and neutrophil purification

Bone marrow cells were isolated from the femurs and tibiae of 8–12-week-old C57BL/6 mice as previously described.^66,67^ Briefly, bones were dissected, cleaned, and flushed using a 23-gauge needle with PBS containing 2 mM EDTA and 2% FBS. The cell suspension was passed through a 70-μm nylon mesh sieve. Red blood cells were lysed using Red Blood Cell (RBC) Lysis buffer (Invitrogen, Cat. 50-112-9743). After centrifugation, bone marrow cells were washed and counted. Neutrophils were isolated by density gradient centrifugation using Histopaque 1119 and 1077 (Sigma-Aldrich, catalog #11191 and #10771). Bone marrow cells were layered onto the gradients and centrifuged at 872 × *g* for 30 min at room temperature without brake. Neutrophils were collected from the 1119/1077 interface, washed, and resuspended in RPMI 1640 supplemented with 10% FBS. Cell yield and purity were determined by flow cytometry, typically yielding >80% Ly6G+ neutrophils with viability >80%.

#### In vitro cell culture

Isolated neutrophils were plated in duplicate on 12-well plates and cultured at 37 °C with 5% CO₂ in RPMI 1640 containing 10% FBS. Cells were treated with monoclonal α-ACBP antibody (5 µg/mL) or isotype control IgG2a for 16 h.

#### Bacteria killing assay

*Escherichia coli* (ATCC 25922) was cultured in Luria-Bertani medium (LB; Sigma, L7275) at 37 °C to mid-logarithmic phase, washed twice with PBS 1X, and resuspended in RPMI without complement. Bacterial concentration was estimated by measuring absorbance at 600 nm (A600). For opsonization, bacteria were incubated with 10% FBS in PBS1X, rotating end-over-end (6 rpm) for 20 min at 37 °C. The bacterial killing assay was performed as previously described.^68,69^ Briefly, 1 × 10⁷ BMDNs were resuspended in 200 μL RPMI 1640 supplemented with 2% FBS. Twenty microliters of normal mouse serum were added to the cells, followed by 220 μL of opsonized bacteria (multiplicity of infection [MOI] of 10). Neutrophils and bacteria were co-cultured in a total volume of 440 μL (1:1 ratio) while rotating end-over-end (6 rpm) for 45 min at 37 °C. The reaction was stopped by adding 440 μL of ice-cold PBS1X, followed by centrifugation (5 min, 100 × *g*, 4 °C). Supernatants were collected, diluted 1:200, plated on LB agar plates, and incubated overnight for colony-forming unit (CFU) enumeration. Cell pellets were lysed with 0.2% Triton X-100 (Euromedex, 9002-93-1), serially diluted, and plated similarly. Control tubes containing bacteria alone were processed in parallel.

#### Flow cytometry

For all samples, cell viability was assessed by incubation with LIVE/DEAD™ Fixable Yellow dye (Thermo Fisher Scientific) for 15 min at 4 °C. After PBS washing, Fc receptors were blocked with anti-mouse CD16/CD32 (clone 2.4G2, Mouse BD Fc Block™, BD Biosciences) diluted in FC buffer (0.5% BSA in PBS) for 10 min at 4 °C. After washing, pre-fixation surface staining was conducted with the following antibody panel diluted in BD brilliant buffer (50% volume) (BD Biosciences), complemented with FC buffer with 25 min incubation at 4 °C.

##### Panel profiling

Cell surface staining was performed with the following fluorochrome-conjugated antibodies: anti-CD11b FITC (clone M1/70, BioLegend, Cat# 101206), anti-Ly-6G PE (clone 1A8, BD Biosciences, Cat# 551461), and anti-CD45 APC-Vio770 (clone 30-F11, Miltenyi Biotec). Cells were then fixed and permeabilized in eBioscience™ Foxp3/Transcription Factor Staining Buffer (Thermo Fisher Scientific). Intracellular staining was performed with an anti-ACBP monoclonal primary antibody (clone 35C7F1, ProteóGenix, Schiltigheim, France) followed by a secondary Alexa Fluor™ Plus 647- conjugated antibody (Invitrogen; Cat# A32728). After final washes, cells were resuspended in 200 µL FC buffer and acquired on a BD FACSDiscover™ S8 Cell Sorter (BD Biosciences).

### Bone marrow-derived macrophages (BMDMs) and their effects on bacteria

#### Isolation and culture of BMDMs

Bone marrow cells were isolated from the femurs and tibiae of 8–12-week-old C57BL/6 mice and homozygous Gabrg2^F77I/F77I^ mice (bearing a point mutation F77I in the binding site of ACBP/DBI in the gamma-aminobutyric acid A Receptor γ2 subunit), as previously described.^70^ Briefly, bones were dissected, cleaned, and flushed using a 23-gauge needle in PBS containing 2 mM EDTA and 2% FBS and passed through a 70-μm nylon mesh sieve cell strainer. Red blood cells were lysed using a Red Blood Cell (RBC) Lysis buffer (Invitrogen, Cat. #50-112-9743). After centrifugation, cells were washed, counted, and plated in RPMI 1640 with GlutaMax (Sigma, Cat. #61870010), supplemented with 10% FBS. Cells were differentiated for 7 days in the presence of 50 U/mL murine M-CSF (PeproTech, Cat. #315-02). On day 7, cells were detached and characterized by flow cytometry, yielding >70–80% F4/80⁺ cells and viability >80%.

#### In vitro cell culture

On day 7 of differentiation, bone marrow-derived macrophages (BMDMs) were cultured in RPMI 1640 with GlutaMax +10% FBS and polarized for 24 h under three conditions: classically activated with lipopolysaccharide (LPS, 100 ng/mL; Sigma, L2630) and murine IFN-γ (25 ng/mL; PeproTech, Cat. #315-05); alternatively activated with murine IL-4 (25 ng/mL; PeproTech, Cat. #214-14) and murine IL-13 (25 ng/mL; PeproTech, Cat. #210-13); or maintained in media alone. For treatment, cells in each condition were incubated with either mouse IgG2a isotype control (5 µg/mL) or α-ACBP antibody clone 7G4a (5 µg/mL) for 16 h.

#### Bacteria killing assay

*Escherichia coli* GFP (ATCC 25922GFP) was cultured in Luria-Bertani medium (LB; Sigma, L7275) at 37 °C to mid- logarithmic growth phase and washed twice with PBS1X and resuspended in uncomplemented RPMI. The concentration of bacteria was determined by spectrophotometric reading at 600 nm. The bacterial killing assay was performed as previously described.^71^ Briefly, culture medium was removed, and bacteria were added to macrophages at a multiplicity of infection (MOI = 10) for 10 or 20 min. Cells were then washed once with PBS1X and incubated with gentamicin (100 μg/ml, Sigma-Aldrich, G1397) in RPMI for 10 min to eliminate extracellular bacteria. After PBS washing, cells were maintained in antibiotic-free RPMI supplemented with 10% FBS for 1 or 2 h before proceeding with confocal staining or LB agar plating.

#### Confocal microscopy

For fluorescence microscopy, cells were fixed with 4% paraformaldehyde (PFA) containing Hoechst 33342 (1:5000, Thermo Fisher Scientific, H3570) for 20 min at room temperature with gentle agitation and protected from light. Cells were rinsed twice with PBS, permeabilized with 0.1% Triton X-100 (Euromedex, 9002- 93-1) for 10 min at room temperature in the dark, then rinsed again. F-actin was stained using Phalloidin-AF568 (Invitrogen, A12380) diluted 1:200 in PBS with 1% BSA for 30 min in the dark at room temperature. After three washes with PBS, automated image acquisition was performed using the ImageXpress® Micro Confocal High- Content Imaging System (Molecular Devices) with a Plan APO 20X objective (Nikon). The effective intracellular *E. coli* GFP killing rate by BMDMs was determined by calculating the percentage of living bacteria after 1 h of culture with respect to the total amount of internalized bacteria per cell, after image segmentation by means of a custom macro edited within the Custom Module Editor (CME) of MetaXpress software.

#### LB agar plating

For internalization assays, macrophages were lysed immediately after the gentamicin step using 0.2% Triton X-100. Lysates were serially diluted (1:10, four dilutions per sample), plated on LB agar, and incubated overnight at 37 °C for CFU quantification as reported. For clearance assays, following the gentamicin step, cells were cultured for 2 h in antibiotic-free RPMI supplemented with 10% FBS. Cells were lysed with 0.2% Triton X-100. Lysates were diluted and plated as described above for CFU enumeration.

#### Flow cytometry

For all samples, cell viability was assessed by incubation with LIVE/DEAD™ Fixable Yellow dye (Thermo Fisher Scientific) for 15 min at 4 °C. After PBS washing, Fc receptors were blocked with anti-mouse CD16/CD32 (clone 2.4G2, Mouse BD Fc Block™, BD Biosciences) diluted in FC buffer (0.5% BSA in PBS) for 10 min at 4 °C. After washing, pre-fixation surface staining was conducted with the following antibody panel diluted in BD brilliant buffer (50% volume) (BD Biosciences), complemented with FC buffer with 25 min incubation at 4 °C.

##### Panel profiling of tissue-infiltrating immune cells with a focus on myeloid cells.

Cell surface staining was performed with the following fluorochrome-conjugated antibodies: anti-MHC-II (I-A/I-E) BUV496 (clone M5/114.15.2, BD Biosciences, Cat#750281), anti-CCR2 BV421 (clone SA203G11, BioLegend, Cat#150605), anti-CD38 BV711 (clone 90/CD38, BD Biosciences, Cat# 740697), anti-CD206 BV785 (clone C068C2, BioLegend, Cat# 141729), anti-CD11b FITC (clone M1/70, BioLegend, Cat# 101206), anti-F4/80 PE-Vio770 (clone REA126, Miltenyi Biotec), and anti-CD45 APC-Vio770 (clone 30-F11, Miltenyi Biotec). Cells were then fixed and permeabilized in eBioscience™ Foxp3/Transcription Factor Staining Buffer (Thermo Fisher Scientific). Intracellular staining was performed with an anti-ACBP monoclonal primary antibody (clone 35C7F1, ProteóGenix, Schiltigheim, France) followed by a secondary Alexa Fluor™ Plus 647-conjugated antibody (Invitrogen; Cat# A32728). After final washes, cells were resuspended in 200 µL FC buffer and acquired on a BD FACSDiscover™ S8 Cell Sorter (BD Biosciences).

### Biochemical assays

#### Renal function estimation

Renal parameters were evaluated on the day of sacrifice on one drop of tail vein blood with an epoc® Blood Analysis System (Siemens Healthineers, Erlangen, Germany). The system provided real-time measurement of the following parameters: pH, pCO₂, pO₂, Na⁺, K⁺, Cl^−^, Ca²⁺, hematocrit (Hct), creatinine, and total CO₂ (TCO₂). In addition, the device calculated several secondary parameters based on the measured values, including: bicarbonate (HCO₃^−^), base excess (BE), oxygen saturation (SO₂), calculated hemoglobin (cHgb). Calculations were performed automatically using the system’s internal algorithms. Blood glucose was measured independently using Accu-Chek® Performa strips (Roche Diagnostics) and a handheld glucometer, while blood lactate was measured using the Lactate Plus Meter (Nova Biomedical), according to the manufacturer’s protocols. A small drop of whole blood was applied to each test strip immediately after collection for both devices. Blood Urea Nitrogen (BUN) was measured in plasma using the Urea Nitrogen (BUN) Colorimetric Detection Kit, following the manufacturer’s instructions (Thermo Fisher, #EIABUN). The glomerular filtration rate was estimated from BUN and creatinine values and the weight of each animal with a formula adapted to rodents^72^: eGFR_µL/min_ = 5862 × Weight_g_^0.695^ × Creatinine_μM_^−1.150^ × Urea_mM_^−0.391^.

#### Quantitation of hepatic transaminases in plasma

Alanine aminotransferase (ALT) and Aspartate aminotransferase (AST) activities were determined by colorimetric kits (Randox) accordingly with the manufacturer’s instructions.^31^

#### Cardiac Troponin I measurement

Mouse cardiac Troponin I levels were measured in plasma samples using the Mouse Troponin I ELISA Kit (Abcam, catalog #ab285235), following the manufacturer’s instructions. Absorbance was read at 450 nm using the VICTOR Nivo Multimode Microplate Reader (PerkinElmer).

#### Mouse IL-1β, TNFα and IL-6 measurements

For cytokine validation, plasma was separated as previously described. Levels of interleukin-6 (IL-6), interleukin-1 beta (IL-1β), and tumor necrosis factor alpha (TNF-α) were measured using mouse-specific ELISA kits from Sigma-Aldrich: IL-6 (catalog #RAB0309), IL-1β (catalog #RAB0274), and TNF-α (catalog #RAB0477). All samples and standards were run in duplicate, and absorbance was read at 450 nm using the VICTOR Nivo Multimode Microplate Reader (PerkinElmer).

#### Murine ACBP (mACBP) determination

Mouse plasma was obtained from blood samples collected in lithium heparin tubes and centrifuged at 4000 × *g* for 10 min at 4 °C. Cell supernatants were collected and stored at −80 °C until measurement. ACBP/DBI concentrations were measured using an ELISA assay as previously reported.^73^ Briefly, high-binding 96-well plates (Corning) were coated with 100 µL/well of anti-ACBP/DBI capture antibody (1 µg/mL, diluted in PBS, MBS2005521, MyBioSource) and incubated overnight at 4 °C. After washing, plates were blocked with 1% BSA in PBS-Tween 20 for 2 h at RT. Samples (murine plasma 1/20) and standards were added in 100 µL volumes and incubated for 2 h at RT. Plates were washed and incubated with 100 µL of the detection antibody (1 µg/mL) for 1 h at RT, followed by incubation with HRP-conjugated avidin (1/1000 for murine) for 30 min. After washing, 100 µL of TMB substrate was added and incubated in the dark for 10–30 min, followed by 50 µL of stop solution (2 M H_2_SO_4_). Absorbance was read at 450 nm using the VICTOR Nivo Multimode Microplate Reader (PerkinElmer).

### Inflammatory cytokine panel

Plasma cytokine concentrations were determined using a proximity extension assay with the Target 48 Mouse Cytokine panel (Olink, #93400) according to the manufacturer’s instructions. Briefly, 1 µL of plasma from fresh aliquots stored at −80 °C was thawed and incubated for 16 h at 4 °C in an incubation mix containing cytokine-specific antibody pairs, each coupled to forward and reverse probes. Extension of the complementary probes occurred on a SimpliAmp thermal cycler (Thermo Fisher, #A24811) and was possible only when both antibodies corresponding to a single cytokine were in close proximity, binding to neighboring epitopes on the target cytokine. For detection, an IFC 48.48 microfluidic chip (Olink, #93007) was primed and loaded with the samples and probes using an MX controller (Standard BioTools), and real-time PCR was performed on a BioMark HD system (Standard BioTools). PCR data analysis was conducted using the BioMark HD Real-Time PCR Analysis software (Standard BioTools), with automatic (global) Ct threshold determination set using the following parameters: quality threshold = 0.5, linear baseline correction. Data processing, quality control, and determination of absolute concentrations were performed using the Olink® NPX Signature software (v1.13.0). The absolute concentrations were imported into R (version 4.3.3), and log2-fold change-transformed data were visualized using the ComplexHeatmap package (version 2.16.0). The distribution of log2-transformed values was tested for normality using the Shapiro–Wilk test. Cytokines that followed a normal distribution across the four groups were analyzed using a two-way analysis of variance (ANOVA), followed by Tukey’s HSD test for pairwise comparisons. Cytokines that did not follow a normal distribution were analyzed using the Kruskal–Wallis test, followed by Dunn’s post hoc test, with Benjamini-Hochberg correction applied for multiple comparisons.

### Histopathology

#### Tissue staining

Paraffin-embedded kidneys were cut into 5 μm-thick sections and stained with hematoxylin-eosin safranin (HES) for structural evaluation or processed for immunohistochemistry (IHC) staining. Slides were scanned using an AxioScan Z1 microscope (Carl Zeiss, Jena, Germany) and visualized using QuPath open source software (v. 0.5).^74^

#### Kidney pathological score

Morphological kidney damage was evaluated on HES-stained sections using a semiquantitative scoring system applied to the entire kidney cross-section. Tubular injury was assessed blindly and independently by two experienced evaluators based on the following criteria: tubular necrosis, brush border loss, tubular dilatation, tubular cast formation, distal nephron damage, widening of the interstitium, degeneration, regeneration, and inflammation.^75^ Degeneration was scored in non-necrotic proximal tubules and included cellular swelling and cytoplasmic vacuolization. Regeneration was scored in tubules previously affected by necrosis and reflected the extent of relining by undifferentiated epithelial cells and their degree of differentiation. Inflammation was determined by the density and distribution of inflammatory cells throughout the kidney. All parameters were graded on a 0–4 scale, where 0 indicated no damage; 1, damage limited to individual tubules; 2, involvement of small groups of tubules; 3, confluent damage at the corticomedullary junction; and 4, extension of damage into the outer cortex, potentially reaching the kidney surface. Average scores from both evaluators were used for analysis, and statistical comparisons were performed using two-way ANOVA followed by estimation of marginal means for pairwise comparisons.

#### Liver pathological score

The severity of hepatic injury was evaluated on HES-stained liver sections using a semiquantitative scoring system applied blindly and independently by two experienced evaluators. Liver pathology was assessed based on the extent and distribution of hepatocellular necrosis, inflammatory infiltrates, and vascular congestion.^11^ Necrosis was graded on a 0–4 scale, where 0 indicated the absence of necrotic infiltrates; 1 represented small, isolated foci of necrotic hepatocytes occurring between intact cells or surrounding individual hepatocytes; 2 denoted larger necrotic foci consisting of approximately 100 necrotic cells or involving clusters of roughly 30 hepatocytes; 3 corresponded to necrosis affecting about 10% of the hepatic cross-section; and 4 indicated severe injury involving approximately 30% of the hepatic tissue. Inflammation was evaluated according to the density and distribution of inflammatory cells within the parenchyma and portal regions, while congestion was assessed by examining dilation and engorgement of hepatic sinusoids and central veins. The final pathological score for each sample was obtained by averaging the two independent evaluations, and statistical comparisons between groups were performed using a two-way ANOVA followed by pairwise comparisons based on estimated marginal means.

#### Lung pathological score

The lung injury score from HES-stained slides was measured by a scoring system as described.^76^ Briefly, the lung injury score was categorized into three levels of edema and three levels of immune cell infiltration: 0 for no edema and infiltration, 1 for mild edema and infiltration, 2 for moderate edema and infiltration, and 3 for severe edema and infiltration. A score was given by the pathologist, ranging from 0 to 6.

#### IHC of Ly6G+, F4/80+, and p21+ cells

To determinate the abundance of neutrophils, macrophages, and p21+ cells, lung, liver, and kidney sections from fixed paraffin blocks were immunohistochemically stained according to standard procedures using an antibody anti-mouse Ly6G, anti-mouse F4/80, and anti-mouse p21. In all cases, quantification of the Ly6G^+^, F4/80^+^, or p21^+^ staining was done on the QuPath analysis software after blinding the sample names. Color deconvolution was applied to define “DAB” and “hematoxylin” channels, corresponding to the brown and blue colors, respectively. Positive signals were detected with a simple threshold on the DAB-channel intensity, after manual removal of artefactual detections (interstitial or non-tissue detections). In the liver and lung, this method was applied to the whole tissue, while in the kidney, 10 regions of interest were defined per animal. The average value in the regions of interest was plotted for each animal, and analyzed one-way ANOVA test followed by estimation of the marginal means for pairwise comparisons.

### Bulk RNA-seq analysis in mouse tissues

#### Whole transcriptome analysis

For RNA-sequencing library preparation, RNA was extracted from mouse lungs, livers, and spleens using RNA Plus Mini Kit (Qiagen) according to manufacturer’s instructions. The concentration and integrity of total RNA were analyzed using electrophoretic separation on microfabricated chips in Agilent 2100 Bioanalyzer System (Agilent, CA, USA). After, mRNA-sequencing library preparation (1.5 µg total RNA per sample) was carried out on NovaSeq 6000 PE150 instrument (2 × 150 bp, 40 million reads per sample).

#### Data analysis

For RNA-sequencing data analysis, Fastq files were align on mm10, Mus musculus genome, by Hisat2,^77^ BAM files were counted by HTSeq-count.^78^ The obtained raw count files were analyzed with R package (R Foundation for Statistical Computing; https://www.R-project.org/) DESeq2.^79^ The genes of interest were selected based on thresholds (fold change ≥1.5 and *p* value ≤ 0.05). Heatmaps were generated with the R package ComplexHeatmap,^80^ Venn diagrams with ggVennDiagram,^81^ all other plots using ggplot2. Gene ontology was realized with gprofiler,^82^ clusterProfiler,^83^ and enrichplot.^84^

### Immunofluorescence cytofluorometric analyses

We followed a well-established protocol to generate single-cell suspensions from tissues and to perform immunofluorescence flow cytometry phenotyping of immune cells.^85^

#### Liver dissociation into single-cell suspensions

Livers were harvested, weighed, and placed in DMEM medium before mechanical disruption with the aid of scalpels and further enzymatic dissociation using the Liver Dissociation Kit (Miltenyi Biotec) and gentleMACS Octo Dissociator (Miltenyi Biotec) according to the manufacturer’s instructions. Cell suspensions were filtered through a 100 µm strainer, washed, and subjected to red blood cell lysis using RBC lysis buffer (Invitrogen, Cat. 50-112-9743) for 5–7 min at room temperature. After washing and filtering through a 70 µm strainer, cells were resuspended in PBS1X at 200 µL per 100 mg tissue for downstream flow cytometry analysis.

#### Kidney dissociation into single-cell suspensions

Kidneys were weighed, minced, and placed in RPMI medium before mechanical disruption and further enzymatic dissociation using the Multi Tissue Dissociation Kit 2 (Miltenyi Biotec) and gentleMACS Octo Dissociator (Miltenyi Biotec) according to the manufacturer’s protocol. Cell suspensions were filtered through a 100 µm strainer into 15 mL tubes, washed with PBS, and centrifuged (300 × *g*, 10 min). Red blood cells were lysed with RBC lysis buffer for 7 min at room temperature, followed by washes and centrifugation at 400 × *g*. Final cell pellets were resuspended in PBS1X at a concentration proportional to tissue weight (e.g., 200 µL per 100 mg) to proceed with flow cytometry.

#### Heart dissociation into single-cell suspensions

Mouse hearts were harvested and processed by mechanical disruption before proceeding with enzymatic dissociation using the Multi Tissue Dissociation Kit 2 (Miltenyi Biotec) and the gentleMACS Octo Dissociator, following the manufacturer’s protocol with minor modifications. Following dissociation, 7.5 mL of RPMI + 20% FBS was added, and samples were filtered through a 70 µm strainer, washed, and centrifuged (600 × *g*, 5 min). Pellets were resuspended in PBS, and 900 µL of cold Debris Removal Solution (Miltenyi Biotec, Cat. 130-109-398) was added, followed by gentle overlaid with PBS1X. After centrifugation (3000 × *g*, 10 min), debris was removed, and cells were washed and treated with RBC lysis buffer (2 min, RT). Cells were washed and resuspended in PBS1X based on tissue weight (e.g., 200 µL per 100 mg) before proceeding to flow cytometry.

#### Spleen dissociation into single-cell suspensions

Spleens were harvested and mechanically dissociated by pressing the tissue through a 70 µm strainer in RPMI medium. Cell suspensions were washed and subjected to red blood cell lysis for 7 min at room temperature, followed by washing and filtration. Cells were resuspended in PBS1X at approximately 2000 µL per 80–100 mg tissue for flow cytometry analysis.

#### Surface staining

For all samples, cell viability was assessed by incubation with LIVE/DEAD™ Fixable Yellow dye (Thermo Fisher Scientific) for 15 min at 4 °C. After PBS washing, Fc receptors were blocked with anti-mouse CD16/CD32 (clone 2.4G2, Mouse BD Fc Block™, BD Biosciences) diluted in FC buffer (0.5% BSA in PBS) for 10 min at 4 °C. After washing, pre-fixation surface staining was conducted with the following antibody panel diluted in BD brilliant buffer (50% volume) (BD Biosciences), complemented with FC buffer with 25 min incubation at 4 °C.

##### Panel profiling of tissue-infiltrating immune cells with a focus on myeloid cells

Cell surface staining was performed with the following fluorochrome-conjugated antibodies: anti-CD11c BUV395 (clone N418, BD Biosciences, Cat#744180), anti-MHC-II (I-A/I-E) BUV496 (clone M5/114.15.2, BD Biosciences, Cat#750281), anti-FcεRI BUV661 (clone MAR-1, BD Biosciences, Cat#751765), anti-CCR2 BV421 (clone SA203G11, BioLegend, Cat#150605), anti-Ly-6C BV605 (clone AL-21, BD Biosciences, Cat# 563011), anti-CD38 BV711 (clone 90/CD38, BD Biosciences, Cat# 740697), anti-CD206 BV785 (clone C068C2, BioLegend, Cat# 141729), anti-CD11b FITC (clone M1/70, BioLegend, Cat# 101206), anti-SIGLEC-F PerCP-Cy5.5 (clone E50-2440, BD Biosciences, Cat# 565526), anti-Ly-6G PE (clone 1A8, BD Biosciences, Cat# 551461), anti-F4/80 PE-Vio770 (clone REA126, Miltenyi Biotec), anti-CD45 APC-Vio770 (clone 30-F11, Miltenyi Biotec), and a lineage dump channel using APC-conjugated antibodies: anti-CD3 (clone 17A2, eBioscience Cat#17-0032-82), anti-CD19 (clone 1D3, BD Biosciences, Cat# 550992), anti-CD20 (clone SA275A11, BioLegend, Cat# 150412), and anti-NK1.1 (clone PK136, BioLegend, Cat# 108710). Cells were then fixed and permeabilized in eBioscience™ Foxp3/Transcription Factor Staining Buffer (Thermo Fisher Scientific). After washing, cells were resuspended in 400 μL of FC buffer before flow cytometer acquisition. Fully stained samples were run through a BD LSRFortessa X-20 Cell Analyzer using BD FACSDiva software (BD Biosciences).

#### Data analysis

Post-acquisition analyses were performed using Omiq.ai (https://app.omiq.ai/). Briefly, these analyses consisted of an initial supervised cell gating of compensated flow cytometry acquired data, followed by dimensionality reduction by Opt-sne and clustering by FlowSOM for selected cell markers for the antibody panel for each tissue and cell population analyzed using default settings, adding some user chosen clusters-means. The most optimal clusters-means (Elbow metaclustering for kidney and heart, and consensus metaclustering for liver) from FlowSOM clustering were used for cell count and percentage analyses. Absolute cell counts were determined after considering the proportion of the stained cell suspension run through the flow cytometer, were normalized by organ weight, and used to calculate cell counts and percentages. Statistical comparisons were conducted using two-way ANOVA test followed by pairwise comparisons.

### Metabolomics analysis

#### Sample processing

First, 30 mg of liver, spleen, kidney, and heart tissue were weighed and transferred into 2 mL homogenizer tubes containing ceramic beads (Hard Tissue Homogenizing CK28, 2.8 mm zirconium oxide beads; Precellys, Bertin Technologies). Each tube was filled with 1 mL of ice-cold extraction solvent (methanol/water, 9:1, −20 °C) containing a cocktail of internal standards. To ensure thorough extraction of endogenous metabolites, tissues were homogenized using a Precellys 24 tissue homogenizer (3 cycles of 20 s at 5000 rpm). Homogenates were centrifuged at 15,000 × *g* for 10 min at 4 °C, and the resulting supernatants were collected. For plasma samples, 25 µL of plasma was mixed with 250 µL of the same ice-cold extraction solvent. After vortexing to facilitate protein precipitation and metabolite extraction, samples were centrifuged under the same conditions (15,000 × *g*, 10 min, 4 °C). Supernatants from both tissue and plasma samples were collected and divided into three fractions for downstream metabolomics workflows, following protocols previously described.^11^

#### Data analysis

Metabolomics data analysis was conducted with the R package (R Foundation for Statistical Computing; https://www.R-project.org/) MetaboDiff.^86^ Significant metabolites were select based on differences in means (dm), with the thresholds abs(dm) ≥0.5 and *p* value ≤ 0.05 (t-test), and depicted as ggVennDiagram.^81^ Heatmaps were realized with ComplexHeatmap.^80^ Pathway enrichment analysis was performed using the enrichment module of MetaboAnalyst (https://www.metaboanalyst.ca/). Enrichment testing employed the *globaltest* algorithm, which models associations between metabolite sets and the outcome using a generalized linear model. For each set, a Q-statistic is computed as the mean of squared covariances (Q values) between individual metabolites and the outcome. Metabolite sets were drawn from a library of 3694 pathways based on RaMP-DB (integrating KEGG, HMDB, Reactome, WikiPathways).

#### Partial least squares discriminant analysis (PLS-DA) of metabolomic profiles

Metabolite peak areas obtained from MS analysis were log-transformed, mean-centered, and scaled separately for each organ. The resulting datasets were subjected to PLS-DA, designed to maximize separation between control (sham/PBS + IgG) and disease (CLP/PBS + IgG) groups. All samples were then projected into the same PLS-DA space, and their scores along the primary component were extracted. For each sample, a displacement from the control group median was computed along this axis. These displacements were normalized between 0 (disease group median) and 1 (control group median), yielding a reversion score that allowed ranking of treatment groups according to their degree of metabolic recovery.

#### Enzyme-metabolite correlations

Pairwise comparisons identified metabolites altered during CLP-induced sepsis (*p* < 0.05, |dm| ≥ 0.3). Heatmaps display dm values (red: increased; blue: decreased). Metabolites altered in all comparisons were used for enzyme-metabolite analysis. Metabolites were mapped to pathways via KEGG, HMDB, and Reactome. Enzymes producing or consuming these metabolites, including those acting on immediate precursors/products, were identified using KEGG, UniProtKB, and Reactome. Only enzymes detected in liver RNA-seq across CLP conditions were retained. Significantly altered enzymes were visualized as log2-fold change in heatmaps. Expected enzyme-metabolite correlations were assigned based on biochemical roles: synthesizers and transporters with positive correlation, degraders and competitors with negative correlation.

### Statistical analyses

Continuous variables were described as mean ± standard error of the mean (SEM), except Fig. 1b, c, h–j, where data are presented as box-and-whisker plots showing the median, interquartile range, and whiskers extending from minimum to maximum values. Individual data points are overlaid. Prior to analysis, the normality of data distribution was evaluated using the D’Agostino–Pearson, Shapiro–Wilk, and Kolmogorov–Smirnov tests. For group comparisons, normally distributed data were analyzed using unpaired two-tailed Student’s t-tests for two groups, and one-way or two-way ANOVA followed by Tukey’s or Sidak’s post hoc tests for multiple groups. Pairwise t-tests with FDR correction were applied where appropriate. Non-normally distributed data were analyzed using the Wilcoxon signed-rank test for two-group comparisons, and the Kruskal–Wallis test for comparisons across multiple groups. Spearman correlation matrices were calculated using the rcorr() function from the Hmisc package (v5.1-3), clustered using hclust() from the stats package (v4.3.3), and visualized using the corrplot package (v0.94). A multivariate analysis of variance was conducted on the first three principal components analysis to assess group differences, followed by pairwise comparisons using t-tests with FDR correction. Survival analyses were performed using the Kaplan–Meier method, and differences in survival were assessed with the log-rank test. Survival curves were fitted using survfit() from the survival package (v3.5-5), stratified by median hACBP levels, and visualized with ggsurvplot() from the survminer package (v0.5.0), including survival probabilities, *p* values, and risk tables. Receiver operating characteristic (ROC) curves were generated using the pROC package (v1.18.5) to assess the predictive performance of biomarkers and scoring systems, and area under the curve (AUC) was calculated using DeLong’s method. Heatmaps were plotted using the ComplexHeatmap package (v2.16.0). Venn diagram analyses were performed to assess overlap between differentially expressed genes and metabolites based on significance thresholds (adjusted *p* < 0.05) using the VennDiagram package (v1.7.3). Summary supplementary tables were generated by computing the mean and SEM for each numeric variable within groups. Group comparisons were conducted using either the Kruskal–Wallis test or one-way ANOVA, depending on data distribution. Categorical variables were evaluated using Fisher’s exact test with simulated *p* values. Pairwise comparisons between groups were performed using the Wilcoxon rank-sum test. Missing or insufficient data were left blank. Data were processed using the dplyr, tidyr, rstatix, and gtsummary packages in R, and tables were formatted with flextable. Flow cytometry data were analyzed by manual gating using either the omiq.ai platform or FlowJo software (v10.6.1), and population frequencies and marker intensities were compared using two-way ANOVA with post hoc comparisons. All statistical analyses were performed using R software version 4.4.1 (2024-06-14 ucrt) (R Foundation for Statistical Computing; https://www.R-project.org/) and GraphPad Prism v10, unless otherwise specified. A *p* value or FDR-adjusted *p* value < 0.05 was considered statistically significant.

## Supplementary information

Supplementary Material

## Data Availability

All the raw data are added as supplementary tables or on appropriate public repositories. The sequencing data reported in this paper have been deposited in the NCBI GEO database under accession numbers GSE313149, GSE304860, and GSE304861 (URL: https://www.ncbi.nlm.nih.gov/geo/) and are publicly available. The metabolomics data reported in this paper have been deposited in Metabolomics Workbench with accession numbers ST004445, ST004592, ST004593, ST004594, and ST004595 (URL: https://www.metabolomicsworkbench.org/). Requests for further information and resources should be directed to and will be fulfilled by the lead contact, G.K.
