# Supplementary material for: Acyl-CoA-binding protein (ACBP): a poor-prognosis biomarker in sepsis and a target for disease mitigation
Source: Signal Transduct Target Ther. 2026 Apr 2;11:119. doi: 10.1038/s41392-026-02670-z (PMC13046771; doi:10.1038/s41392-026-02670-z)
Supplement: Supplementary file 1 — Supplementary Material [file 41392_2026_2670_MOESM1_ESM.docx]

Supplementary Materials for

**Acyl-CoA-binding protein (ACBP): a poor-prognosis biomarker in sepsis and a target for disease mitigation**

Flavia Lambertucci, Omar Motiño, Uxía Nogueira-Recalde, Yan Rong, Léa Montégut, María Pérez-Lanzón, Vincent Carbonnier, Sijing Li, Sylvère Durand, Fanny Aprahamian, Hui Chen, Yanbing Dong, Allan Sauvat, Silvia Mingoia, Sylvie Lachkar, Ester Saavedra, Jonathan Pol, Federico Pietrocola, Maria Chiara Maiuri, Estela Rocha-Oliveira, Roberto Roncon-Albuquerque Jr, Francisco Vasques-Nóvoa, Roberto Lozano-Rodríguez, José Avendaño-Ortiz, Eduardo López-Collazo, Mahmoud Abdellatif, Isabelle Martins and Guido Kroemer

Correspondence to: [**isabelle.martins@inserm.fr**](mailto:isabelle.martins@inserm.fr) (I.M.), [**kroemer@orange.fr**](mailto:kroemer@orange.fr) (G.K.)

**This PDF file includes:**

Figures. S1 to S13

Tables S1 to S6

**Supplementary Figure S1**

**
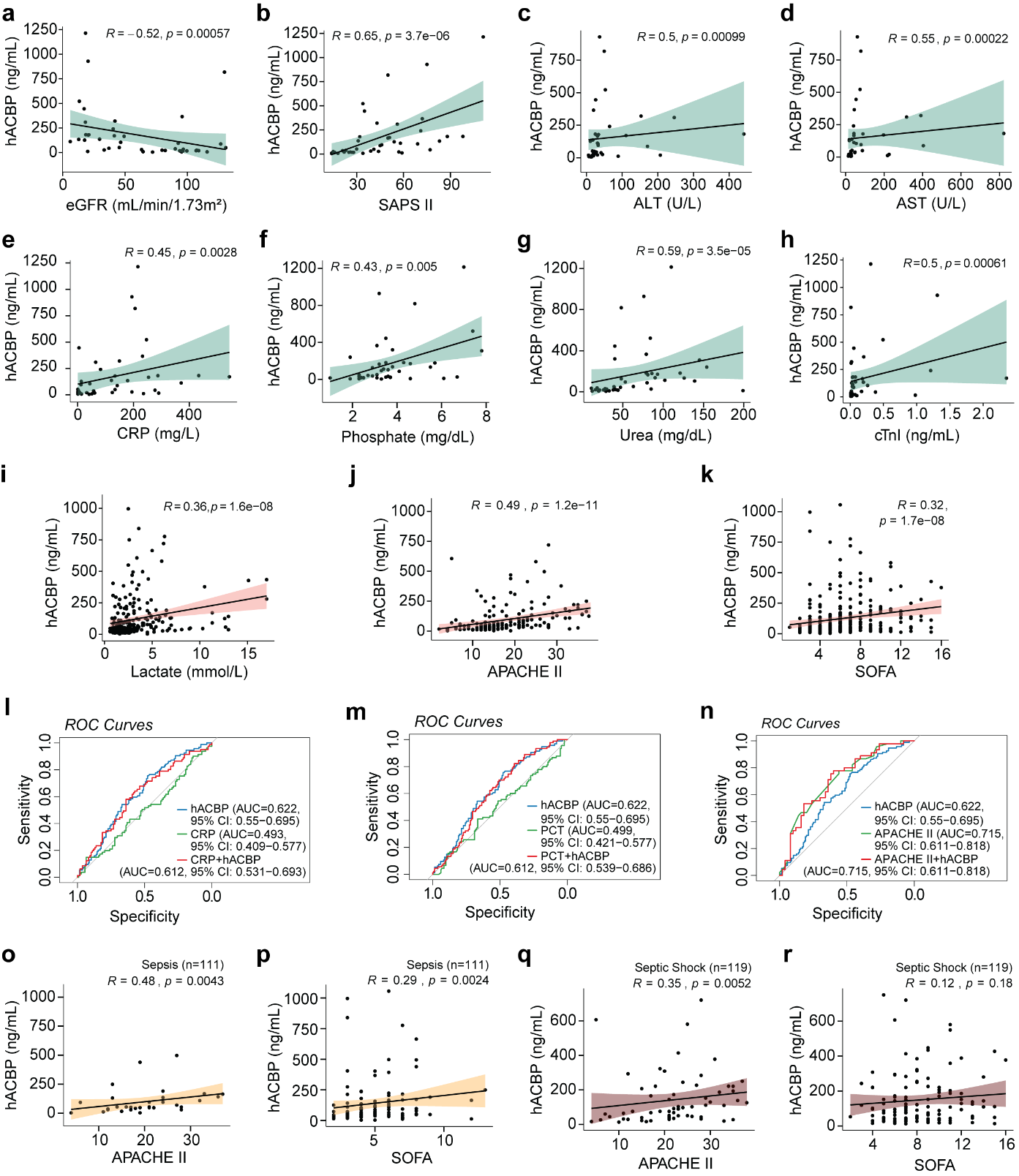
**

**Supplementary Figure S1. Human ACBP/DBI plasma levels correlate with clinical prognosis indicators in sepsis.** Plasma levels of human ACBP (hACBP) **(a)** negatively correlate with eGFR, a marker of renal function, and positively correlate with **(b)** SAPS II, **(c)** ALT, **(d)** AST, **(e)** CRP, **(f)** phosphate, **(g)** urea, and **(h)** cTnI in patients from the first cohort (n = 43). **(i-k)** Positive correlation of hACBP with **(i)** lactate, **(j)** APACHE II score and **(k)** SOFA score in all septic patients (n = 230). **(l-n)** Receiver operating characteristic (ROC) curves showing the predictive performance of plasma hACBP, **(l)** CRP, **(m)** PCT or **(n)** APACHE II scores and their combination with hACBP, for identifying mortality in septic patients from a second cohort (n = 230). **(o, p)** In sepsis, hACBP plasma levels positively correlate with **(o)** APACHE II and **(p)** SOFA scores (n = 111). **(q, r)** In septic shock patients, hACBP continues to correlate with **(q)** APACHE II but not with **(r)** SOFA score. Spearman’s correlation coefficients, and p-values were used for individual correlations. ROC curves include AUC intervals calculated using DeLong’s method. Abbreviations: eGFR: estimated glomerular filtration rate; SAPS II: Simplified Acute Physiology Score II; ALT: alanine aminotransferase; AST: aspartate aminotransferase; CRP: C-reactive protein; cTnI: cardiac troponin I; PCT: Procalcitonin; SOFA: Sequential Organ Failure Assessment; APACHE II: Acute Physiology and Chronic Health Evaluation II.

**Supplementary Figure S2**


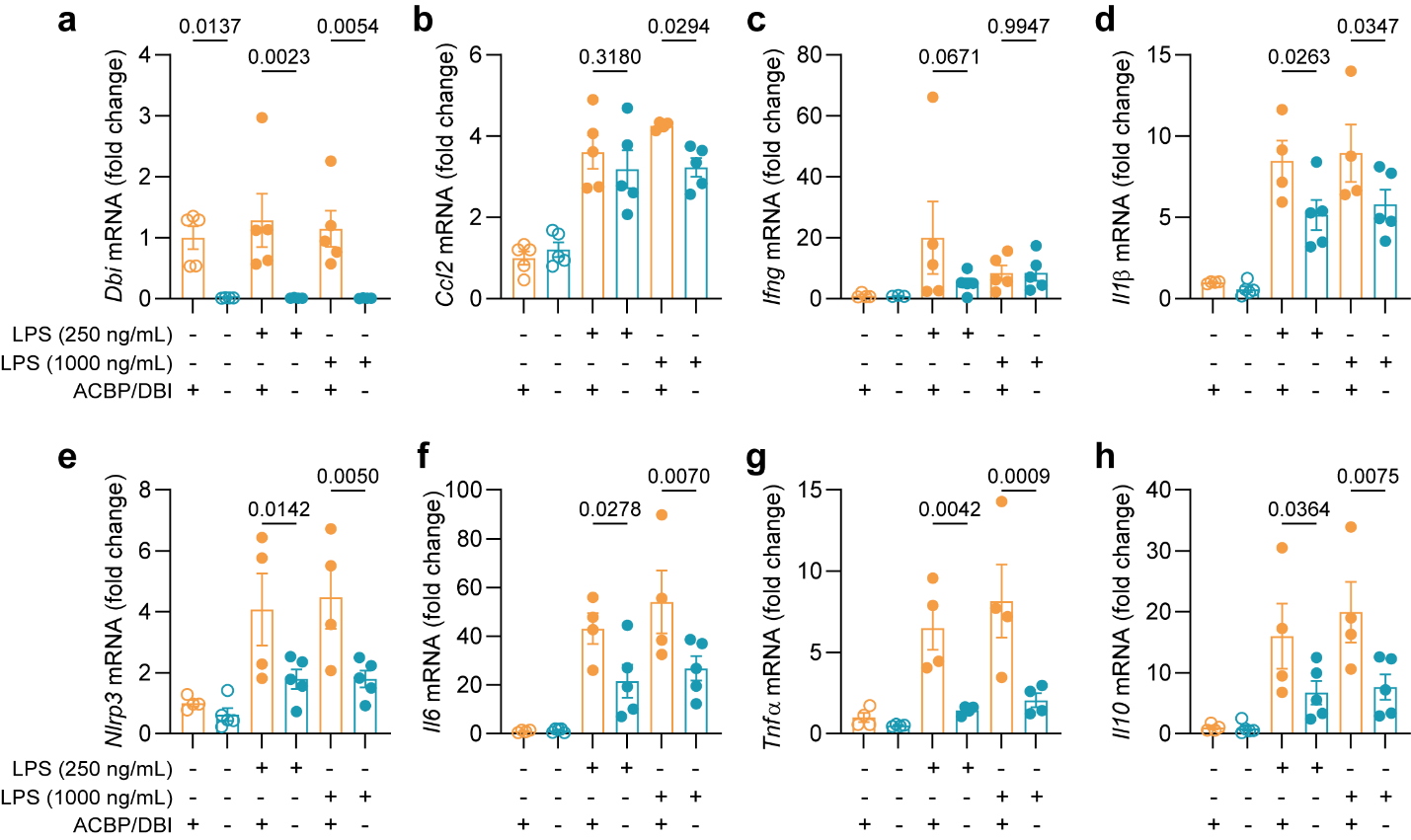


**Supplementary Figure S2. LPS-induced inflammatory gene expression is altered in hepatocytes from inducible Acbp/Dbi^-/-^ knockout mice.** Fold changes in mRNA expression measured by RT-qPCR in primary hepatocytes isolated from inducible whole-body *Acbp/Dbi* ^⁻/⁻^ or control mice treated *in vitro* with lipopolysaccharide (LPS; 250 ng/mL or 1000 ng/mL). mRNA expression levels are shown for: **(a)** *Dbi*, **(b)** *Ccl2*, **(c)** *Ifng*, **(d)** *Il1b*, **(e)** *Nlrp3*, **(f)** *Il6*, **(g)** *Tnf*, and **(h)** *Il10*. Data are expressed as means ± SEM (n=5 mice per group). Statistical comparisons were performed using one-way ANOVA followed by estimation of marginal means for pairwise comparisons.

**Supplementary Figure S3**

**
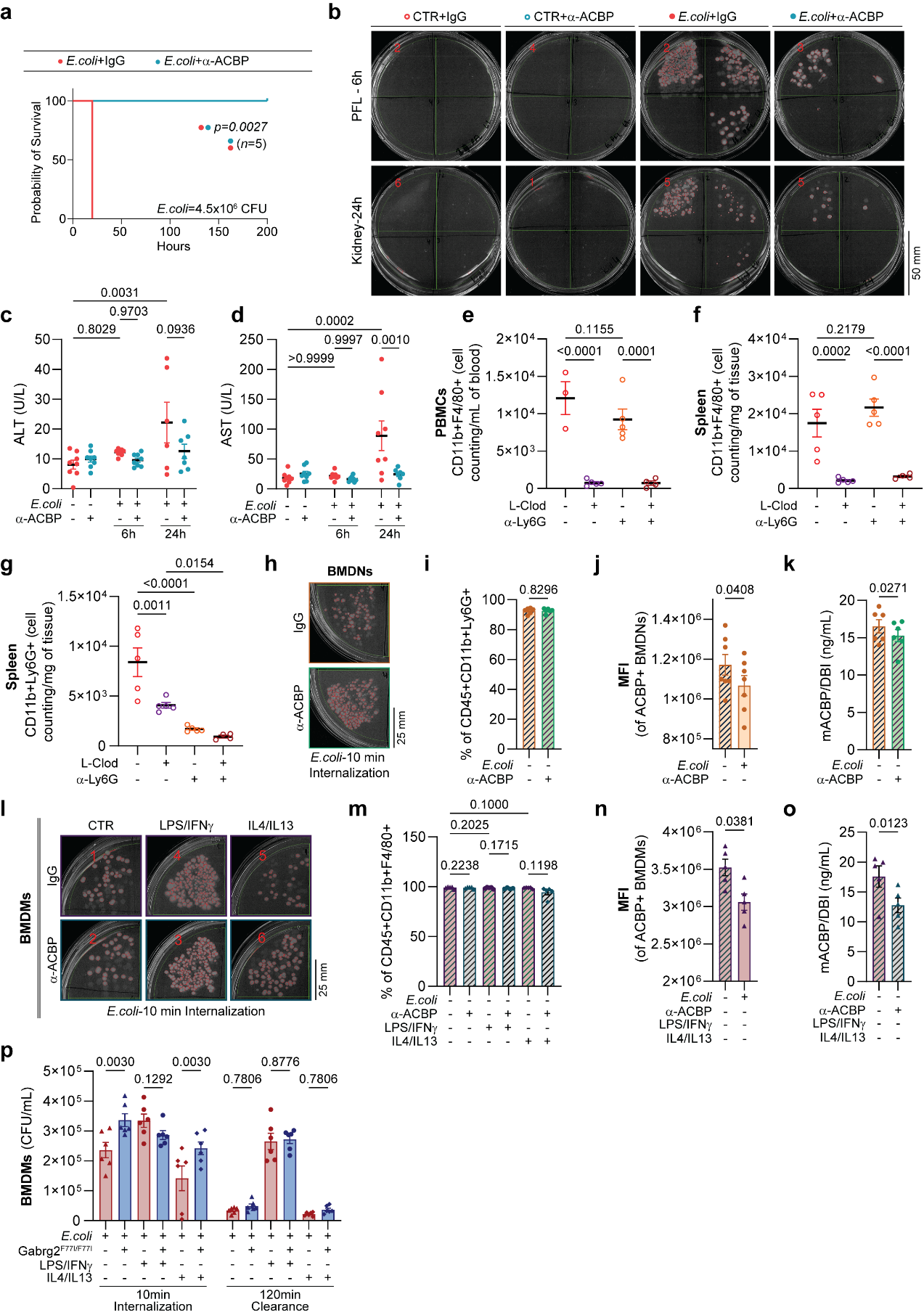
**

**Supplementary Figure S3. α-ACBP treatment enhances bacterial clearance *in vivo* and *in vitro*. (a)** Survival curve of mice pretreated with weekly administration of α-ACBP (5 mg/kg body weight, i.p.) the week prior to *E. coli* challenge, followed by two additional doses (2.5 mg/kg, i.p.) at 4 h and 1 h before intraperitoneal injection of *E. coli* (4.5 × 10⁶ CFU/mouse). **(b)** Representative images of *E. coli* colonies grown on LB agar plates from PLF and kidneys of mice treated with or without α-ACBP. Automated detection of colonies is shown as red overlay. **(c, d)** Plasma levels of ALT and AST were measured at 6 h and 24 h post-infection (n=4-8 mice per group). **(e–g)** Twelve-week-old male C57BL/6J mice were pretreated with clodronate liposomes (100 µL/10 g body weight, i.v.) or control liposomes 48 h prior, and with anti-mouse Ly6G (clone 1A8, 50 µg/mouse, i.p.) or IgG2a isotype control at 36 h and 12 h prior to sample collection (n=3-5 mice per group). Flow cytometry was used to quantify: **(e)** CD11b⁺F4/80⁺ cells in PBMCs, **(f)** CD11b⁺F4/80⁺ cells in spleen, and **(g)** CD11b⁺Ly6G⁺ cells in spleen. **(h)** Representative LB agar images showing *E. coli* CFUs from internalization assays in bone marrow-derived neutrophils (BMDNs) treated with or without α-ACBP. Automated detection of colonies is shown as red overlay. **(i)** Flow cytometry was used to quantify % of CD45^+^CD11b^+^Ly6G^+^ cells in BMDNs treated with α-ACBP for 16 hours (n=5-7 mice per group). **(j)** MFI in CD45^+^CD11b^+^Ly6G^+^ACBP^+^ cells showing ACBP released in BMDNs after 10 min exposure to *E. coli* (MOI=10). **(k)** Plasma mACBP/DBI levels measured by ELISA in the supernatant of BMDNs treated with α-ACBP for 16 hours. **(l)** Representative LB agar images showing *E. coli* CFUs from internalization assays in bone marrow-derived macrophages (BMDMs) treated with or without α-ACBP. Automated detection of colonies is shown as red overlay. **(m)** Flow cytometry was used to quantify % of CD45^+^CD11b^+^F4/80^+^ cells in BMDMs treated with α-ACBP for 16 hours (n=5 mice per group). **(n)** MFI in CD45^+^CD11b^+^F4/80^+^ACBP^+^ cells showing ACBP released in BMDMs after 10 min exposure to *E. coli* (MOI=10). **(o)** Plasma mACBP/DBI levels measured by ELISA in the supernatant of BMDMs treated with α-ACBP for 16 hours. **(p)** BMDMs from male C57BL/6J and Gabrg2^F77I/F77I^ mice were differentiated over 7 days (≥70–80% F4/80⁺), then stimulated with LPS (100 ng/mL) + IFNγ (25 ng/mL) or IL-4 (25 ng/mL) + IL-13 (25 ng/mL) for 24 h (n=6 mice per group). Killing assays were performed with *E. coli* (MOI = 10), and CFU/mL were determined after overnight incubation. Results are presented as means ± SEM. Statistical comparisons were performed using one-way or two-way ANOVA with estimation of marginal means for pairwise comparisons, or Student’s t-test where appropriate. Abbreviations: IFNγ: Interferon-γ; IL-4: Interleukin-4; IL-13: Interleukin-13; BMDNs: bone marrow-derived neutrophils; BMDMs: bone marrow-derived macrophages; CFU: colony-forming unit; PBMCs: peripheral blood mononuclear cells; ALT: alanine aminotransferase; AST: aspartate aminotransferase; MFI: median fluorescence intensity.

**Supplementary Figure S4**


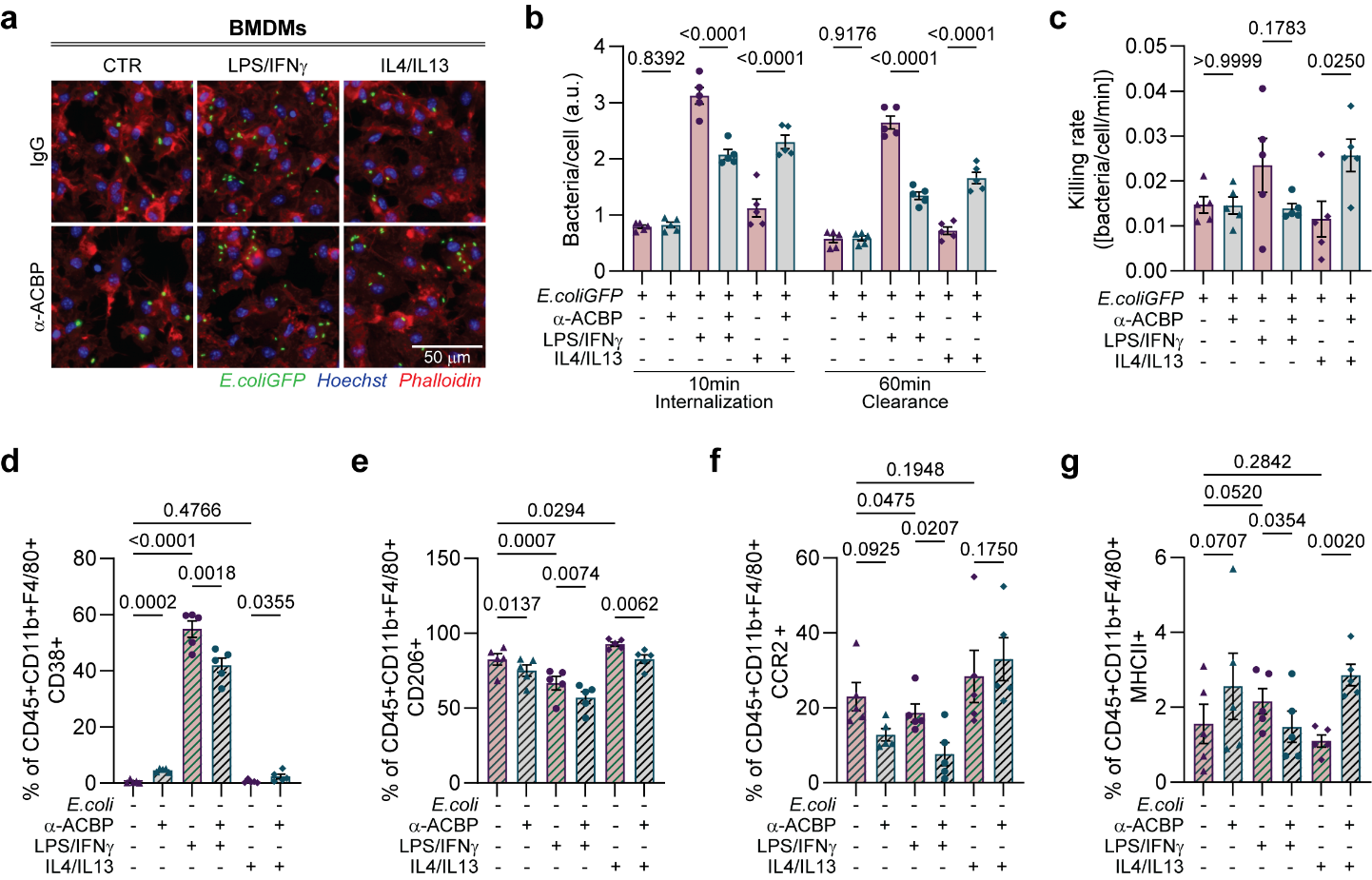


**Supplementary Figure S4. Bacterial clearance and immunophenotyping of bone marrow-derived macrophages (BMDMs) treated with or without α-ACBP. (a)** Representative confocal microscopy images of GFP-labelled *E. coli* internalized by BMDMs 10 minutes after infection. Scale bars: 50 µm. **(b)** Quantification of intracellular bacteria per cell was performed based on confocal images after 10 minutes of internalization, and after 60 minutes of clearance. **(c)** Killing rate of *E. coli*-GFP in BMDMs, calculated as intracellular bacterial load per minute following 20 minutes of internalization. **(d-g)** Flow cytometry was used to quantify: **(d)** % of CD45^+^CD11b^+^F4/80^+^CD38^+^ cells, **(e)** % of CD45^+^CD11b^+^F4/80^+^CD206^+^ cells, **(f)** % of CD45^+^CD11b^+^F4/80^+^CCR2^+^ cells, and **(g)** % of CD45^+^CD11b^+^Ly6G^+^MHCII^+^ cells in BMDMs treated with α-ACBP for 16 hours. Results are presented as means ± SEM (n=5 mice per group). Statistical comparisons were performed using one-way or two-way ANOVA with estimation of marginal means for pairwise comparisons. Abbreviations: IFNγ: Interferon-γ; IL-4: Interleukin-4; IL-13: Interleukin-13; BMDNs: bone marrow-derived neutrophils; BMDMs: bone marrow-derived macrophages; GPF: green fluorescent protein.

**Supplementary Figure S5**

–
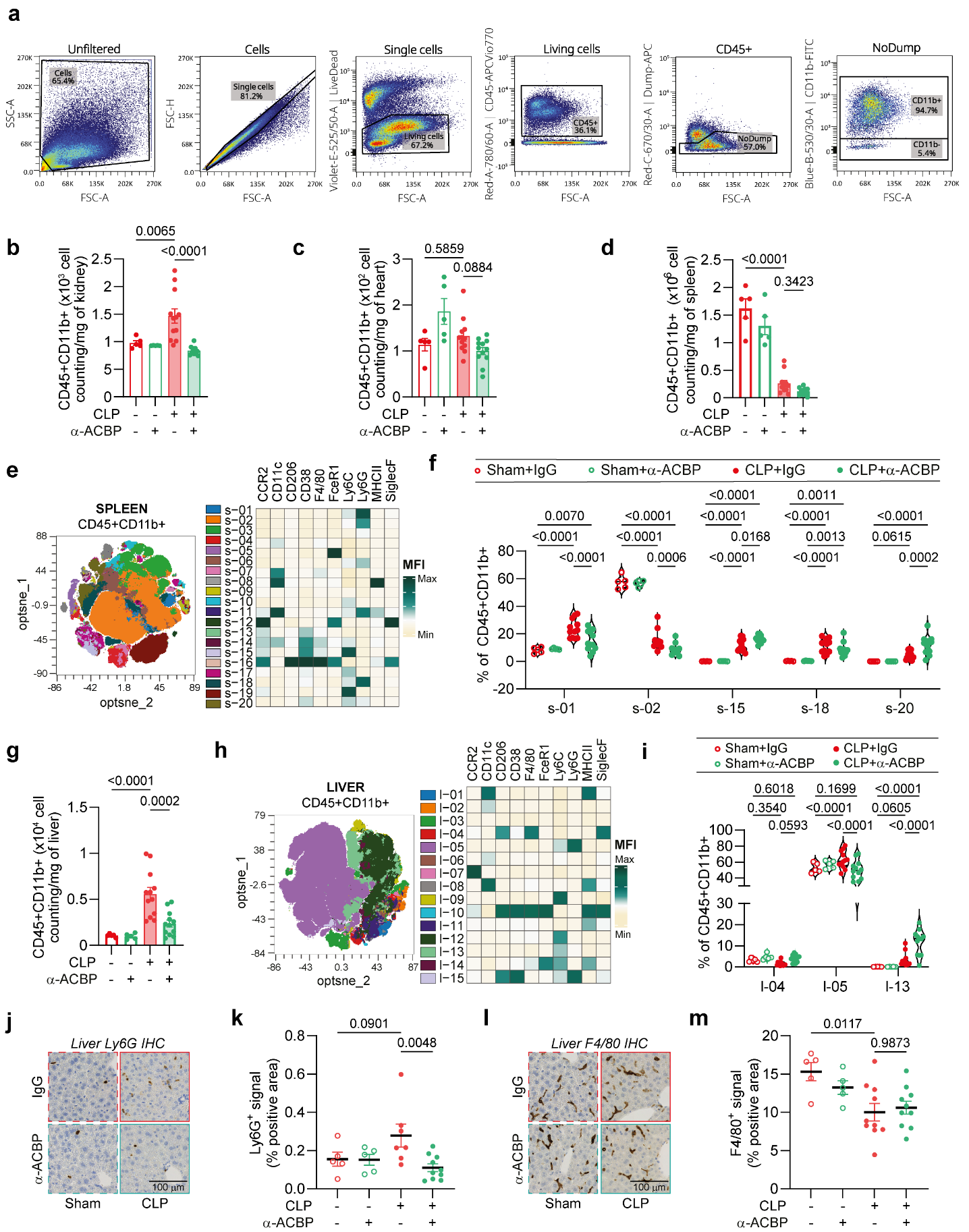


**Supplementary Figure S5. ACBP/DBI neutralization protects against CLP-induced splenic and hepatic inflammation *in vivo*. (a)** Flow cytometry analysis was conducted 24 h after CLP to identify live CD45⁺Dump⁻CD11b⁺ immune cells in kidney, heart, and liver tissues (n=5-12 mice per group). The dump channel (APC) included antibodies against CD3, CD19, CD20, and NK1.1. A representative gating strategy from the heart is shown. **(b-d)** Quantification of CD45⁺CD11b⁺ immune cells per mg of tissue in **(b)** kidney, **(c)** heart and **(d)** spleen. **(e)** In the spleen, 20 immune cell populations within the CD45⁺CD11b⁺ compartment were defined, and their median fluorescence intensities (MFI) for each marker are shown in a heatmap. **(f)** Relative abundance of selected splenic immune cell clusters (those significantly altered by α-ACBP treatment), expressed as a percentage of CD45⁺CD11b⁺ cells. **(g)** Total CD45⁺CD11b⁺ cell counts per mg of liver tissue. **(h)** In the liver, 15 immune cell populations within the CD45⁺CD11b⁺ compartment were defined, and their MFIs for each marker are shown in a heatmap. **(i)** Relative abundance of selected immune cell clusters (those significantly altered by α-ACBP treatment), expressed as a percentage of CD45⁺CD11b⁺ cells. **(j, l)** Representative immunohistochemistry (IHC) images of **(j)** Ly6G⁺ and **(l)** F4/80⁺ staining in liver sections from sham or CLP mice treated with α-ACBP or isotype control (n=5-10 mice per group). **(k, m)** Quantification of **(k)** Ly6G⁺ and **(m)** F4/80⁺ positive signals in liver sections. Data are presented as mean ± SEM. Comparisons between groups were performed using one-way ANOVA followed by estimation of marginal means for pairwise comparisons. Immune population analyses were conducted using two-way ANOVA followed by pairwise comparisons. Compensation, scaling, and gating strategies were implemented using the omiq.ai platform.

**Supplementary Figure S6**


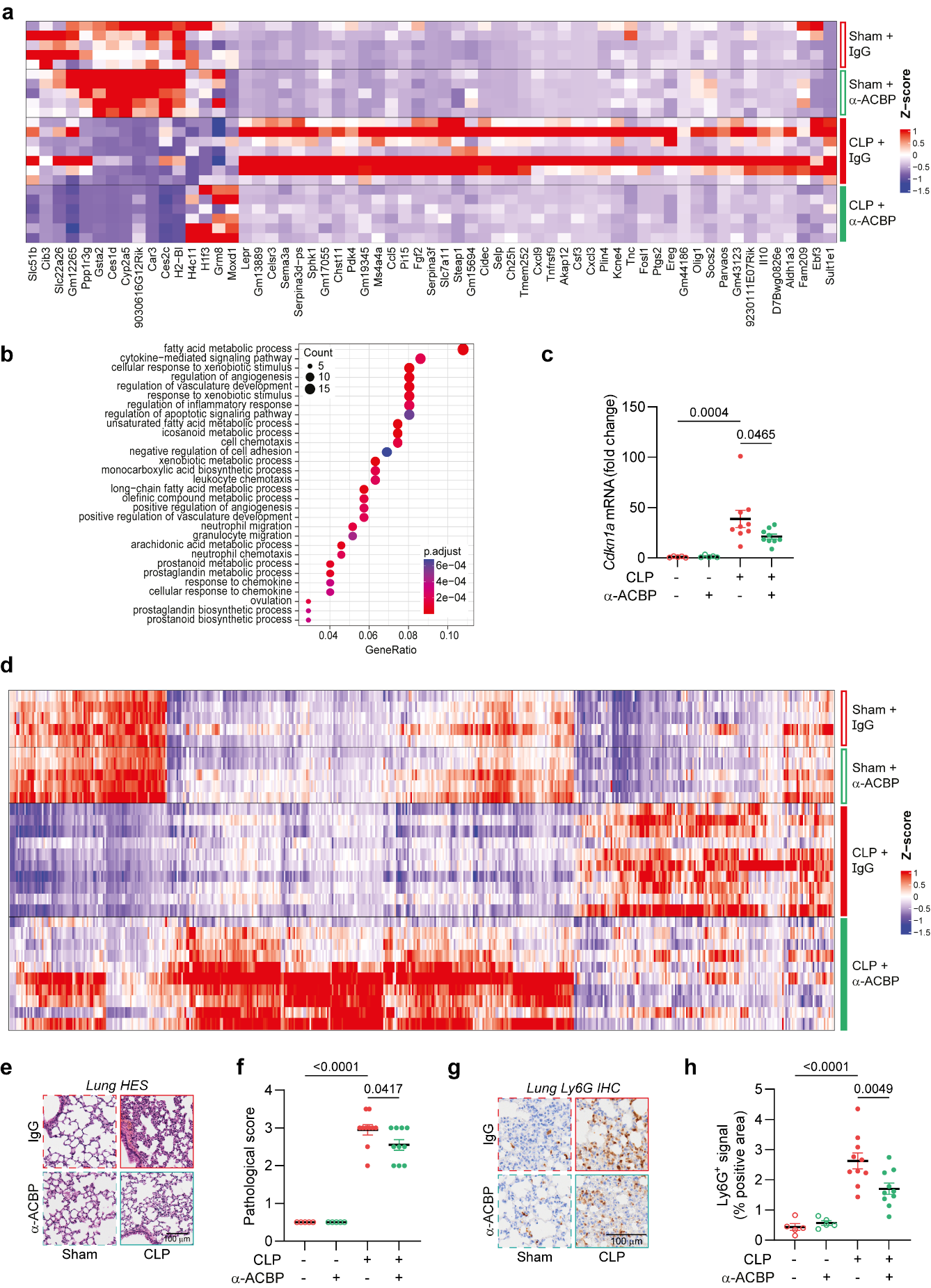


**Supplementary Figure S6. Neutralization of ACBP/DBI restores CLP-induced tissue damage and reverses transcriptomic alterations in the liver and lung. (a)** Heatmap clustered by Euclidean distance showing changes in liver mRNA expression (depicted as Z-scores) from mice treated with IgG or α-ACBP following 24h post-CLP or sham surgery (n=5-9 mice per group). **(b)** Pathway enrichment analysis of downregulated hepatic genes by α-ACBP in CLP mice was performed using Gene Ontology (GO) biological process terms. **(c)** Fold changes in liver mRNA expression of *Cdkn1a* gene extracted from **(a)**. **(d)** Heatmap clustered by Euclidean distance showing changes in lung mRNA expression (depicted as Z-scores) from mice treated with IgG or α-ACBP following 24h post-CLP or sham surgery (n=5-10 mice per group). **(e)** Representative Hematoxylin-Eosin-Safranin (HES) stained lung sections showing tissue damage in CLP mice. **(f)** Quantification of lung injury score (scale 0–6), categorized into three levels of oedema and three levels of immune cell infiltration: 0 for no oedema and infiltration, 1 for mild oedema and infiltration, 2 for moderate oedema and infiltration, and 3 for severe oedema and infiltration. **(g)** Representative immunohistochemistry (IHC) images showing Ly6G+ neutrophils infiltration in lungs of CLP mice treated with α-ACBP or isotype. **(h)** Quantification of Ly6G⁺ IHC signal indicating increased neutrophils presence in CLP lungs, which is reduced by α-ACBP treatment. Data are presented as mean ± SEM. Comparisons between groups were performed using one-way ANOVA followed by estimation of marginal means for pairwise comparisons.

**Supplementary Figure S7**


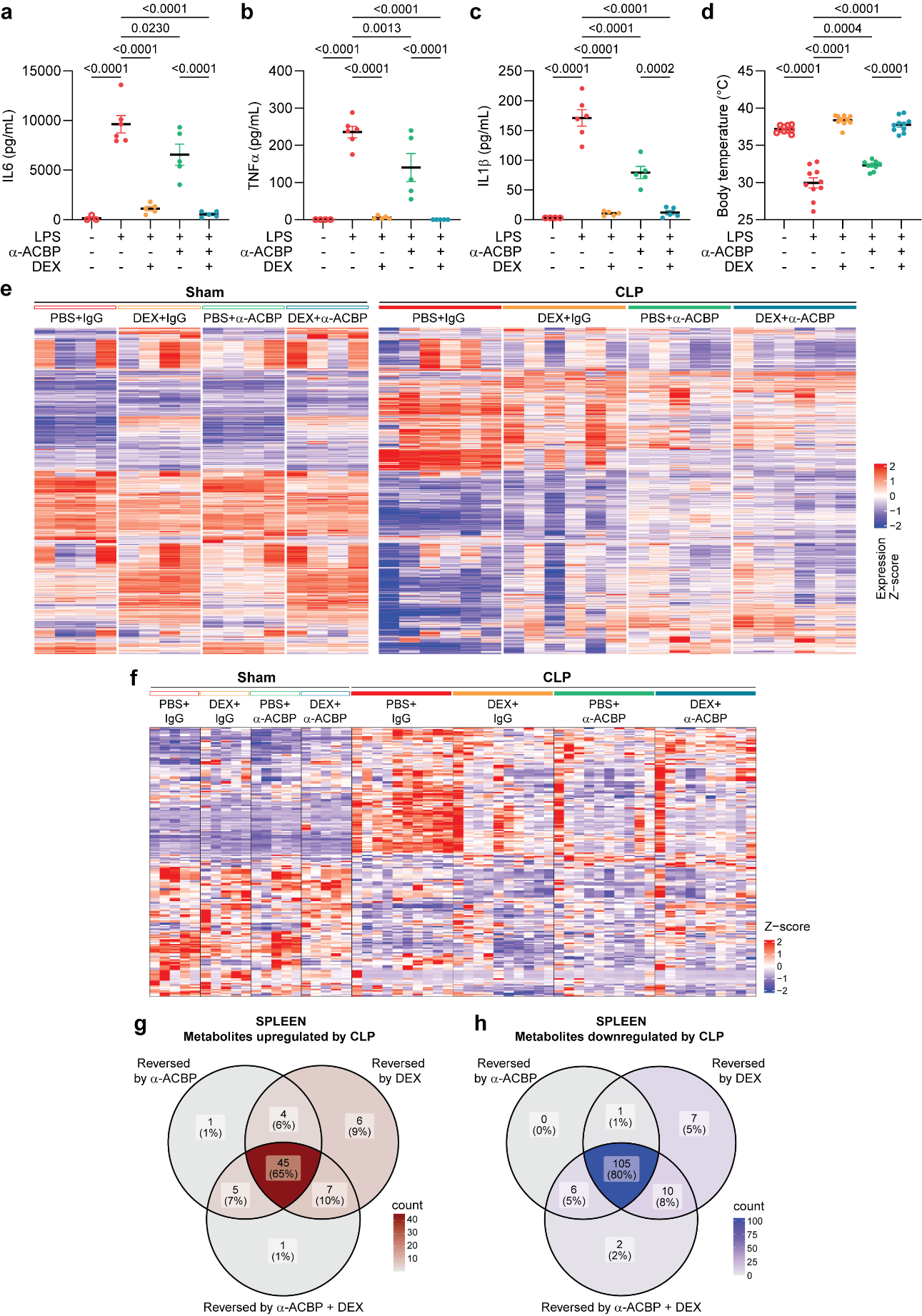


**Supplementary Figure S7. Effects of α-ACBP and/or dexamethasone on plasma cytokines and splenic metabolomes in models of sepsis. (a–c)** Plasma levels of IL-6, TNFα, and IL-1β were quantified by ELISA 6 hours after LPS injection. **(d)** Body temperature was measured at the same time point (n=3-10 mice per group). **(e)** Heatmap showing changes in spleen mRNA expression, clustered by Euclidean distance and represented as Z-scores. Samples were collected from C57BL/6J mice treated with IgG/α-ACBP, PBS/DEX, or both treatments, and subjected to either sham or CLP surgery (n=4-10 mice per group). **(f)** Heatmap showing changes in spleen metabolite concentrations, also clustered by Euclidean distance and depicted as Z-scores under the same treatment and surgical conditions. **(g, h)** Venn diagrams representing the overlap of differentially expressed metabolites in spleens of CLP mice. **(g)** Upregulated and **(h)** downregulated metabolites reversed by α-ACBP, DEX, or the combination. Data are shown as mean ± SEM. Statistical comparisons were performed using one-way ANOVA followed by estimation of marginal means for pairwise comparisons.

**Supplementary Figure S8**


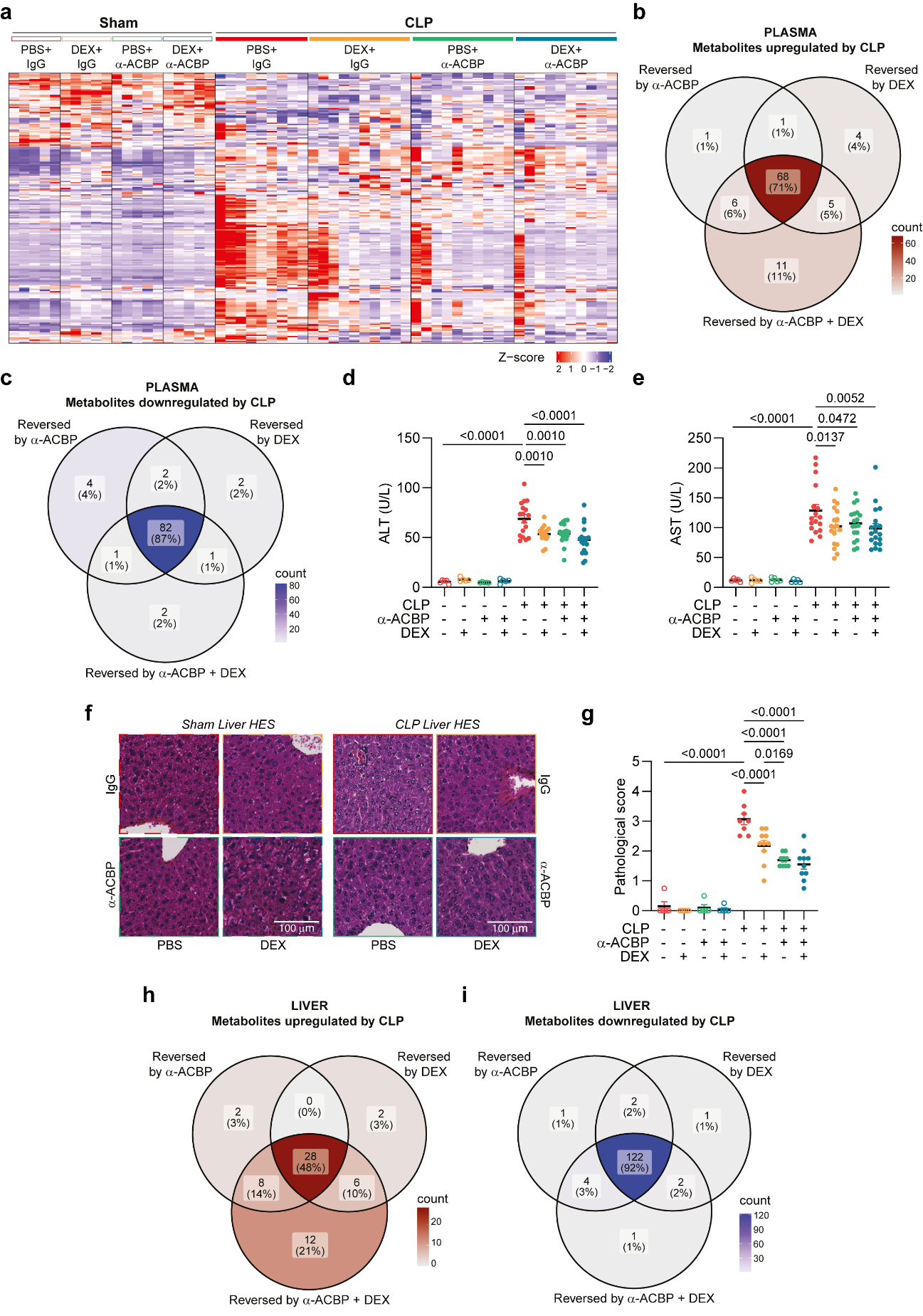


**Supplementary Figure S8. Treatment with α-ACBP and/or DEX triggers metabolic alterations in plasma and liver, along with improvements in liver function markers. (a)** Heatmap clustered by Euclidean distance showing changes in plasma metabolite concentrations, depicted as Z-scores, in sham or CLP mice treated with α-ACBP, DEX, or their combination (n=5-10 mice per group). **(b, c)** Venn diagrams showing **(b)** upregulated and **(c)** downregulated plasma metabolites from **(a)**. Plasma **(d)** ALT and **(e)** AST levels measured 24 h post-CLP (n=5-20 mice per group). **(f)** Representative HES-stained liver sections from sham or CLP mice treated with α-ACBP, DEX, or their combination. **(g)** Liver pathology scores assessed on HES-stained sections using a 0–4 grading scale: 0, no necrotic infiltrates; 1, small foci of necrotic cells; 2, foci with ~100 necrotic cells or involvement of ~30 hepatocytes; 3, necrosis affecting 10% of the section; 4, necrosis affecting ≥30% of the section. Scores were averaged from two independent examiners. **(h, i)** Venn diagrams showing **(h)** upregulated and **(i)** downregulated liver metabolites in CLP mice at 24 h, classified according to reversal by α-ACBP, DEX, or the combination treatment. Data are presented as mean ± SEM. Statistical comparisons were performed using two-way ANOVA followed by pairwise comparisons.

**Supplementary Figure S9**


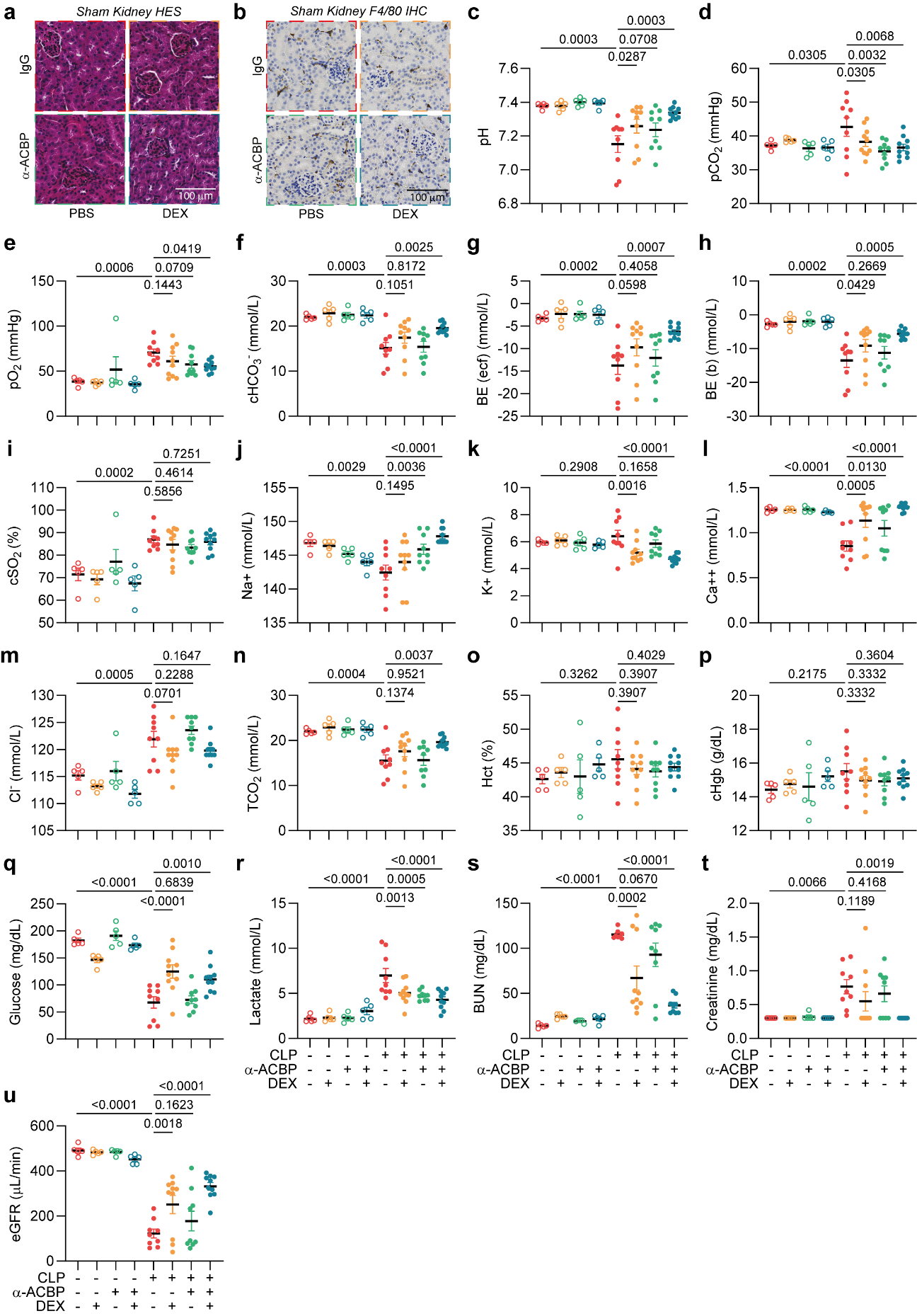


**Supplementary Figure S9. Individual renal parameters measured 24 hours after CLP surgery in mice treated with α-ACBP, DEX, or the combination. (a)** Representative HES-stained kidney sections from sham mice. **(b)** Representative immunohistochemistry (IHC) images showing F4/80⁺ signal in kidneys from sham mice treated with α-ACBP, DEX, or the combination. Renal parameters measured by the EPOC analysis system at 24 h post-surgery include: **(c)** pH, **(d)** partial pressure of carbon dioxide (pCO₂), **(e)** partial pressure of oxygen (pO₂), **(f)** calculated bicarbonate concentration (cHCO₃⁻), **(g)** base excess in extracellular fluid (BE [ecf]), **(h)** base excess in whole blood (BE [b]), **(i)** calculated oxygen saturation (cSO₂), **(j)** sodium (Na⁺), **(k)** potassium (K⁺), **(l)** calcium (Ca⁺⁺), **(m)** chloride (Cl⁻), **(n)** total carbon dioxide (TCO₂), **(o)** hematocrit (Hct), **(p)** calculated hemoglobin (cHgb), **(q)** glucose, **(r)** lactate, **(s)** blood urea nitrogen (BUN), **(t)** creatinine, and **(u)** estimated glomerular filtration rate (eGFR). Data are presented as means ± SEM (n=5-10 mice per group). Statistical analysis was performed using two-way ANOVA followed by estimation of marginal means for pairwise comparisons with FDR correction.

**Supplementary Figure S10**


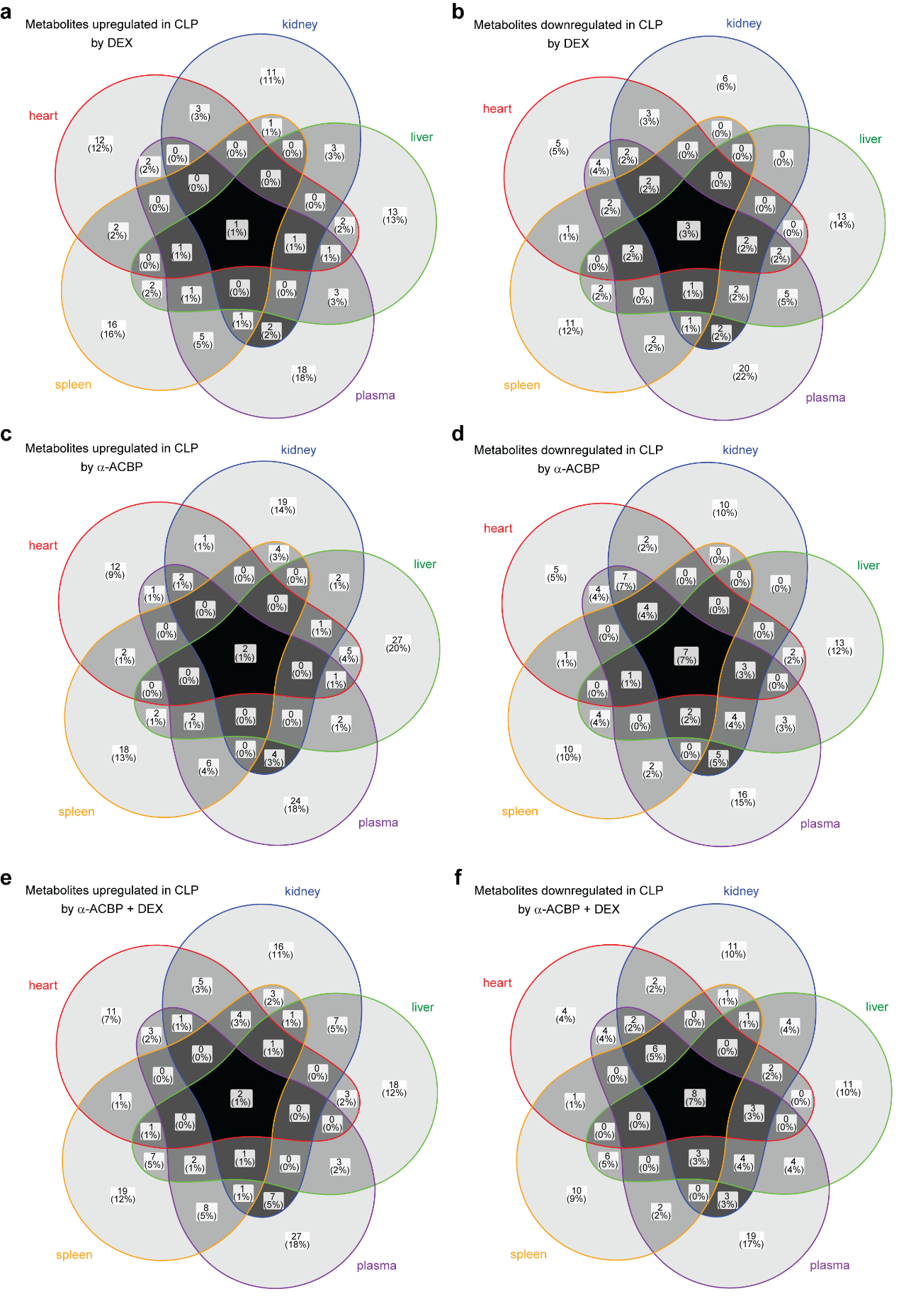


**Supplementary Figure S10. Comparative metabolomic analysis across organs following CLP and combination treatments.** Venn diagrams depict metabolites measured in the heart, kidney, liver, plasma, and spleen that were altered by CLP and subsequently corrected by α-ACBP, dexamethasone (DEX), or their combination. **(a, b)** Plasma metabolites **(a)** upregulated or **(b)** downregulated by CLP and reversed by DEX. **(c, d)** Metabolites **(c)** upregulated or **(d)** downregulated in CLP mice and corrected by α-ACBP. **(e, f)** Metabolites **(e)** upregulated or **(f)** downregulated in CLP mice and restored by the combination treatment.

**Supplementary Figure S11**


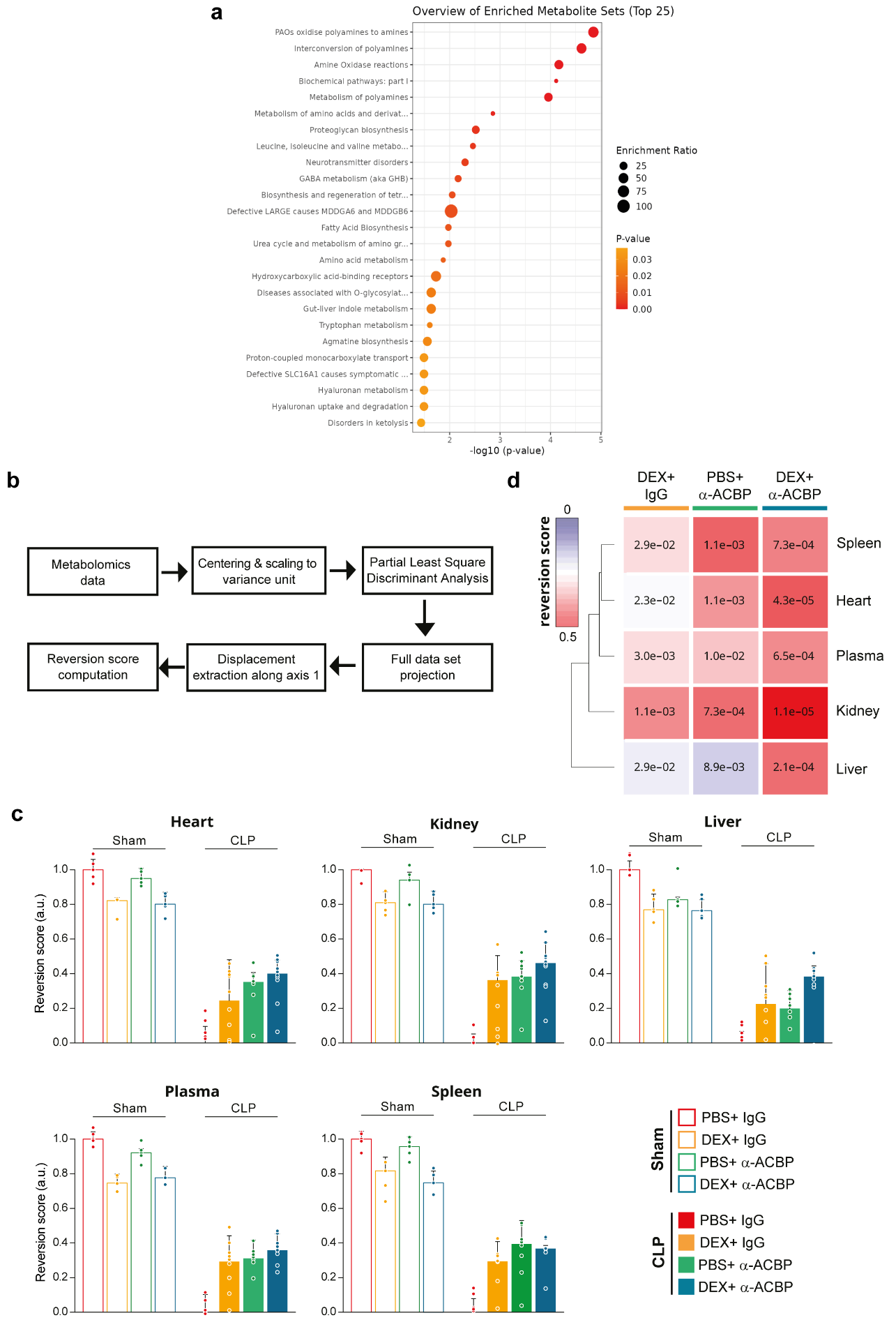


**Supplementary Figure S11. Enrichment pathways and CLP reversion scoring based on PLS-DA of metabolomic profiles. (a)** Enrichment pathways of differentially expressed metabolites from **Supplementary Table S5** measured in the heart, kidney, liver, plasma, and spleen that were induced by CLP and corrected by different treatments: α-ACBP, DEX, or both. **(b)** Workflow summarizing the steps used to calculate the reversion scores. **(c)** Metabolomic data were projected onto the first two PLS-DA components, with component 1 maximizing the separation between control (Sham+PBS/IgG) and disease (CLP+PBS/IgG) groups. Normalized displacements along the primary axis (reversion scores) are summarized in a bar chart showing group medians and median absolute deviations. Negative values were truncated at zero for improved visualization. **(d)** Median reversion scores of CLP groups from panel **(c)** are displayed as a heatmap, with p-values (Mann–Whitney test versus the disease group) indicated.

**Supplementary Figure S12**


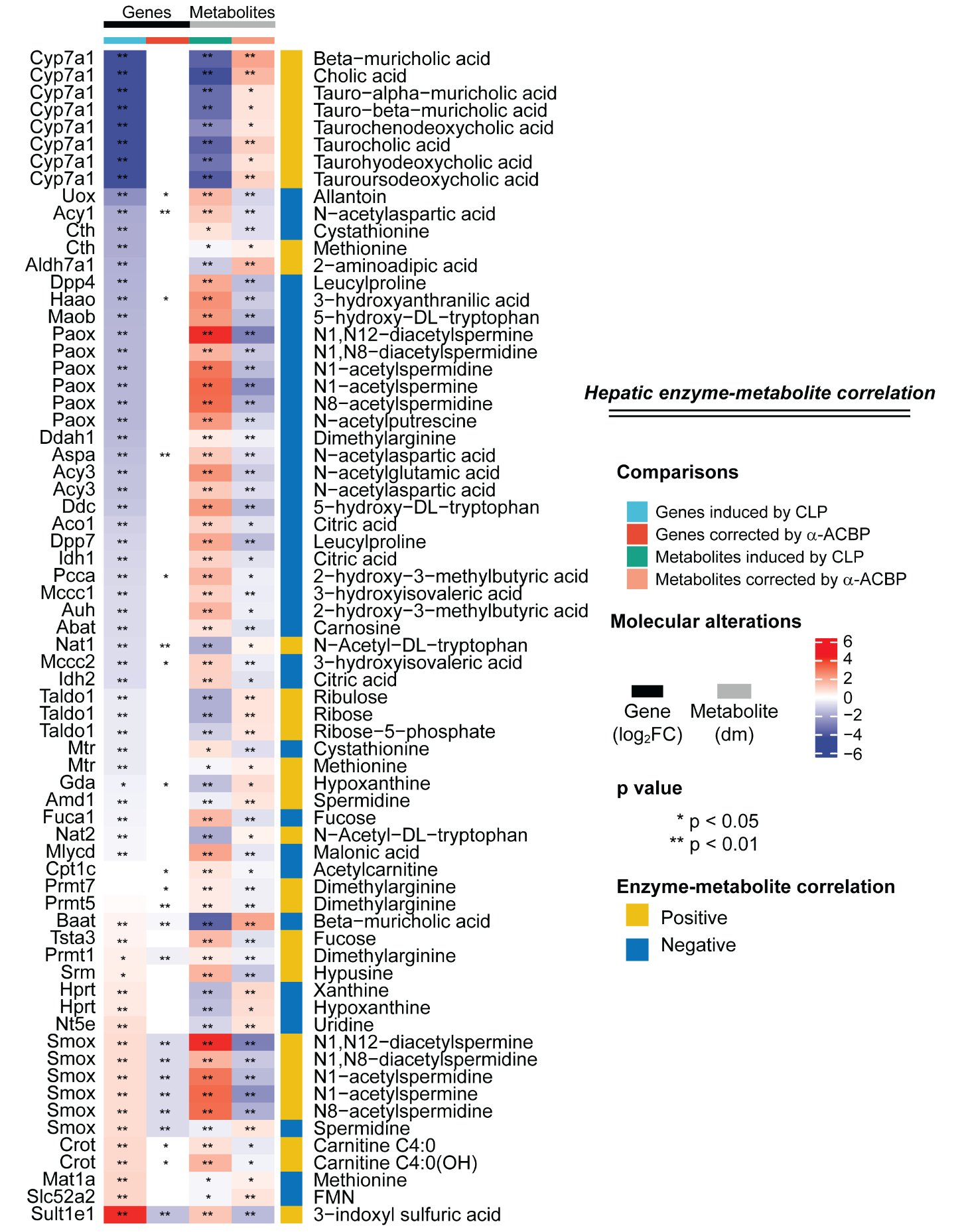


**Supplementary Figure S12. Enzyme–metabolite alterations and correlations in liver during CLP-induced sepsis under anti-ACBP/DBI treatment.** Heatmaps show differential expressions of enzymes and their associated metabolites across four experimental comparisons. Colors represent molecular alterations (genes: log_2_FC; metabolites: dm), with red indicating upregulation and blue indicating downregulation. Right annotation bars denote enzyme–metabolite correlations (yellow, positive; blue, negative). These analyses highlight coordinated transcriptional and metabolic remodeling during sepsis and the modulatory effects of α-ACBP on hepatic metabolism. *p < 0.05; **p < 0.01.

**Supplementary Figure S13**


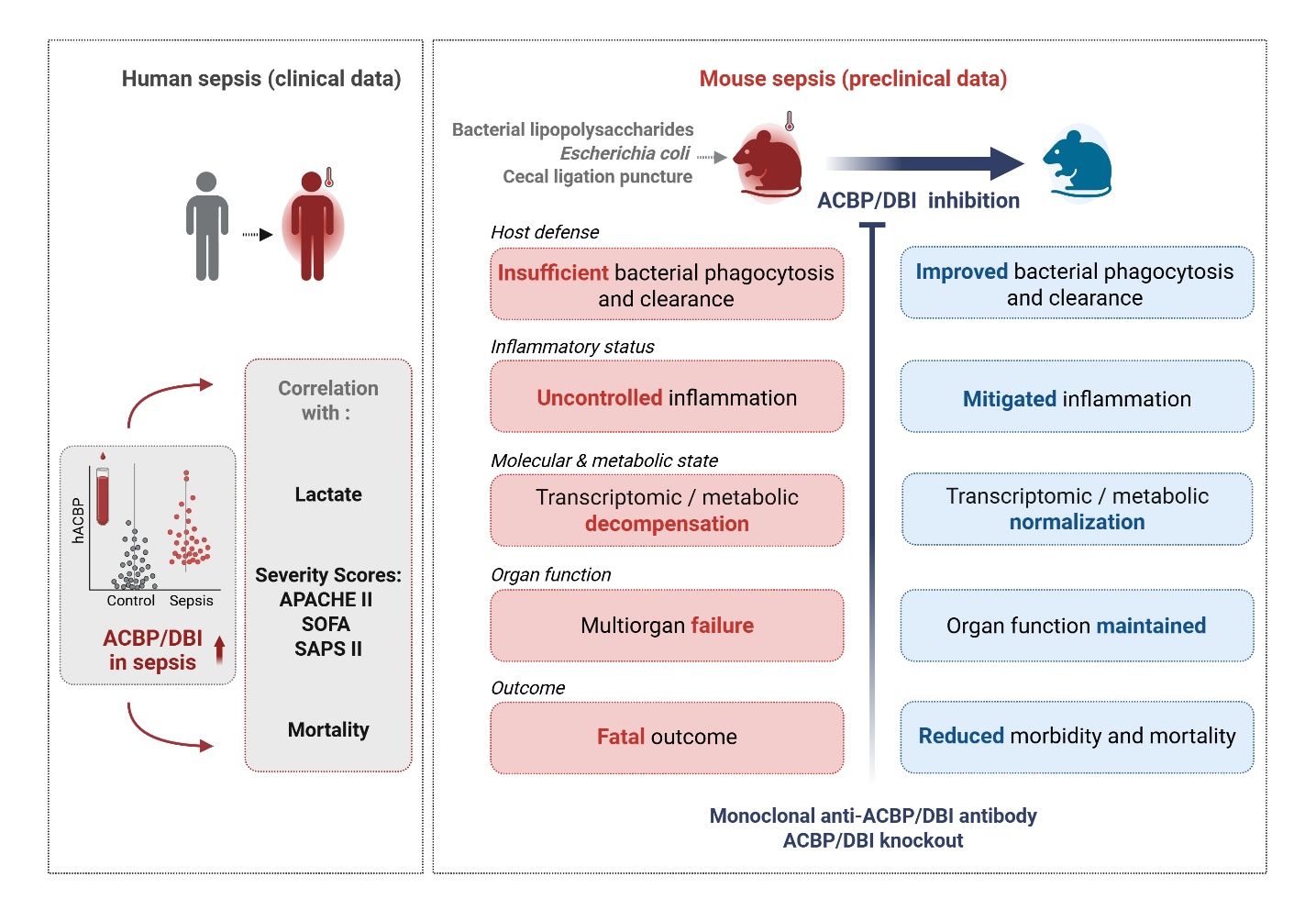


**Supplementary Figure S13. Role of ACBP/DBI in sepsis and effects of its** **neutralization**.
ACBP/DBI is elevated in human sepsis and correlates with severity and mortality. In mouse models, its blockage improves bacterial clearance, reduces inflammation, normalizes metabolism, and protects organ function, lowering mortality and morbidity. Figure generated with ‘BioRender,com’.

**Supplementary Table S1**

| **Variable** | **Control** | **Sepsis** | **Septic Shock** |
| --- | --- | --- | --- |
| Alanine aminotransferase | 30.56 ± 10.67# | 99.33 ± 73.48 | 246.80 ± 173.21# |
| Albumin | 29.96 ± 1.17# | 29.30 ± 5.12 | 19.12 ± 0.98# |
| Alkaline phosphatase | 58.61 ± 4.33 | 79.75 ± 21.47 | 135.11 ± 31.16 |
| Aspartate aminotransferase | 41.72 ± 11.55#* | 146.33 ± 86.96* | 549.65 ± 409.69# |
| Basophils (%) | 0.19 ± 0.03# | 0.10 ± 0.04 | 0.13 ± 0.04# |
| C reactive protein | 14.46 ± 4.42#* | 128.43 ± 26.63* | 223.72 ± 26.49# |
| Calcium | 3.88 ± 0.09 | 3.75 ± 0.31 | 3.59 ± 0.12 |
| Chloride | 110.17 ± 0.85 | 109.50 ± 3.80 | 113.40 ± 2.25 |
| Creatine kinase | 145.53 ± 35.18 | 407.50 ± 350.51 | 738.21 ± 315.84 |
| Creatine kinase MB | 2.13 ± 0.34# | 2.62 ± 0.71 | 12.32 ± 2.88# |
| Creatinine | 1.01 ± 0.20#* | 2.52 ± 0.15* | 2.02 ± 0.34# |
| hACBP | 55.25 ± 24.10#* | 162.77 ± 58.31* | 272.75 ± 72.20# |
| Direct bilirubin | 0.24 ± 0.04 | 0.32 ± 0.12 | 0.55 ± 0.19 |
| Eosinophils | 0.54 ± 0.16* | 0.00 ± 0.00* | 1.16 ± 0.74 |
| γ-glutamyl transferase | 24.67 ± 2.45 | 107.50 ± 73.33 | 96.84 ± 34.13 |
| Glucose | 223.06 ± 50.24# | 148.00 ± 10.62 | 140.68 ± 11.28# |
| Hematocrit | 33.47 ± 0.97 | 39.35 ± 3.80 | 31.32 ± 1.34 |
| Hemoglobin | 11.03 ± 0.37 | 13.20 ± 1.42 | 10.23 ± 0.42 |
| Lactate | 0.98 ± 0.26#* | 1.96 ± 0.42* | 4.13 ± 0.63# |
| Lymphocytes (%) | 13.19 ± 2.87* | 4.22 ± 1.31* | 10.09 ± 2.01 |
| Magnesium | 1.26 ± 0.07# | 1.55 ± 0.12 | 1.58 ± 0.07# |
| Monocytes (%) | 6.74 ± 0.80 | 5.72 ± 2.38 | 6.42 ± 1.41 |
| Myoglobin | 434.26 ± 130.47 | 1976.35 ± 1759.18 | 2231.14 ± 675.15 |
| Neutrophils (%) | 78.94 ± 3.18 | 87.30 ± 3.16 | 80.02 ± 2.93 |
| Phosphate | 2.99 ± 0.16#* | 6.00 ± 0.85* | 3.96 ± 0.36# |
| Platelets | 162.91 ± 16.43 | 134.25 ± 23.58 | 143.25 ± 20.94 |
| Potassium | 3.71 ± 0.12 | 4.07 ± 0.33 | 3.76 ± 0.20 |
| SASP II | 27.39 ± 2.57#* | 40.75 ± 5.88* | 62.40 ± 5.10# |
| Sodium | 140.28 ± 0.56# | 140.75 ± 2.17 | 145.15 ± 1.93# |
| Total bilirubin | 0.71 ± 0.07 | 0.92 ± 0.25 | 1.12 ± 0.27 |
| Total protein | 56.67 ± 1.54# | 47.43 ± 1.01 | 45.05 ± 2.35# |
| Cardiac Troponin I | 0.02 ± 0.00#* | 0.04 ± 0.01* | 0.40 ± 0.13# |
| Urea | 31.56 ± 3.40#* | 101.50 ± 14.54* | 89.00 ± 9.63# |
| White blood cell count | 11.42 ± 1.05* | 21.73 ± 4.05* | 12.90 ± 2.86 |
| Age | 68.78 ± 2.93 | 60.75 ± 4.19 | 67.14 ± 3.45 |
| eGFR | 81.28 ± 6.96#* | 25.39 ± 3.59* | 52.44 ± 9.40# |

**Supplementary Table S1.** Clinical description of the patients included in the discovery cohort (n=43). Data expressed as mean ± SEM. *p ≤ 0.05 Control vs Sepsis. #p ≤ 0.05 Control vs Septic Shock. Abbreviations: eGFR: estimated glomerular filtration rate.

**Supplementary Table S2**

| **Variable** | **Control** | **Sepsis** | **Septic Shock** | **SIRS** |
| --- | --- | --- | --- | --- |
| APACHE_II |  | 20.12 ± 1.40 | 22.10 ± 1.08 | 13.44 ± 0.49 |
| CRP |  | 22.19 ± 1.70 | 22.35 ± 1.53 | 5.78 ± 2.50 |
| hACBP | 19.54 ± 4.84#$* | 140.29 ± 18.21* | 149.43 ± 13.85# | 54.06 ± 8.51$ |
| Hematocrit |  | 34.45 ± 0.70 | 32.25 ± 0.58 |  |
| Hemoglobin |  | 11.06 ± 0.23 | 10.21 ± 0.21 |  |
| Lymphocytes (%) |  | 11.44 ± 1.44 | 11.37 ± 1.47 |  |
| Lymphocytes (WB) |  | 902.88 ± 108.33 | 674.52 ± 74.34 |  |
| Lactate |  | 3.26 ± 0.27 | 4.61 ± 0.51 | 1.63 ± 0.10 |
| Leukocytes |  | 13589.95 ± 1573.05 | 13045.57 ± 1342.35 | 15168.90 ± 692.23 |
| MCHC (WB) |  | 32.00 ± 0.14 | 31.64 ± 0.19 |  |
| MCH (WB) |  | 30.38 ± 0.74 | 29.41 ± 0.25 |  |
| Monocytes (%) |  | 5.92 ± 0.49 | 6.20 ± 0.81 |  |
| Monocytes (WB) |  | 611.80 ± 60.79 | 554.33 ± 58.22 |  |
| MPV (WB) |  | 11.09 ± 0.10 | 11.03 ± 0.15 |  |
| Neutrophils (WB) |  | 10978.56 ± 972.41 | 10657.20 ± 855.51 |  |
| Neutrophils (%) |  | 80.06 ± 1.73 | 79.00 ± 1.93 |  |
| Procalcitonin |  | 29.30 ± 6.25 | 31.99 ± 4.98 |  |
| PaFIO_2_ |  | 212.61 ± 27.77 | 174.07 ± 20.29 |  |
| RBC count |  | 3731621621621.62 ± 77209686150.05 | 3518389830508.47 ± 68831324790.94 |  |
| SOFA |  | 4.97 ± 0.21 | 8.21 ± 0.28 | 4.28 ± 0.23 |
| SOFA-CV |  | 0.97 ± 0.04 | 2.22 ± 0.12 |  |
| SaFIO_2_ |  | 349.81 ± 13.17 | 326.62 ± 14.04 |  |

**Supplementary Table S2.** Clinical description of the patients included in the validation cohort (n=424). Blank cells indicate no available data for that group. Data expressed as mean ± SEM. *p ≤ 0.05 Control vs Sepsis, # p ≤ 0.05 Control vs Septic Shock, ! p ≤ 0.05 Sepsis vs Septic Shock, $ p ≤ 0.05 Control vs SIRS. Abbreviations: SIRS: Systemic Inflammatory Response Syndrome; WB: whole blood; CRP: C-reactive protein; MCH: mean corpuscular hemoglobin; MCHC: mean corpuscular hemoglobin concentration; MPV: mean platelet volume; RBC: red blood cells; APACHE II: Acute Physiology and Chronic Health Evaluation II; SOFA: Sequential Organ Failure Assessment; SOFA-CV: SOFA cardiovascular subscore.

**Supplementary Table S3**

| **Variable** | **CTR + IgG** | **CTR + α-ACBP** | **LPS + IgG** | **LPS + α-ACBP** |
| --- | --- | --- | --- | --- |
| Heart Rate | 594.93 ± 9.93* | 552.54 ± 26.32 | 410.69 ± 5.59#* | 442.14 ± 5.27# |
| Diameter;s | 1.89 ± 0.09* | 2.18 ± 0.06 | 2.63 ± 0.17* | 2.24 ± 0.10 |
| Diameter;d | 3.28 ± 0.08 | 3.63 ± 0.08 | 3.39 ± 0.15 | 3.20 ± 0.09 |
| Volume;s | 11.30 ± 1.34* | 15.98 ± 1.15 | 26.96 ± 4.83* | 17.47 ± 1.84 |
| Volume;d | 43.66 ± 2.63 | 55.77 ± 2.97 | 48.44 ± 5.43 | 41.63 ± 2.85 |
| Stroke Volume | 32.36 ± 1.73* | 39.78 ± 2.04 | 21.47 ± 1.21* | 24.16 ± 1.72 |
| Ejection Fraction | 74.31 ± 2.04* | 71.40 ± 0.97 | 46.71 ± 3.05#* | 58.66 ± 2.80# |
| Fractional Shortening | 42.30 ± 1.85* | 39.98 ± 0.78 | 22.87 ± 1.68#* | 30.39 ± 1.89# |
| Cardiac Output | 19.28 ± 1.19* | 21.85 ± 0.97 | 8.79 ± 0.46* | 10.71 ± 0.82 |
| LV Mass | 149.16 ± 6.00 | 141.70 ± 9.64 | 152.49 ± 5.44 | 155.14 ± 4.43 |
| LV Mass Cor | 119.33 ± 4.80 | 113.36 ± 7.71 | 121.99 ± 4.36 | 124.11 ± 3.54 |
| LVAW;s | 1.78 ± 0.05* | 1.56 ± 0.05 | 1.44 ± 0.04* | 1.56 ± 0.06 |
| LVAW;d | 1.15 ± 0.02 | 0.96 ± 0.04 | 1.10 ± 0.04 | 1.15 ± 0.05 |
| LVPW;s | 1.65 ± 0.08 | 1.51 ± 0.05 | 1.43 ± 0.10 | 1.60 ± 0.04 |
| LVPW;d | 1.18 ± 0.05 | 1.08 ± 0.07 | 1.21 ± 0.08 | 1.30 ± 0.04 |
| LVRI | 45.48 ± 1.38 | 39.10 ± 2.78 | 45.67 ± 2.52 | 48.62 ± 1.37 |
| LV mass index | 1.78 ± 0.08 | 1.72 ± 0.10 | 1.85 ± 0.07 | 1.78 ± 0.05 |
| Cardiac Index | 230.11 ± 12.98* | 266.01 ± 13.57 | 106.73 ± 5.28* | 122.76 ± 8.84 |
| end diastolic volume index | 0.52 ± 0.03 | 0.68 ± 0.04 | 0.59 ± 0.06 | 0.48 ± 0.03 |

**Supplementary Table S3.** Echocardiographic parameters in mice treated with α-ACBP at 12h after LPS challenge (20 mg/kg body weight). Data expressed as mean ± SEM (n=5-10 mice per group). *p ≤ 0.05 for CTR + IgG vs LPS + IgG . # p ≤ 0.05 for LPS + IgG vs LPS + α-ACBP. Abbreviations: Diameter;s: LV internal diameter in systole; Diameter;d: LV internal diameter in diastole; Volume;s: LV end-systolic volume; Volume;d: LV end-diastolic volume; LV Mass: estimated LV muscle mass; LV Mass Cor: LV mass corrected for body weight; LVAW;s: LV anterior wall thickness in systole; LVAW;d: LV anterior wall thickness in diastole; LVPW;s: LV posterior wall thickness in systole; LVPW;d: LV posterior wall thickness in diastole; LVRI: left ventricular remodeling index; LV Mass Index: LV mass normalized to body weight; End Diastolic Volume Index: end-diastolic volume normalized to body weight.

**Supplementary Table S4**

| **Variable** | **Sham +**  **PBS/IgG** | **Sham +**  **DEX/IgG** | **Sham +**  **PBS/α-ACBP** | **Sham +**  **DEX/α-ACBP** | **CLP +**  **PBS/IgG** | **CLP +**  **DEX/IgG** | **CLP +**  **PBS/α-ACBP** | **CLP +**  **DEX/α-ACBP** |
| --- | --- | --- | --- | --- | --- | --- | --- | --- |
| Heart Rate | 548.97 ± 18.35 | 592.52 ± 15.88 | 530.98 ± 11.59 | 568.10 ± 13.35 | 513.10 ± 10.28 | 495.13 ± 12.56 | 519.78 ± 9.95 | 510.18 ± 13.75 |
| Diameter;s | 2.01 ± 0.12* | 1.86 ± 0.11 | 2.06 ± 0.04 | 1.74 ± 0.08 | 1.17 ± 0.12&* | 1.43 ± 0.12 | 1.47 ± 0.11 | 1.64 ± 0.12& |
| Diameter;d | 3.40 ± 0.08* | 3.27 ± 0.11 | 3.49 ± 0.04 | 3.15 ± 0.07 | 2.59 ± 0.11#&* | 2.94 ± 0.08# | 2.71 ± 0.11 | 3.10 ± 0.11& |
| Volume;s | 13.69 ± 1.69* | 11.31 ± 1.56 | 13.94 ± 0.62 | 9.29 ± 0.99 | 4.18 ± 1.04&* | 6.59 ± 1.17 | 7.02 ± 1.32 | 9.00 ± 1.30& |
| Volume;d | 47.78 ± 2.54* | 43.91 ± 3.45 | 50.65 ± 1.44 | 39.67 ± 2.06 | 25.50 ± 2.46#&* | 34.10 ± 2.23# | 28.62 ± 2.63 | 39.42 ± 3.24& |
| Stroke Volume | 34.09 ± 1.19* | 32.60 ± 1.94 | 36.70 ± 0.99 | 30.39 ± 1.63 | 21.32 ± 1.66#&* | 27.51 ± 1.55# | 21.59 ± 1.67 | 30.43 ± 2.18& |
| Ejection Fraction | 72.35 ± 2.51* | 75.45 ± 1.91 | 72.64 ± 0.72 | 76.77 ± 2.02 | 86.08 ± 2.39$&* | 82.36 ± 2.53 | 78.02 ± 2.73$ | 79.22 ± 2.05& |
| Fractional Shortening | 41.21 ± 2.35* | 43.59 ± 1.85 | 40.98 ± 0.62 | 44.71 ± 1.91 | 55.91 ± 2.99$&* | 52.26 ± 3.14 | 46.76 ± 2.68$ | 48.39 ± 2.69& |
| Cardiac Output | 18.62 ± 0.67* | 19.16 ± 0.98 | 19.47 ± 0.68 | 17.23 ± 0.94 | 10.99 ± 0.89#&* | 13.62 ± 0.89# | 11.20 ± 0.87 | 15.34 ± 1.07& |
| LV Mass | 142.20 ± 6.32 | 149.58 ± 6.50 | 147.73 ± 6.88 | 129.95 ± 4.17 | 148.29 ± 4.45#$& | 128.84 ± 5.43# | 131.93 ± 6.28$ | 128.54 ± 3.98& |
| LV Mass Cor | 113.76 ± 5.05 | 119.66 ± 5.20 | 118.18 ± 5.50 | 103.96 ± 3.34 | 118.63 ± 3.56#$& | 103.07 ± 4.35# | 105.54 ± 5.02$ | 102.83 ± 3.19& |
| LVAW;s | 1.64 ± 0.05* | 1.68 ± 0.03 | 1.55 ± 0.03 | 1.70 ± 0.05 | 2.02 ± 0.06#$&* | 1.80 ± 0.07# | 1.77 ± 0.04$ | 1.70 ± 0.06& |
| LVAW;d | 1.06 ± 0.03* | 1.06 ± 0.03 | 0.95 ± 0.03 | 1.06 ± 0.05 | 1.36 ± 0.05#$&* | 1.13 ± 0.03# | 1.18 ± 0.04$ | 1.07 ± 0.04& |
| LVPW;s | 1.50 ± 0.06* | 1.72 ± 0.05 | 1.67 ± 0.04 | 1.61 ± 0.05 | 1.95 ± 0.08$&* | 1.80 ± 0.05 | 1.76 ± 0.07$ | 1.67 ± 0.07& |
| LVPW;d | 1.13 ± 0.06* | 1.28 ± 0.05 | 1.23 ± 0.06 | 1.16 ± 0.04 | 1.47 ± 0.06#&* | 1.21 ± 0.05# | 1.36 ± 0.07 | 1.18 ± 0.06& |
| LVRI | 42.07 ± 2.12* | 45.86 ± 1.68 | 42.27 ± 1.67 | 41.56 ± 1.91 | 58.49 ± 2.72#$&* | 44.07 ± 1.69# | 49.04 ± 2.03$ | 42.58 ± 2.17& |
| LV mass index | 1.79 ± 0.08 | 1.87 ± 0.09 | 1.83 ± 0.09 | 1.60 ± 0.05 | 1.86 ± 0.04 | 1.67 ± 0.06 | 1.71 ± 0.07 | 1.70 ± 0.05 |
| Cardiac Index | 234.37 ± 9.07* | 239.07 ± 11.96 | 242.06 ± 9.88 | 212.02 ± 11.19 | 138.14 ± 11.44#&* | 178.74 ± 13.38# | 144.45 ± 11.02 | 203.68 ± 14.15& |
| end diastolic volume index | 0.60 ± 0.03* | 0.55 ± 0.04 | 0.63 ± 0.02 | 0.49 ± 0.02 | 0.32 ± 0.03#&* | 0.44 ± 0.03# | 0.37 ± 0.03 | 0.53 ± 0.04& |

**Supplementary Table S4.** Ecocardiographic data measured at 24h after CLP surgery in mice treated with α-ACBP, DEX or combination. Data expressed as mean ± SEM (n=10-20 mice per group). *p ≤ 0.05 for sham + IgG-PBS vs CLP + IgG-PBS. # p ≤ 0.05 for CLP + IgG-PBS vs CLP + IgG-DEX. $ p ≤ 0.05 for CLP + IgG-PBS vs CLP + αACBP-PBS. & p ≤ 0.05 for CLP + IgG-PBS vs CLP + αACBP-DEX. Abbreviations: Diameter;s: LV internal diameter in systole; Diameter;d: LV internal diameter in diastole; Volume;s: LV end-systolic volume; Volume;d: LV end-diastolic volume; LV Mass: estimated LV muscle mass; LV Mass Cor: LV mass corrected for body weight; LVAW;s: LV anterior wall thickness in systole; LVAW;d: LV anterior wall thickness in diastole; LVPW;s: LV posterior wall thickness in systole; LVPW;d: LV posterior wall thickness in diastole; LVRI: left ventricular remodeling index; LV Mass Index: LV mass normalized to body weight; End Diastolic Volume Index: end-diastolic volume normalized to body weight.

**Supplementary Table S5**


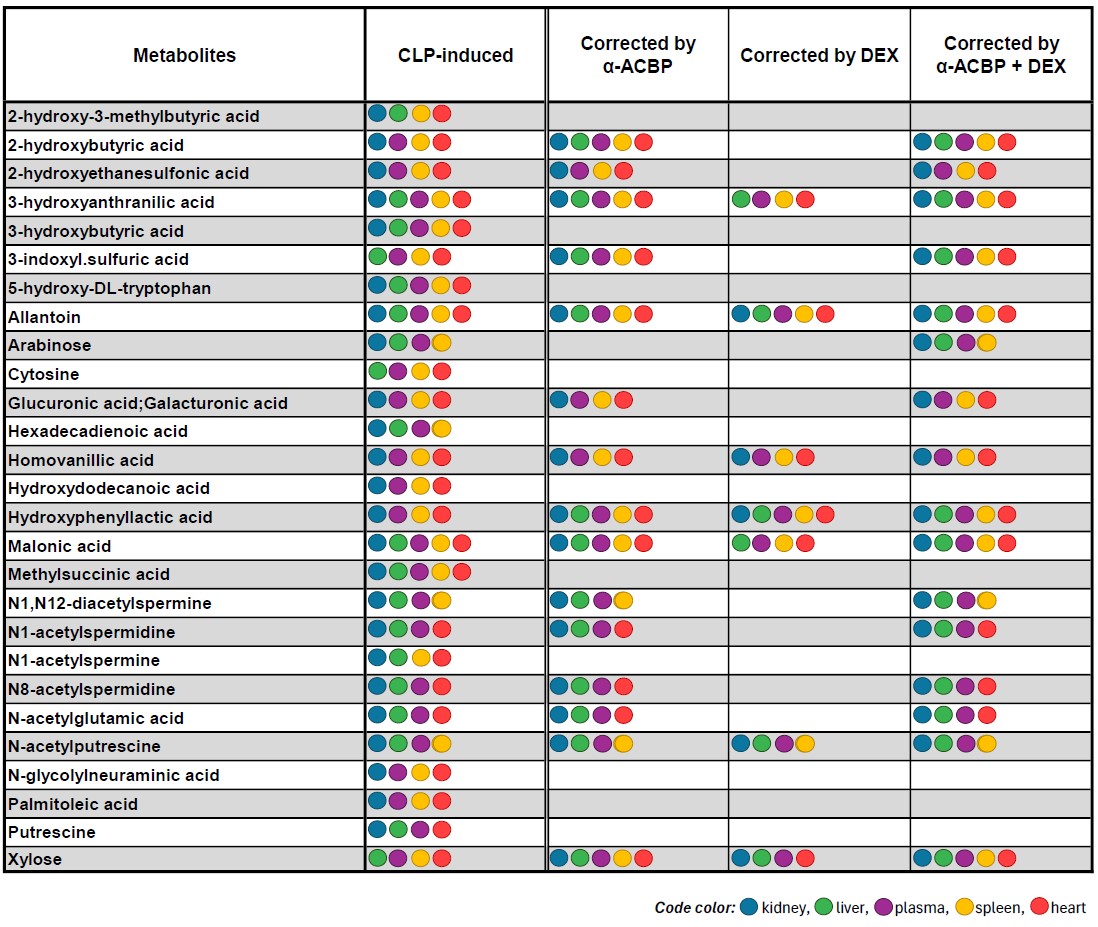


**Supplementary Table S5.** Metabolites measured in the heart, kidney, liver, plasma, and spleen that were induced by CLP and corrected by different treatments: α-ACBP, DEX, or both.

**Supplementary Table S6**

| **Oligonucleotides** | **Source** | **Identifier** |
| --- | --- | --- |
| *Mouse RT-qPCR* |  |  |
| Primer: *36b4*  F: 5′ -ACTGGTCTAGGACCCGAGAAG-3′  R: 5′ -TCCCACCTTGTCTCCAGTCT-3′ | Sigma-Aldrich | N/A |
| Primer: *Dbi*  F: 5′-GAATTTGACAAAGCCGCTGAG-3′  R: 5′-CCCACAGTAGCTTGTTTGAAGTG-3′ | Sigma-Aldrich | N/A |
| Primer: *Nlrp3*  F: 5′- ATTACCCGCCCGAGAAAGG -3′  R: 5′- TCGCAGCAAAGATCCACACAG -3′ | Sigma-Aldrich | N/A |
| Primer: *Il6*  [Mm00446190_m1](https://na01.safelinks.protection.outlook.com/?url=https%3A%2F%2Fwww.thermofisher.com%2Ftaqman-gene-expression%2Fproduct%2FMm00446190_m1%3FCID%3D%26ICID%3D%26subtype%3D&data=05%7C02%7C%7Cd5146ebef6764565006c08dc587268a3%7C84df9e7fe9f640afb435aaaaaaaaaaaa%7C1%7C0%7C638482495638778638%7CUnknown%7CTWFpbGZsb3d8eyJWIjoiMC4wLjAwMDAiLCJQIjoiV2luMzIiLCJBTiI6Ik1haWwiLCJXVCI6Mn0%3D%7C0%7C%7C%7C&sdata=i2th%2F62f7hH2RO5nYP4N4PJckCSeidMlvJ%2B6BTVNT3I%3D&reserved=0) | Thermo-Fisher | Catalog #4331182 |
| Primer: *Ccl2*  Mm00441242_m1 | Thermo-Fisher | Catalog #4331182 |
| Primer: *Il1b*  Mm00434228_m1 | Thermo-Fisher | Catalog #4331182 |
| Primer: *Tnf*  Mm00443258_m1 | Thermo-Fisher | Catalog #4331182 |
| Primer: *Il10*  Mm01288386_m1 | Thermo-Fisher | Catalog #4331182 |
| Primer: *Ifng*  Mm01168134_m1 | Thermo-Fisher | Catalog #4331182 |
| Primer: *Ppia*  Mm02342430_g1 | Thermo-Fisher | Catalog #4331182 |

**Supplementary Table S6.** Primer sequences used for RT-qPCR (related to Fig. 2e).
